# Ligand-free Pd-catalyzed highly selective arylation of activated and unactivated alkenes *via* oxidative and reductive heck coupling†

**DOI:** 10.1039/d3ra08186a

**Published:** 2024-02-21

**Authors:** Mixiang Tian, Qinghong Cui, Qiuling Xu, Wenwen Wu, Yuxian Wang, Kun Wei, Ruifen Sun, Junliang Wang

**Affiliations:** a Center for Scientific Research, Yunnan University of Chinese Medicine Kunming Yunnan 650500 P. R. China wangjunliangedu@163.com sunruifen@ynutcm.edu.cn; b School of Chemical Science and Technology, Yunnan University Kunming Yunnan 650500 P. R. China

## Abstract

In this work, an eco-friendly, green, efficient approach for oxidative and reductive Heck–Mizoroki (HM) reactions was developed, which offered acceptable yields from first-pass experiments. Mono-arylation was achieved without the use of ligands, directing groups, or prefunctionalized alkenes. Considering mild reaction conditions, good functional group compatibility, and great regioselectivity, the method can find broad applications in novel medicine and material development and discovery processes.

## Abstract 2

We developed a solvent-free, mild, and efficient protocol for Heck–Mizoroki reactions under ultrasonic irradiation in open air, which would be predictable and robust using a range of substrates.

## Graphical abstract

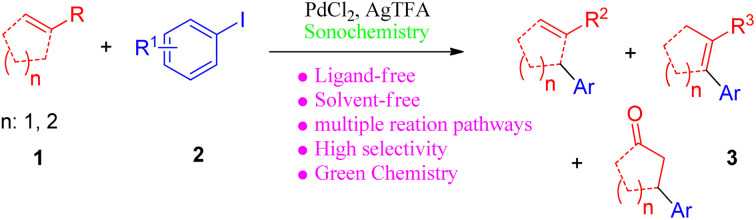

## Introduction

**Scheme 1:**
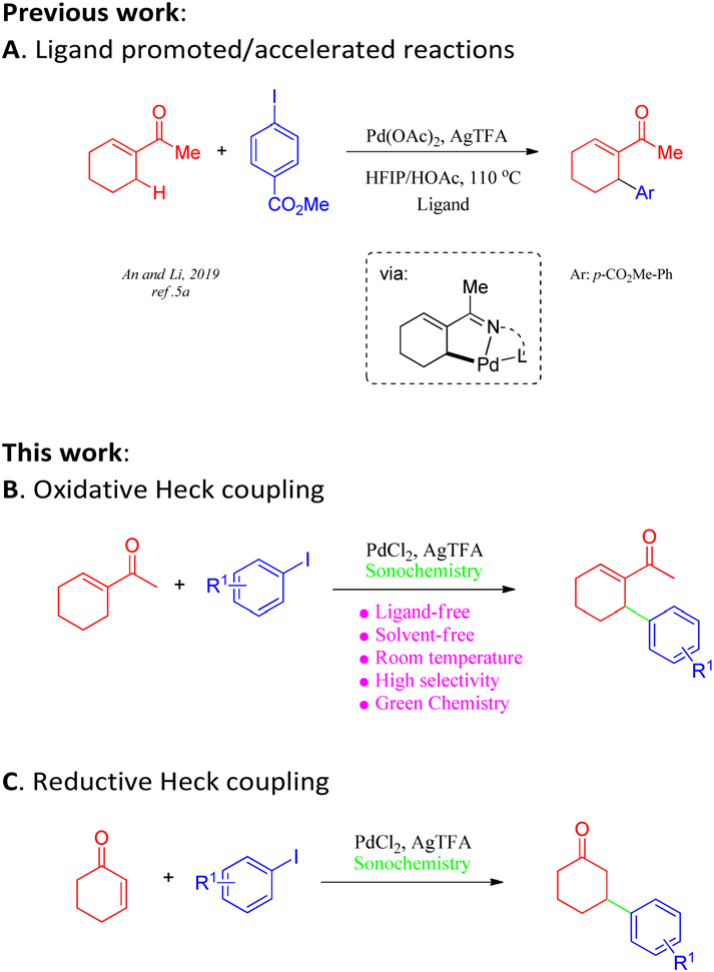
(A) Ligand promoted/accelerated coupling reactions with α,β-unsaturated ketone. (B) Ligand-free Pd-catalyzed oxidative heck coupling reactions. (C) Ligand-free Pd-catalyzed reductive heck coupling reactions.

Approaches employing Heck coupling have emerged as some of the most powerful tools for modern organic syntheses owing to their high efficiency and widespread applications in the synthesis of pharmaceutical drugs and functional materials.^1^ Although ground-breaking advances in metal-catalysed reactions have been reported in the past two decades, the pursuit of scalable, operationally simple protocols for the Heck–Mizoroki (HM) reaction has always been challenging. It is particularly true that drawbacks such as harsh reaction conditions and low yields greatly hamper its large-scale practical application, especially in the pharmaceutical industry.^2^ Attempts to overcome these drawbacks include attempted syntheses of efficient catalysts;^3^ the utilization of solvent-free reaction conditions; and the application of non-classical energy sources such as microwave irradiation, high pressure, and mechanochemical techniques.^4^ Despite significant advances in acyclic aliphatic alkenes and cycloenones (Scheme 1A),^5*a*–*d*^ the synthesis of cyclic olefins involving oxidative or reductive Heck coupling continues to be particularly challenging. Additionally, chemo-selectivity is another commonly faced problem within directing-group-assisted C–H olefination.^5^ Hence, it is of great synthetic value to develop a practical methodology to address these long-standing issues.

In recent decades, the application of green methodologies that are environmentally friendly and can minimize chemical disposal and energy consumption have become the goals of organic chemists. One of the green chemistry techniques is ultrasound irradiation. Ultrasound technology has recently been applied to accelerate a large number of organic reactions under aqueous and non-aqueous conditions.^6^ In particular, such techniques often provide an ameliorative, sustainable system with solvent-free conditions^7^ for the improvement of traditional transformations^8^ and, most importantly, alter chemical reactivity as well as selectivity.^9^ The recent demand for eco-friendly chemical processes has led to the development of several clean and efficient protocols.^10^ Furthermore, a reliable and robust procedure for Heck reactions would be of considerable value. Herein, we report an ultrasound-assisted methodology for oxidative or reductive Heck coupling under solvent-free conditions at room temperature in open air, which can be applied to a broad range of coupling partners (Scheme 1B and C).

## Results and discussion

To develop a general protocol for the arylation of olefins under mild conditions, a model coupling reaction between 1-cyclohexenyl methyl ketone (1a) and methyl 4-iodobenzoate (2a) in the presence of catalytic Pd(OAc)_2_ and stoichiometric amounts of AgTFA was conducted to screen optimal reaction conditions; the results are shown in Table 1. Only a trace amount of the expected product (3a) was formed when the model coupling reaction was treated with mechanical stirring at room temperature (Table 1, entry 1). However, the application of ultrasound led to a moderate improvement in the yield to 76% (Table 1, entry 2). In this case, when the substrates increased the energy of collision due to a change in the oscillations of the electrical component of the microwave field, it induced the breakage and re-establishment of intermolecular bonds. Next, the screening of silver salts was conducted, and a low yield was observed with Ag_3_PO_4_, AgOAc, Ag_2_O, and Ag_2_CO_3_ (entries 3–6). Further investigations indicated that the desired product was formed in the highest yield with 1.5 equivalents of AgTFA. The yields decreased with a higher or lower concentration of AgTFA (compare entries 7–10 of Table 1). The examination of different palladium catalysts revealed that all the examined reactions led to some conversion, albeit with quite different efficiencies (Table 1, entries 11–15), with PdCl_2_ being the most reactive. When the amount of catalyst was reduced to 0.5 mol%, the yields decreased obviously (Table 1, entry 16–18). In stark contrast, the coupling reaction proceeded less efficiently in solvents such as HFIP (hexafluoroisopropyl alcohol), DMSO, DMF, EtOH, THF, DCM, and Tol (toluene). According to the above results, the optimization study revealed that the production of 3a can be successfully accomplished in high yield (86%) by ultrasonic irradiation of a mixture of 1a and 2a in the absence of the solvent at room temperature in the open air.

**Table 1:** Optimization of the reaction conditions^a^

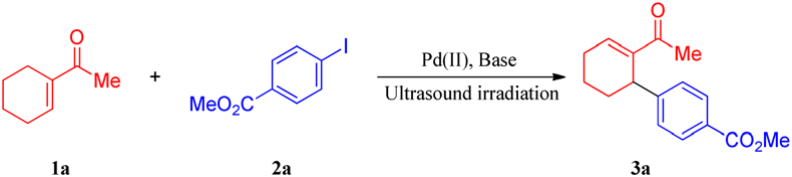			
Entry	Pd source (mol%)	Base (equiv.)	Yield^b^ (%)
1^c^	Pd(Oac)_2_ (10)	AgTFA (1.0)	70
2	Pd(Oac)_2_ (10)	AgTFA (1.0)	76
3	Pd(Oac)_2_ (10)	Ag_3_PO_4_ (1.0)	<10
4	Pd(Oac)_2_ (10)	AgOAc (1.0)	<10
5	Pd(Oac)_2_ (10)	Ag_2_O (1.0)	Trace
6	Pd(Oac)_2_ (10)	Ag_2_CO_3_ (1.0)	<10
7	Pd(Oac)_2_ (10)	AgTFA (0.2)	22
8	Pd(Oac)_2_ (10)	AgTFA (0.6)	61
9	Pd(Oac)_2_ (10)	AgTFA (1.5)	84
10	Pd(Oac)_2_ (10)	AgTFA (2.0)	67
11	Pd(TFA)_2_ (10)	AgTFA (1.5)	68.6
12	Pd(CH_3_CN)_2_Cl_2_ (10)	AgTFA (1.5)	85
13	Pd(PPh_3_)_2_Cl_2_ (10)	AgTFA (1.5)	<10
14	Pd(dppf)Cl_2_ (10)	AgTFA (1.5)	<10
15	**PdCl**_**2**_ **(10)**	**AgTFA (1.5)**	**86**
16	PdCl_2_ (0.5)	AgTFA (1.5)	Trace
17	PdCl_2_ (1.0)	AgTFA (1.5)	13
18	PdCl_2_ (5.0)	AgTFA (1.5)	19
19^d^	PdCl_2_ (10)	AgTFA (1.5)	<30

a Reactions were performed taking reactant 1a (0.45 mmol) and 2a (0.15 mmol) under ultrasound irradiation at room temperature in the open air. Sonication was performed using an ultrasound cleaning bath with a frequency of 40 kHz and a voltage of 220 V.

b Isolated yield.

c Mechanical stirring instead of ultrasound irradiation.

d Solvent (1 mL): HFIP, DMSO, DMF, EtOH, THF, DCM, and Tol.

**Scheme 2:**
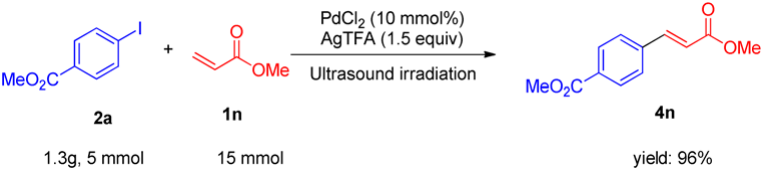
The scale-up Heck coupling reaction.

After determining the optimal conditions, we next investigated the substrate scope of olefins and aryl iodides (Table 2 and Scheme 2). Firstly, the substrate scope of the activated and unactivated olefins was examined for Heck coupling, using methyl 4-iodobenzoate (2a) as the model substrate. It was interesting to find that the Heck coupling of 2a with a variety of substituted cyclic olefins was achieved to afford the allylic products (3a–3e) in excellent to moderate yields, while cyclohexene and 3-methylenedihydrofuran-2(3*H*)-one provided the mixture of double-bond isomers (3f/3g, 3h/3i) with good yields (75% and 73%, respectively). Unexpectedly, α,β-unsaturated ketones, such as cyclohex-2-enone, cyclopent-2-enone, and cyclohept-2-enone, led to the reductive Heck products 3j, 3k, and 3l with moderate yields. Gratifyingly, a wide range of terminal olefins underwent the coupling smoothly, offering the corresponding products in satisfactory yields (up to 96%). In all the cases, only the *E*-isomers (3m–3s) were selectively obtained, and the mono-arylations were also regioselective.

**Table 2:** Scope of olefins

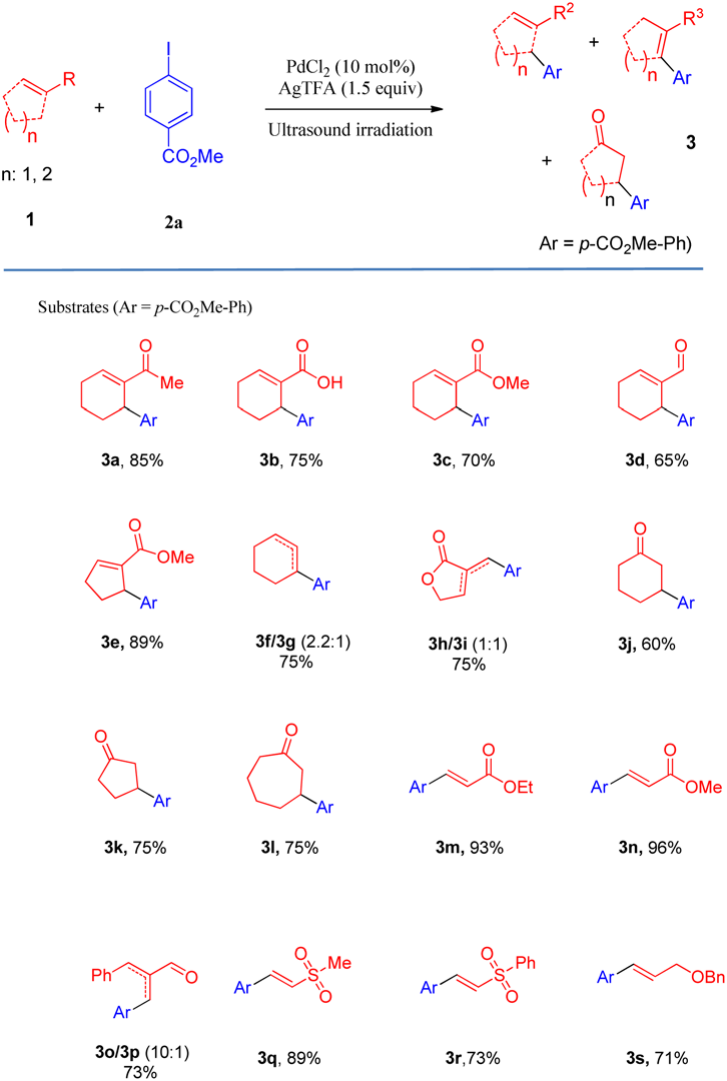

We also surveyed the scope of aryl iodides comprising various types of functional groups. Under mild reaction conditions and using simple operations, catalytic amounts of Pd(Oac)_2_ and stoichiometric amounts of AgTFA effected the transformations of aryl iodides into the corresponding monoarylated product (4a–4ah) in good to excellent yields. The coupling of aryl iodides bearing *ortho*-, *meta*- or *para*-substituent (4a–4u) with methyl acrylate furnished satisfactory yields of the monoarylated products. It is notable that the steric hindrance properties of aryl iodides did not show significant influences on the reactions (4d and 4e), and a phenolic hydroxy group (4f) and protic functional groups (4g) are well tolerated in this process. Not surprisingly, the catalytic system distinguished between iodide and other potential reactive halides (4t and 4u) (Table 3).

**Table 3:** Scope of aryl iodides

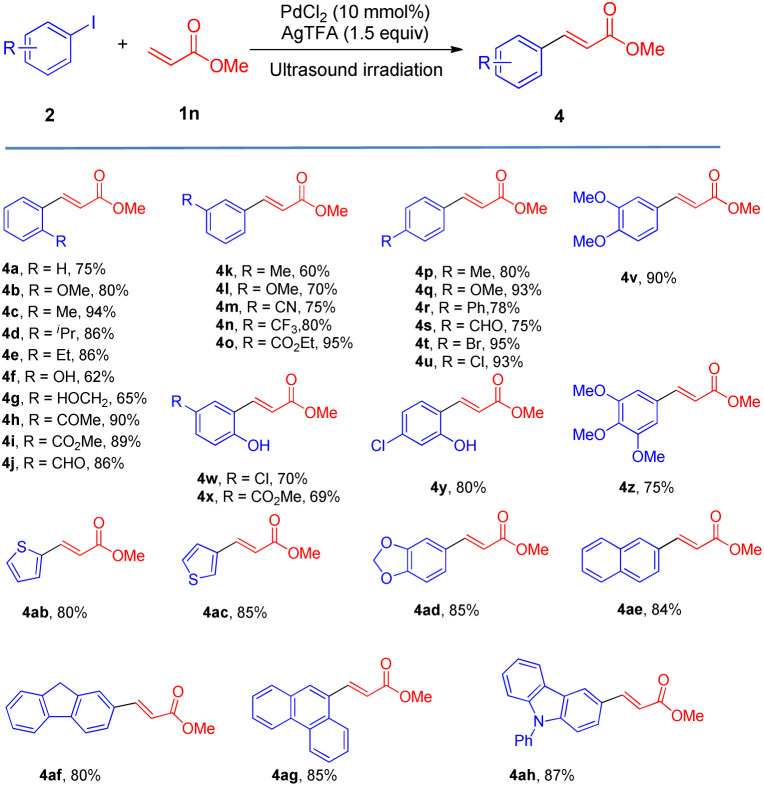

Moreover, this new protocol succeeded with the multi-substituted substrate (4v–4z). Furthermore, heteroaryl iodides and iodine-substituted fused-ring compounds in this process could also give very beneficial yields (4ab–4ah).

In particular, the arylations of olefins could be well scalable under the optimized conditions, which could be up to 1.3 g (Scheme 2). The proposed method offers several remarkable advantages, including shorter reaction time, milder reaction conditions, and reduction of undesired side reactions causing higher yields in comparison to the conventional methods.^11^ These features render our protocol particularly useful for coupling reactions of complex small molecules, which offers an excellent option for establishing a new horizon for Heck-type reactions of olefins.

**Scheme 3:**
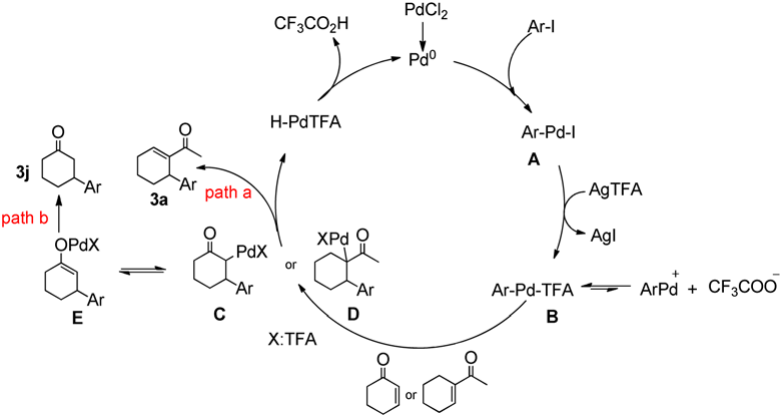
Plausible mechanism.

On the basis of the experimental results (3a, 3j) and literature reports,^12^ two tentative mechanisms for the arylation of arenes are proposed, as shown in Scheme 3. We believe that an initial silver-mediated iodide abstraction from the aryl palladium iodide (A) results in the formation of the transition state ArPdTFA (B). It is presumed that the transition state is followed by an equilibrium involving ArPdTFA, ArPd^+^, and CF_3_COO^−^. ArPdTFA is assumed to be the reactive species instead of ArPd^+^. Insertion of an olefin into the C–Pd bond of ArPdTFA would result in the formation of new alkyl palladium species (C, D). There are two pathways: path a and path b.

In path a, β-hydride elimination of intermediate D gave product 3a, the Pd(0) species was obtained in the reductive elimination of HPdTFA from the β-hydride elimination process.

In path b, enolization of intermediate C generated the palladium enolate E, finally, protonolysis of E furnished the desired β-arylated ketone with regeneration of the Pd catalyst.

## Conclusions

In summary, we developed a solvent-free, mild, and efficient protocol for the Heck–Mizoroki reactions under ultrasonic irradiation in open air, which would be predictable and robust using a range of substrates. Varieties of aryl iodides were tolerated in the reaction with a wide range of olefins and provided the oxidative or reductive Heck products in satisfactory yields even at the gram scale. More importantly, the approach should find broad applications in an industrial environment and the synthesis of ubiquitous structural units in pharmaceuticals.

## Conflicts of interest

There are no conflicts to declare.

## Supplementary Material

RA-014-D3RA08186A-s001
