# Supplementary material for: Ligand-free Pd-catalyzed highly selective arylation of activated and unactivated alkenes via oxidative and reductive heck coupling
Source: RSC Adv. 2024 Feb 21;14(10):6470–5. doi: 10.1039/d3ra08186a (PMC10879848; doi:10.1039/d3ra08186a)
Supplement: RA-014-D3RA08186A-s001 [file RA-014-D3RA08186A-s001.pdf]

Electronic Supplementary Material (ESI) for RSC Advances.
This journal is © The Royal Society of Chemistry 2024

Supporting Information

Ligand-Free Pd-Catalyzed Highly Selective Arylation of Activated and Unactivated Alkenes

Mixiang Tian,[a] Qinghong Cui,[a] Qiuling Xu,[a] Wenwen Wu,[a] Yuxian Wang,[a] Kun Wei,[b] Ruifen Sun[a,*] and Junliang Wang[a,*]

[a] Center for scientific research, Yunnan University of Chinese Medicine, Kunming, 650500 (P. R. China). E-mail: wangjunliangedu@163.com; sunruifen@ynutcm.edu.cn.

[b] School of Chemical Science and Technology, Yunnan University, Kunming, 650091 (P. R. China).

Contents

General Information **S2**

Experimental Section **S3**

1. Optimization of reaction conditions S3
2. General procedure for Heck reactions S5

Gram-scale experiment **S5**

Characterization of products **S6**

Copies of the ^{1}H and ^{13}C NMR spectra **S26**

General Information

Unless otherwise noted, all the reagents (including all Iodobenzene and olefin are known compounds), starting materials were purchased from commercial vendors (e. g. Adamas-beta) and used without further purification. All NMR spectra were acquired on Bruker Advance III 400 MHz spectrometers. ^{1}H NMR chemical shifts were recorded relative to SiMe4 (δ 0.00) or residual protiated solvents ($CDCl_3$: δ 7.26). Multiplicities were given as: s (singlet), d (doublet), t (triplet), q (quartet) and m (multiplet). The number of protons (n) for a given resonance was indicated by nH. Coupling constants were reported as a J value in Hz. ^{13}C NMR chemical shifts were recorded relative to solvent resonance ($CDCl_3$: δ 77.16). High-resolution mass spectra (HRMS) were recorded on an Agilent Mass spectrometer using ESI-TOF. All compounds were measured using infrared spectroscopy using an infrared spectrometer (Zhongke Ruijie Tianjin Technology Co., Ltd.) and subjected to KBr compression. Individual compounds were measured for molecular weight using mass spectrometry 4500 QTRAP (Applied Biosystems, USA). All known products gave satisfactory analytical data by 1H NMR or 13C NMR spectra.

Sonication was performed using an ultrasound cleaning bath (Shanghai guante, China) with a frequency of 40 kHz and voltage of 220 V.

Experimental Section

1. Extended optimization table for HM reactions

Full optimization process for the HM reactions starting from effect of TFA equivalents on reaction yield, variables considered were Ag salt, solvent, Pd catalyst as well as loadings of catalyst. When using $Pd(OAc)_2$ and AgTFA, therefore removing TFA additive from the reaction, there is a positive trend in the yield. Most crucial was the change to AgTFA and Pd catalyst, solvent-free condition.

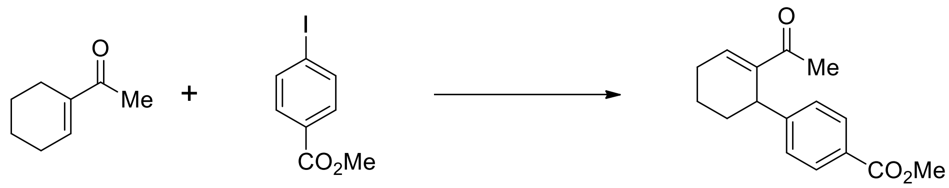

1a **2a** **3a**

Table 1 Evaluation of TFA and Ag salt in HM reactions [a]

Entry	TFA (equiv)	Silver salt (equiv)	Yield (%) [b]
1	TFA (0.0)	AgTFA (1.0)	76
2	TFA (0.5)	AgTFA (1.0)	63
3	TFA (1.0)	AgTFA (1.0)	69
4	TFA (1.5)	AgTFA (1.0)	54
5	TFA (2.0)	AgTFA (1.0)	75.4
6	TFA (0.0)	Ag_3PO_4 (1.0)	<10
7	TFA (0.0)	AgOAc (1.0)	<10
8	TFA (0.0)	Ag_2O (1.0)	trace
9	TFA (0.0)	Ag_2CO_3 (1.0)	<10
10	TFA (0.0)	AgTFA (0.2)	22
11	TFA (0.0)	AgTFA (0.6)	61
12	TFA (0.0)	AgTFA (1.5)	84
13	TFA (0.0)	AgTFA (2.0)	67

[a] Reaction conditions: **2a** (0.15mmol, 1equiv), **1a** (0.45mmol, 3equiv), TFA, Ag salt, $Pd(OAc)_2$ (10 mmol%), under

ultrasonic irradiation for 24 h; [b] Isolated yield.

Table 2 Evaluation of Pd catalyst in HM reactions [a]

Entry	catalyst (mmol%)	Yield (%) [b]
1	Pd $(OAc)_2$ (10)	76
2	Pd $(TFA)_2$ (10)	68.6
3	Pd $(CH_3CN)_2Cl_2$ (10)	85
4	Pd $(PPh_3)_2Cl_2$ (10)	<10
5	Pd (dppf) Cl_2 (10)	<10
6	$PdCl_2$ (10)	86
7	$PdCl_2$ (0.5)	trace
8	$PdCl_2$ (1.0)	13
9	$PdCl_2$ (5.0)	19

[a] Reaction conditions: **2a** (0.15mmol, 1equiv), **1a** (0.45mmol, 3e quiv), AgTFA (1.5 equiv), Pd catalyst, under ultrasonic irradiation for 24 h; [b] Isolated yield.

Table 3 Solvent effects in in HM reactions [a]

Entry	Solvent	Yield [b]
1	HFIP	10
2	DMSO	No reaction
3	DMF	No reaction
4	EtOH	30
5	THF	20

6	DCM	30
7	TOL	20

[a] Reaction conditions: **2a** (0.15 mmol, 1 equiv), **1a** (0.45 mmol, 3equiv), AgTFA(1.5 equiv), $PdCl_2$ (10 mmol%), 1mL solvent, a under ultrasonic irradiation for 24 h; [b] Isolated yield.

To a 5mL reaction tube were added Pd catalyst, silver salt, additive (TFA), **2a** (0.2mmol 1.0eq.) and **1a** (3.0eq.) or solvent (1mL). The tube was then sealed, and the reaction mixture was ultrasonically treated in an ultrasonicator for 24 hours at room temperature. The crude reaction mixture was purified on silica gel using hexanes/EtOAc as the eluent to afford the desired product **3a**.

2. General procedure for the Heck coupling reactions

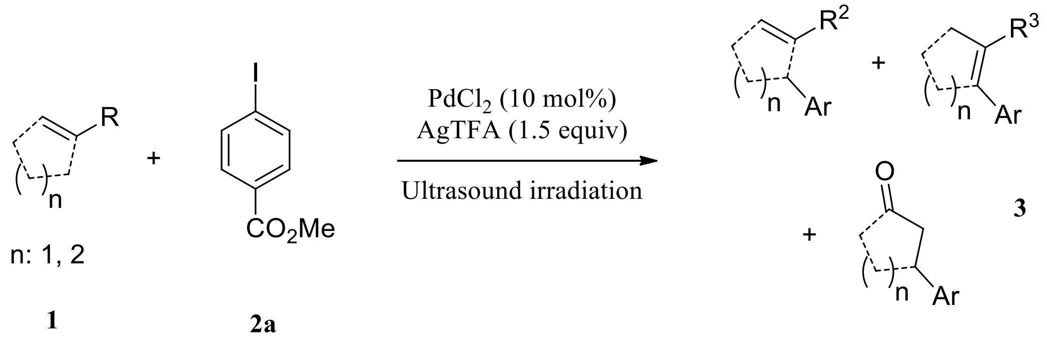

1 **2a** **3**

Ar = *p*-CO_2Me-Ph)

To a 5mL reaction tube were added $PdCl_2$ (10 mmol%), AgTFA (1.5 equiv), aryl iodides (**2**, 0.2 mmol 1.0 equiv) and olefins (**1**, 3.0 equiv). The tube was then sealed, and the reaction mixture was ultrasonically treated in an ultrasonicator for 24 hours at room temperature. The crude reaction mixture was purified on silica gel using hexanes/EtOAc as the eluent to afford the desired product **3**.

Gram-scale experiment

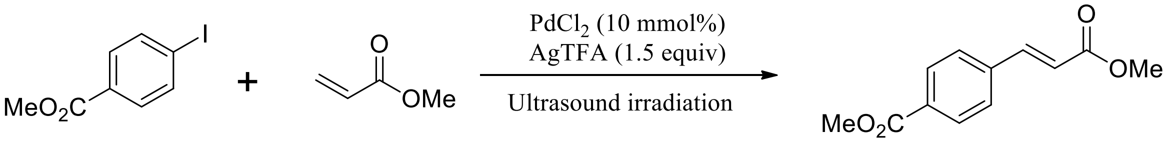

**2a**	**1n**	**4n**
5 mmol	15 mmol	96%

To a 25 mL tube were added $PdCl_2$ (10 mmol%, 88.7 mg), AgTFA (1.66g, 1.5 equiv), methyl 4-iodobenzoate (**2a**, 1.3g, 5mmol, 1.0 equiv) and methyl acrylate (**1n**, 1.35ml, 3.0 equiv), The tube was then sealed, and the reaction mixture was ultrasonically treated in an ultrasonicator for 24 hours at room temperature. The crude reaction mixture was purified on silica gel using hexanes/EtOAc as the eluent to afford the desired product **4n** (yield: 96%).

Characterization of products

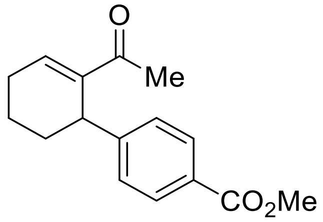

Methyl 6'-acetyl-2',3',4',5'-tetrahydro-[1,1'-biphenyl]-4-carboxylate (3a): This compound was prepared by the general procedure described above and was obtained as white solid (24 h, 85%). R_f (PE: EtOAc = 10:1): 0.5. ESI m/z 281.1150 $[M+Na]^+$. ^{1}H NMR (500 MHz, $CDCl_3$) δ 7.92 (d, J = 9.36 Hz, 2H), 7.19 (d, J = 8.00 Hz, 1H), 7.17 (d, J = 8.22 Hz, 2H), 4.05 (s, 1H), 3.88 (s, 3H), 2.45-2.39 (m, 1H), 2.34-2.3 (m, 1H), 2.25 (s, 3H), 1.95-1.85 (m, 1H), 1.79-1.75 (m, 1H), 1.55-1.45 (m, 2H); ^{13} C NMR (500 MHz, $CDCl_3$) δ 198.16, 167.07, 150.70, 142.57, 140.65, 129.58, 127.71, 51.89, 38.60, 31.01, 26.10, 25.67, 16.95. FT-IR (neat, cm^{-1}): 3419.28, 3385.4, 1763.78, 1716.63, 1652.72, 1182.30, 643.39, 508.90.

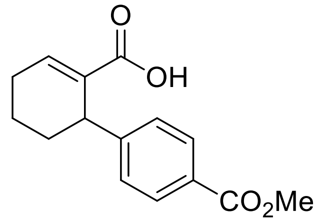

4'-(methoxycarbonyl)-1,4,5,6-tetrahydro-[1,1'-biphenyl]-2-carboxylic acid (3b): This compound was prepared by the general procedure described above and was obtained as white solid (24 h, 75%). R_f (PE: EtOAc = 10:1): 0.5. ESI *m/z* 283.0940 $[M+Na]^+$. ^{1} H NMR (500 MHz, $CDCl_3$) δ 7.93 (d, *J* = 8.31 Hz, 2H), 7.39 (t, *J* = 3.77 Hz, 1H), 7.18 (d, *J* = 8.26 Hz,

2H), 3.91 (s, 1H), 3.88 (s, 3H), 2.40-2.52 (m, 2H), 1.95-1.89 (m,1H), 1.78-1.72 (m, 1H), 1.56-1.50 (m, 1H), 1.47-1.41 (m, 1H); ^{13} C NMR (500 MHz, $CDCl_3$) δ 171.82, 167.13, 150.30, 144.89, 130.66, 129.60, 128.05, 127.73, 51.95, 39.22, 31.03, 26.03, 16.64. FT-IR (neat, cm^{-1}): 3438.49, 2934.86, 1718.23, 1631.06, 1283.21, 1109.98, 706.92, 502.6.

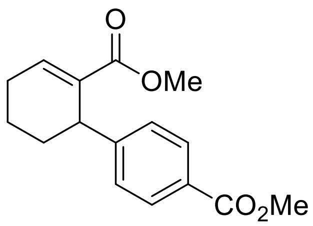

Dimethyl 1,4,5,6-tetrahydro-[1,1'-biphenyl]-2,4'-dicarboxylate (3c): This compound was prepared by the general procedure described above and was obtained as white solid (24 h, 70%). ESI *m/z* 297.1100 $[M+Na]^+$. R_f(PE: EtOAc = 10:1): 0.5. ^{1}H NMR (500 MHz, $CDCl_3$) δ 7.87 (d, J = 8.24 hz, 2H), 7.21 (t, *J* = 3.85 Hz, 1H), 7.14 (d, *J* = 8.25 Hz, 2H), 3.81 (s, 3H), 3.50 (s, 3H), 2.30-2.20 (m, 2H), 1.85-1.65 (m, 3H), 1.47-1.40 (m, 2H); ^{13} C NMR (500 MHz, $CDCl_3$) δ 167.27, 167.11, 150.66, 142.22, 131.32, 129.59, 128.01, 127.71, 51.94, 51.53, 39.75, 31.21, 25.83, 17.03. FT-IR (neat, cm^{-1}): 3414.74, 2946.03, 1713.83, 1438.62, 1279.00, 1107.87,766.99, 558.48.

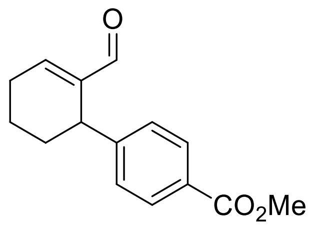

Methyl 6'-formyl-1',2',3',4'-tetrahydro-[1,1'-biphenyl]-4-carboxylate (3d): This compound was prepared by the general procedure described above and was obtained as white solid (24 h, 65%). R_f (PE: EtOAc = 10:1): 0.5. ^{1}H NMR (500 MHz, $CDCl_3$) δ 9.37 (s, 1H), 7.86 (d, *J* = 8.50 Hz, 2H), 7.09 (d, *J* = 8.26 Hz, 2H), 7.06 (d, *J* = 3.80 Hz, 1H), 3.86 (s,1H), 3.81 (s, 3H), 2.40-2.52 (m, 2H), 1.87-1.75 (m, 1H), 1.73-1.68 (m, 1H), 1.54-1.48 (m, 2H); ^{13} C NMR (500 MHz, $CDCl_3$) δ 193.25, 167.09, 153.46, 149.45, 142.71, 129.63, 128.10, 127.78, 37.45, 26.57, 17.57. FT-IR (neat, cm^{-1}): 3433.95, 3308.68, 1629.32, 1319.87,

1013.92, 1022.65, 703.32, 510.98.

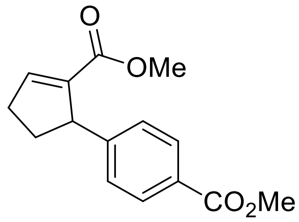

Methyl 4-(2-(methoxycarbonyl) cyclopent-1-en-1-yl) benzoate (3e): This compound was prepared by the general procedure described above and was obtained as white solid (24 h, 89%). R_f (PE: EtOAc = 10:1): 0.3. ^1H NMR (500 MHz, $CDCl_3$) δ 7.96 (d, J = 8.14 Hz, 2H), 7.14 (d, J = 8.20 Hz, 2H), 7.04 (d, J = 1.69 Hz, 1H), 4.21 (d, J = 6.00 Hz, 1H), 3.90 (s, 3H), 3.61 (s, 3H), 2.72-2.68 (m, 1H), 2.69-2.64 (m, 2H), 1.94-1.89 (m, 1H); ^{13}C NMR (500 MHz, $CDCl_3$) δ 167.07, 164.92, 150.63, 145.51, 138.67, 129.87, 128.22, 127.03, 51.94, 51.37, 50.15, 33.87, 32.29. FT-IR (neat, cm^{-1}): 2951.27, 2845.45, 1722.92, 1611.50, 1275.20, 1021.95, 767.69, 554.69.

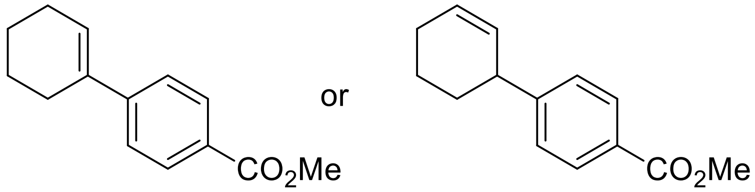

Methyl 4-(3-oxocyclohept-1-en-1-yl) benzoate (3f/3g): This compound was prepared by the general procedure described above and was obtained as a mixture of double bond isomers. (2.2:1, 20 mg, 75%). R_f (PE: EtOAc = 10:1): 0.3. ^1H NMR (500 MHz, $CDCl_3$) δ 7.91-7.88 (m, 4H), 7.24 (dd, J = 34.00 Hz, 4H), 5.69 (s, 2H), 3.83 (s, 6H), 2.31 (s, 2H), 2.20-2.05 (m, 8H), 1.72-1.55 (m, 6H); ^{13}C NMR (500 MHz, $CDCl_3$) δ 167.16, 152.73, 147.71, 135.91, 129.76, 129.67, 129.58, 129.24, 129.01, 128.05, 127.97, 127.76, 127.19, 127.05, 126.95, 126.39, 124.72, 51.96, 40.25, 33.02, 29.43, 27.18, 25.64, 22.91, 21.98, 21.06. FT-IR (neat, cm^{-1}): 2945.56, 2855.61, 1715.39, 1435.60, 1277.55, 1018.95, 853.60, 703.29.

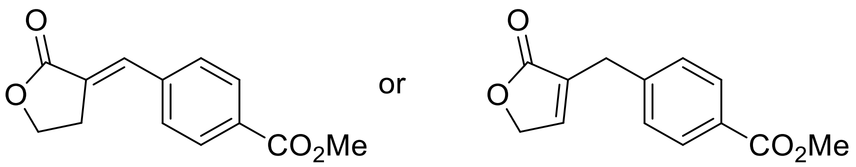

(E)-methyl 4-((2-oxodihydrofuran-3(2H)-ylidene) methyl) benzoate compound with methyl 4-((2-oxo-2,5-dihydrofuran-3-yl)methyl)benzoate (3h/3i): This compound was prepared by the general procedure described above and was obtained as a mixture of double bond isomers. (1:1， 24 h, 75%). R_f (PE: EtOAc = 10:1): 0.3. ^{1}H NMR (500 MHz, $CDCl_3$) δ 8.02 (d, *J* = 9.25 Hz, 2H), 7.92 (d, *J* = 6.51 Hz, 2H), 7.49 (d, *J* = 8.56 Hz, 2H), 7.23 (s, 2H), 4.71 (dd, *J* = 4.05 Hz, 3H), 4.42 (d, *J* = 7.22 Hz, 2H), 3.86 (d, *J* = 1.61 Hz, 3H), 3.83 (s, 3H), 3.59 (d, *J* = 1.60 Hz, 3H), 3.21 (d, *J* = 7.30 Hz, 2H); ^{13}C NMR (500 MHz, $CDCl_3$) δ 173.70, 172.00, 166.83, 166.35, 145.99, 142.64, 138.81, 135.24, 130.07, 130.04, 129.75, 128.97, 70.34, 65.48, 65.38, 52.37, 52.13, 31.80, 27.50. FT-IR (neat, cm^{-1}): 1752.69, 1745.60, 1715.56, 1275.60, 1260.43, 1156.97, 596.03.

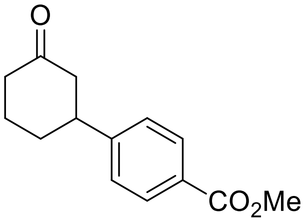

Methyl 5'-oxo-2',3',4',5'-tetrahydro-[1,1'-biphenyl]-4-carboxylate (3j): This compound was prepared by the general procedure described above and was obtained as white solid (24 h, 60%). R_f (PE: EtOAc = 10:1): 0.4. ^{1}H NMR (500 MHz, $CDCl_3$) δ 7.92 (d, *J* = 7.74 Hz, 2H), 7.22 (d, *J* = 8.04 Hz, 2H), 3.83 (d, *J* = 0.86 Hz, 3H), 2.99 (dd, *J* = 11.52 Hz, 1H), 2.51-2.39 (m, 4H), 2.10-1.95 (m, 2H), 1.82-1.70 (m, 2H); ^{13}C NMR (500 MHz, $CDCl_3$) δ 210.30, 166.85, 149.44, 130.07, 128.70, 126.66, 52.07, 48.47, 44.66, 41.11, 32.47, 25.43. FT-IR (neat, cm^{-1}): 2937.65, 2871.64, 1715.58, 1314.63, 1182.62, 1018.11, 702.73, 541.72.

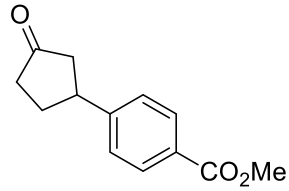

Methyl 4-(2-oxocylopentyl) benzoate (3k): This compound was prepared by the general procedure described above and was obtained as white solid (24 h, 75%). R_f (PE: EtOAc = 10:1): 0.3. ^1H NMR (500 MHz, $CDCl_3$) δ 7.91 (d, J = 8.29 Hz, 2H), 7.22 (d, J = 8.31 Hz, 2H), 3.81 (s, 3H), 3.42-3.35 (m, 1H), 2.65-2.63 (m, 1H), 2.40-2.35 (m, 2H), 2.27-2.20 (m, 2H), 1.95-1.87 (m, 1H) ; ^{13}C NMR (500 MHz, $CDCl_3$) δ 217.54, 166.83, 148.36, 130.04, 128.75, 126.81, 52.11, 45.47, 42.22, 38.74, 30.98. FT-IR (neat, cm^{-1}): 2976.42, 1741.68, 1605.66, 1281.10, 1013.60, 859.15, 614.06, 421.92.

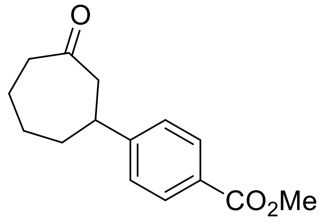

Methyl 4-(3-oxocyclohept-1-en-1-yl) benzoate (3l): This compound was prepared by the general procedure described above and was obtained as white solid (24 h, 75%). R_f (PE: EtOAc = 10:1): 0.3. ^1H NMR (500 MHz, $CDCl_3$) δ 7.90 (d, J = 8.29 Hz, 2H), 7.18 (d, J = 8.28 Hz, 2H), 3.83 (s, 3H), 2.90-2.85 (m, 2H), 2.57-2.50 (m, 3H), 2.03-1.95 (m, 3H), 1.72-1.65 (m, 3H); ^{13}C NMR (500 MHz, $CDCl_3$) δ 212.85, 166.91, 152.05, 130.08, 128.40, 126.50, 52.05, 50.77, 43.91, 42.76, 38.91, 29.19, 24.09. FT-IR (neat, cm^{-1}): 3439.54, 3385.40, 1763.76, 1716.63, 1442.43, 128.20, 825.20, 509.83.

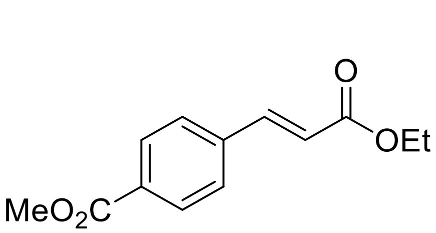

(E)-methyl 4-(3-ethoxy-3-oxoprop-1-en-1-yl) benzoate (3m): This compound was prepared by the general procedure described above and was obtained as white solid (24 h,

93%). R_f (PE: EtOAc = 10:1): 0.3. ^{1}H NMR (500 MHz, $CDCl_3$) δ 7.97 (d, *J* = 8.12Hz, 2H), 7.62 (d, *J* = 16.04 Hz, 1H), 7.50 (d, *J* = 8.36 Hz, 2H), 6.44 (d, *J* = 16.04 Hz, 1H), 4.20 (q, *J* = 7.11 Hz, 2H), 3.85 (d, *J* = 0.53 Hz, 3H), 1.27 (q, *J* = 6.98Hz, 3H); ^{13}C NMR (500 MHz, $CDCl_3$) δ 166.52, 166.45, 143.14, 138.68, 131.32, 130.09, 127.88, 120.67, 60.74, 52.28, 14.29. FT-IR (neat, cm^{-1}): 3408.45, 2968.38, 1714.56, 1636.65, 1273.42, 1412.77, 1172.60, 772.69, 462.35.

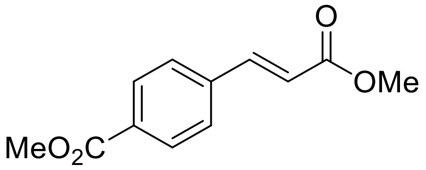

(E)-methyl 4-(3-methoxy-3-oxoprop-1-en-1-yl) benzoate (3n): This compound was prepared by the general procedure described above and was obtained as white solid (24 h, 96%). R_f (PE : EtOAc = 10:1): 0.3 .^{1}H NMR (500 MHz, $CDCl_3$) δ 7.97 (d, *J* = 7.86 Hz, 2H), 7.62 (d, *J* = 16.05Hz, 1H), 7.50 (d, *J* = 8.06Hz, 2H), 6.44 (d, *J* = 16.05 Hz, 1H), 3.85 (d, *J* = 0.86Hz, 3H), 3.74 (d, *J* = 0.82Hz, 3H); ^{13}C NMR (500 MHz, $CDCl_3$) δ 166.96, 166.42, 143.44, 138.57, 131.39, 130.10, 127.91, 120.17, 52.29, 51.89. FT-IR (neat, cm^{-1}): 3017.28, 2955.46, 2363.82, 1720.47, 1279.00, 1105.42, 722.93, 499.60.

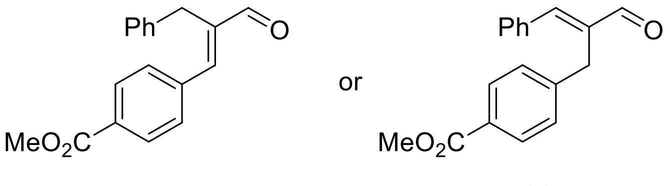

(Z)-methyl 4-(2-formyl-3-phenylallyl) benzoate (3o/3p): This compound was prepared by the general procedure described above and was obtained as a mixture of double bond isomers. (10:1, 24 h, 73%). R_f (PE: EtOAc = 10:1): 0.4. ESI *m/z* 303.2990 $[M+Na]^+$. ^{1}H NMR (500 MHz, $CDCl_3$) δ 9.73 (s, 2H), 7.96 (d, *J* = 8.34Hz, 4H), 7.57 (s, 2H), 7.46 (d, *J* = 7.00 Hz, 4H), 7.42-7.40 (m, 6H), 7.24 (d, *J* = 8.50 Hz, 4H), 4.01 (s, 4H), 3.91 (s, 6H); ^{13}C NMR (500 MHz, $CDCl_3$) δ 194.92, 194.78, 166.99, 152.04, 143.91, 139.73, 134.28, 130.17, 129.97, 129.69, 128.93, 128.30, 128.01, 52.02, 30.56. FT-IR (neat, cm^{-1}): 2646.76, 3035.44, 1720.48, 1612.55, 1281.20, 1109.27, 757.65, 590.96.

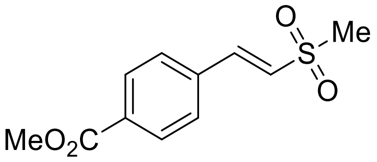

(E)-methyl 4-(2-(methylsulfonyl) vinyl) benzoate (3q): This compound was prepared by the general procedure described above and was obtained as white solid (24 h, 89%). R_f (PE: EtOAc = 3:1): 0.3. ^{1}H NMR (500 MHz, $CDCl_3$) δ 8.09 (d, *J* = 8.19 Hz, 2H), 7.63 (dd, *J* = 26.62 Hz, 3H), 7.04 (d, *J* = 15.04Hz, 1H), 3.95 (s, 3H), 3.07 (s, 3H); ^{13}C NMR (500 MHz, $CDCl_3$) δ 166.13, 142.52, 136.21, 132.43, 130.30, 128.63, 128.46, 52.40, 43.16. FT-IR (neat, cm^{-1}): 3056.05, 3006.01, 1716.29, 1435.16, 1268.75, 1113.80, 856.07, 760.00, 451.28.

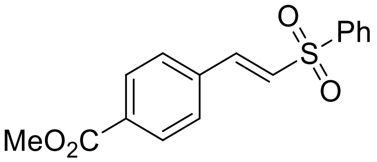

(E)-methyl 4-(2-(phenylsulfonyl) vinyl) benzoate (3r): This compound was prepared by the general procedure described above and was obtained as white solid (24 h, 73%). R_f (PE: EtOAc = 3:1): 0.4. ^{1}H NMR (500 MHz, $CDCl_3$) δ 7.98-7.85 (m, 4H), 7.65-7.56 (m, 2H), 7.50-7.45 (m, 4H), 6.88 (dd, *J* = 16.00 Hz, 1H), 3.83 (d, *J* = 4.89 Hz, 3H); ^{13}C NMR (500 MHz, $CDCl_3$) δ 166.14, 140.89, 136.51, 133.64, 132.23, 130.22, 129.79, 129.44, 128.44, 127.80, 52.37. FT-IR (neat, cm^{-1}): 3420.68, 3050.56, 1717.68, 1438.97, 1276.21, 1080.98, 979.69, 557.83.

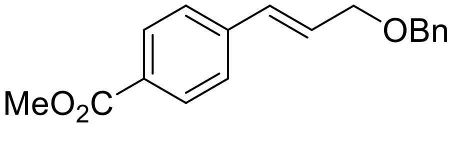

(E)-methyl 4-(3-(benzyloxy) prop-1-en-1-yl) benzoate (3s): This compound was prepared by the general procedure described above and was obtained as colorless oil (24 h, 71%). R_f (PE: EtOAc = 10:1): 0.3. ^{1}H NMR (500 MHz, $CDCl_3$) δ 8.01 (d, *J* = 8.90 Hz, 2H), 7.46(d, *J*=7.37Hz, 2H), 7.41-7.30 (m, 5H), 6.70 (d, *J* = 16.00Hz, 1H), 6.50-6.43 (m, 1H), 4.61 (d, *J* = 6.99 Hz, 2H), 4.25 (dd, *J* = 6.00 Hz, 2H), 3.93 (d, *J* = 2.66 Hz, 3H); ^{13}C NMR (500 MHz,

$CDCl_3$) δ 166.89, 141.26, 131.12,129.94, 129.10, 128.98, 128.48, 127.81, 127.76, 126.35, 72.49, 40.46, 52.08. FT-IR (neat, cm^{-1}): 3457.00, 2923.33, 1718.35, 1275.86, 1173.61, 1030.68, 741.50, 455.45.

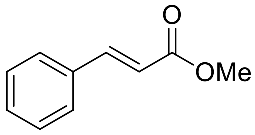

Methyl cinnamate (4a): This compound was prepared by the general procedure described above and was obtained as white solid (24 h, 75%). R_f (PE : EtOAc = 10:1): 0.3 . ^{1}H NMR (500 MHz, $CDCl_3$) δ 7.62（d, *J* = 16.03 Hz, 1H）, 7.44-7.46 (m, 3H), 6.36 (d, *J* = 16.03 Hz,1H), 3.73 (s,3H); ^{13}C NMR (500 MHz, $CDCl_3$) δ 167.43, 144.88, 134.41, 130.30, 128.90, 128.08, 117.83, 51.69. FT-IR (neat, cm^{-1}): 2929.27, 1716.28, 1634.55, 1490.66, 1313.59, 1279.36, 982.13, 717.39, 687.06.

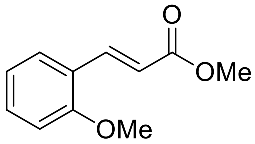

(E)-methyl 3-(2-methoxyphenyl) acrylate (4b): This compound was prepared by the general procedure described above and was obtained as colorless oil (24 h, 80%). R_f (PE: EtOAc = 10:1): 0.3. ^{1}H NMR (500 MHz, $CDCl_3$) δ 7.83 (d, *J* = 16.18 Hz, 1H), 7.43 (dd, *J* = 7.70 Hz, 1.50 Hz, 1H), 7.29–7.25 (m, 1H), 6.90– 6.88 (m, 2H), 6.46 (d, *J* = 16.20 Hz, 1H), 3.81 (s, 3H), 3.73 (s, 3H); ^{13}C NMR (500 MHz, $CDCl_3$) δ 167.92, 158.36, 140.27, 131.49, 128.91, 123.39, 120.70, 118.33, 111.16, 55.46, 51.56. FT-IR (neat, cm^{-1}): 2949.62, 1709.53, 1630.06, 1319.62, 1245.29, 1160.57, 1023.52, 986.02, 749.39.

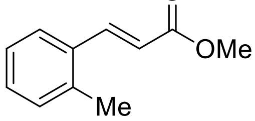

(E)-methyl 3-(o-tolyl) acrylate (4c): This compound was prepared by the general procedure described above and was obtained as colorless oil (24 h, 94%). R_f (PE: EtOAc = 10:1): 0.3.

^{1}H NMR (500 MHz, $CDCl_3$) δ 7.9 (d, J = 15.91 Hz, 1H), 7.48-7.43 (m, 1 H), 7.15-7.20 (m, 1H), 7.12 (t, J = 6.99 Hz, 2H), 6.28 (d, J = 15.90 Hz ,1H), 3.73 (s, 3H), 2.35 (s, 3H); ^{13}C NMR (500 MHz, $CDCl_3$) δ167.48, 142.56, 137.67, 133.39, 130.80, 130.04, 126.42, 126.35, 118.88, 51.68, 19.79. FT-IR (neat, cm^{-1}): 2950.19, 1712.80, 1632.59, 1434.09, 1314.66, 1269.85, 978.35, 783.45.

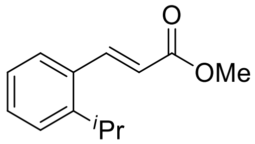

(E)-methyl 3-(2-isopropylphenyl) acrylate (4d): This compound was prepared by the general procedure described above and was obtained as colorless oil (24 h, 86%). R_f (PE: EtOAc = 10:1): 0.3. ^{1}H NMR (500 MHz, $CDCl_3$) δ 8.05（d, J = 15.77 Hz, 1H）, 7.35-7.24 (m, 2H), 7.15-7.10 (m, 1H), 6.26 (d, J = 15.76 Hz ,1H), 3.73 (s, 3H), 3.26 (dt, J = 7.56 Hz , 2H), 1.17 (d, J = 6.90 Hz, 6H); ^{13}C NMR (500 MHz, $CDCl_3$) δ167.46, 147.91, 142.70, 132.47, 130.27, 126.77, 126.08, 125.5, 119.55, 51.68, 29.2, 23.63; FT-IR (neat, cm^{-1}): 2961.40, 1719.08, 1477.04, 1271.65, 1170.73, 1034.56, 833.35, 527.05.

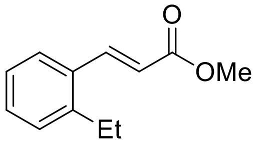

(E)-methyl 3-(2-ethylphenyl) acrylate (4e): This compound was prepared by the general procedure described above and was obtained as colorless oil (24 h, 86%). R_f (PE: EtOAc = 10:1): 0.3. ^{1}H NMR (500 MHz, $CDCl_3$) δ 7.95 (d, J = 15.83 Hz, 1H）, 7.47 (d, J = 7.69 Hz, 1 H), 7.23 (t, J = 7.43 Hz, 1H), 7.16-7.10 (m, 2H), 6.29 (d, J = 15.83 Hz, 1H), 3.73 (d, J = 0.83 Hz, 3H), 2.7 (t, J = 7.56 Hz , 2H),1.14 (t, J = 0.58 Hz 3H); ^{13}C NMR (500 MHz, $CDCl_3$) δ 167.49, 143.85, 142.38, 132.71, 130.23, 129.24, 126.55, 126.35, 119.03, 51.68, 26.38, 15.85. FT-IR (neat, cm^{-1}): 2922.98, 2851.73, 1720.48,1633.86, 1272.72, 1170.39, 980.04, 763.65.

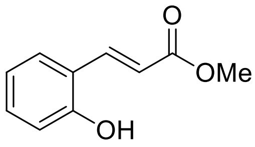

(E)-methyl 3-(2-hydroxyphenyl) acrylate (4f): White solid(24 h, 62%). [1] H NMR (400 MHz, $CDCl_3$) δ 7.97 (d, *J* = 16.16 Hz, 1H), 7.47 (d, *J* = 7.95 Hz, 1H), 7.22−7.18 (m, 1H), 6.84 (d, *J* = 7.99 Hz, 2H), 6.59 (d, *J* = 10.15 Hz, 1H), 4.85(s, 1H), 3.77 (s, 3H). ^{13}C NMR (400 MHz, $CDCl_3$) δ 168.62, 156.89, 140.97, 131.26, 128.66, 119.38, 116.61, 115.63, 50.62. FT-IR (neat, cm^{-1}): 3433.65, 2845.08, 1691.83, 1455.64, 1172.83, 1015.32, 864.78, 458.60.

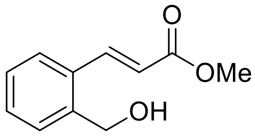

(E)-methyl 3-(2- (hydroxymethyl) phenyl) acrylate (4g): This compound was prepared by the general procedure described above and was obtained as colorless oil (24 h, 65%). R_f (PE: EtOAc = 10:1): 0.3. [1] H NMR (500 MHz, $CDCl_3$) δ 8.04 (d, *J* = 15.88 Hz, 1H), 7.59 (d, *J* = 7.58 Hz, 1H), 7.45-7.30 (m, 4H), 6.43-6.38 (m, 1H), 4.80 (d, *J* = 2.00 Hz, 2H), 3.79 (d, *J* = 4.88 Hz, 3H); [13] C NMR (500 MHz, $CDCl_3$) 167.55, 141.80, 139.67, 133.01, 130.23, 128.80, 128.22, 126.78, 119.59, 62.79, 51.84. FT-IR (neat, cm^{-1}): 3412.99, 3138.45, 2948.13, 1712.71, 1632.10, 1432.56, 1011.48, 766.64.

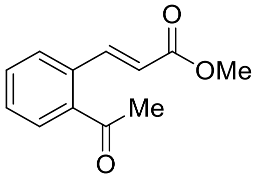

(E)-methyl 3-(2-acetylphenyl) acrylate (4h): This compound was prepared by the general procedure described above and was obtained as colorless oil (24 h, 90%). R_f (PE: EtOAc = 10:1): 0.3. [1] H NMR (500 MHz, $CDCl_3$) δ 8.16 (d, *J* = 15.90 Hz, 1H), 7.76 (d, *J* = 7.80 Hz, 1H), 7.59 (d, *J* = 7.70 Hz, 1H), 7.52 (t, *J* = 7.10 Hz, 1H), 7.47 (t, *J* = 7.00 Hz, 1H), 6.29 (d, *J* = 16.00 Hz, 1H), 3.81 (s, 3H), 2.62 (s, 3H); [13] C NMR (500 MHz, $CDCl_3$) δ 200.8, 166.94, 144.20, 138.21, 134.80, 131.99, 129.44, 129.25, 128.38, 120.52, 51.74, 29.24; FT-IR (neat,

cm^{-1}): 2988.42, 1718.33, 1637.56, 1437.43, 1254.31, 970.96,767.69, 502.14.

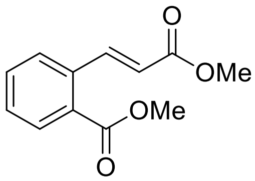

(E)-methyl 2-(3-methoxy-3-oxoprop-1-en-1-yl) benzoate (4i): This compound was prepared by the general procedure described above and was obtained as colorless oil (24 h, 89%). R_f (PE: EtOAc = 10:1): 0.3. ^{1}H NMR (500 MHz, $CDCl_3$) δ 8.37 (d, J = 15.94 Hz, 1H), 7.89 (d, J = 7.80 Hz, 1H), 7.52-7.44 (m, 2H), 7.36 (t, J = 7.20 Hz, 1H), 6.23 (t, J = 7.80 Hz, 1H), 3.85 (s, 3H), 3.74 (s, 3H); ^{13}C NMR (500 MHz, $CDCl_3$) δ167.71, 167.01, 143.94, 136.37, 132.39, 130.78, 129.77, 129.41, 127.92, 120.69, 52.42, 51.80. FT-IR (neat, cm^{-1}): 2969.30, 1720.43, 1635.95, 1437.22, 1130.57, 1078.52, 977.25, 724.03.

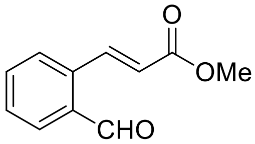

(E)-methyl 3-(2-formylphenyl) acrylate (4j): This compound was prepared by the general procedure described above and was obtained as white solid (24 h, 86%). R_f (PE: EtOAc = 10:1): 0.3.^{1}H NMR (500 MHz, $CDCl_3$) δ 10.28 (s, 1H), 8.53 (d, J = 15.50 Hz, 1H), 7.88 (d, J = 7.41 Hz, 1H), 7.64-7.56 (m, 3H), 6.38 (d, J = 16.00 Hz, 1H), 3.83 (s, 3H); ^{13}C NMR (500 MHz, $CDCl_3$) δ 191.85, 166.66, 141.29, 136.50, 133.91, 133.84, 132.44, 129.94, 127.97, 122.70, 51.91. FT-IR (neat, cm^{-1}): 33059.54, 2952.39, 1712.43, 1631.41, 1437.22, 1280.40, 765.59, 425.06.

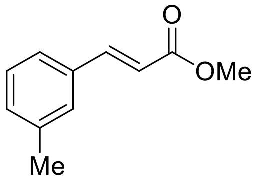

(E)-methyl 3-(m-tolyl) acrylate (4k): This compound was prepared by the general procedure described above and was obtained as colorless oil (24 h, 60%). R_f (PE: EtOAc =

10:1): 0.3. [1] H NMR (500 MHz, $CDCl_3$) δ 7.59 (d, J = 17.02 Hz, 1H), 7.25 (m, J = 6.17 Hz, 2H), 7.10-7.22 (m, 1H), 7.12 (d, J = 7.46, 1H), 7.35 (d, J = 16.02, 1H), 3.37 (s, 3H), 2.29 (s, 3H);[13] C NMR (500 MHz, $CDCl_3$) δ167.52, 145.07, 138.56, 134.36, 131.14, 128.78, 128.74, 125.27, 117.58, 51.67, 21.32. FT-IR (neat, cm^{-1}): 3410.55, 3022.85, 2079.01, 1716.91, 1317.09, 1169.08, 1045.72, 905.66.

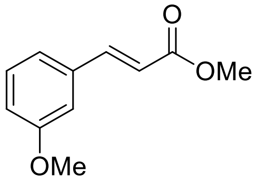

(E)-methyl 3-(3-methoxyphenyl) acrylate (4l): This compound was prepared by the general procedure described above and was obtained as colorless oil (24 h, 70%). R_f (PE: EtOAc = 10:1): 0.3. [1] H NMR (500 MHz, $CDCl_3$) δ 7.58（d, J = 16.00 Hz, 1H）,7.25-7.18 (m, 1H), 7.04 (d, J = 7.65 Hz, 1H), 6.96 (d, J=7.60 Hz, 1H), 6.85(dd, J=8.20Hz, 1H), 6.35(d, J=16.00Hz, 1H), 3.75(s, 3H), 3.73(s, 3H); [13] C NMR (500 MHz, $CDCl_3$) δ 167.37, 159.91, 144.79, 135.76, 129.89, 120.76, 118.11, 116.14, 112.99, 55.29, 51.71. FT-IR (neat, cm^{-1}): 2949.87, 2640.56, 1716.68, 1583.51, 1488.52, 1255.69, 854.03, 583.17.

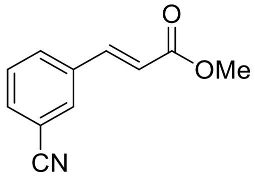

(E)-methyl 3-(3-cyanophenyl) acrylate (4m): This compound was prepared by the general procedure described above and was obtained as white solid (24 h, 75%). R_f (PE: EtOAc = 10:1): 0.3. [1] H NMR (500 MHz, $CDCl_3$) δ 7.71 (d, J = 1.53 Hz, 1H), 7.67 (d, J = 8.00 Hz, 1H), 7.6-7.55 (m, 2H), 7.45 (d, J = 16.10 Hz, 1H), 6.42 (d, J = 16.06 Hz, 1H), 3.75 (s, 3H); ^{13}C ($CDCl_3$) δ 166.61, 142.06, 135.66, 133.21, 131.90, 131.33, 129.83, 120.56, 118.13, 113.39, 51.98. FT-IR (neat, cm^{-1}): 3409.85, 2920.89, 2225.16, 1721.52, 1640.16, 1226.06, 981.09, 465.58.

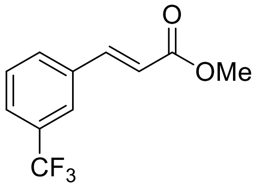

(E)-methyl 3-(3-(trifluoromethyl) phenyl) acrylate (4n): This compound was prepared by the general procedure described above and was obtained as white solid (24 h, 80%). R_f(PE: EtOAc = 10:1): 0.3. ^{1}H NMR (500 MHz, $CDCl_3$) δ 7.68 (s, 1H), 7.62 (m, *J* = 6.17 Hz,2H), 7.55 (m, 1H), 7.43 (d, *J* = 7.46 Hz, 1H), 6.42 (d, *J* = 16.05 Hz, 1H), 3.74 (s, 3H);^{13}C NMR (500 MHz, $CDCl_3$) δ 166.86, 143.01, 135.19, 131.04, 131.03, 129.45, 126.67, 126.63, 124.62, 51.67, 21.32. FT-IR (neat, cm^{-1}): 2956.12, 1711.74, 1642.69, 1444.29, 1124.89, 1075.04, 882.68, 509.94.

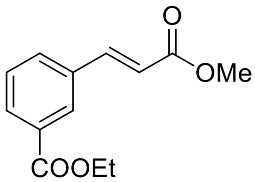

(E)-ethyl 3-(3-methoxy-3-oxoprop-1-en-1-yl) benzoate (4o): This compound was prepared by the general procedure described above and was obtained as white solid (24 h, 95%). R_f(PE: EtOAc = 10:1): 0.3. ^{1}H NMR (500 MHz, $CDCl_3$) δ 8.21(d, *J* = 16.01 Hz, 1H), 8.06 (d, *J* = 14.00 Hz, 1H), 7.75-7.68 (m, 2H), 7.46(dd, *J* = 15.00 Hz, 1H), 6.57-6.48 (m, 1H), 4.45-4.48 (m, 2H), 3.85-3.80 (m, 3H), 1.45-1.38 (m, 3H); ^{13}C NMR (500 MHz, $CDCl_3$) δ 167.08, 165.95, 143.69, 134.67, 132.07, 131.29, 131.07, 128.96, 119.07, 61.25, 51.77, 14.30. FT-IR (neat, cm^{-1}): 2920, 1703, 1631, 1434, 1316, 1166, 998, 814, 509, 491.

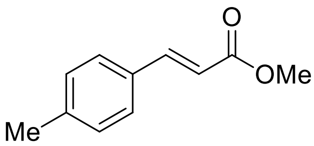

(E)-methyl 3-(p-tolyl) acrylate (4p): This compound was prepared by the general procedure described above and was obtained as white solid (24 h, 80%). R_f (PE: EtOAc = 10:1): 0.3. ^{1}H NMR (500 MHz, $CDCl_3$) δ 7.59 (d, *J* = 16.01 Hz, 1H),7.34(d, *J* = 8.10 Hz, 2H), 7.11 (d, *J* = 7.99 Hz, 2H),6.31 (d, *J* = 16.00 Hz, 1H), 3.72 (s, 3H), 2.99(s, 3H); ^{13}C NMR (500 MHz,

CDCl3) δ167.65, 144.90, 140.74, 131.66, 129.63, 128.09, 116.69, 51.65, 21.47; FT-IR (neat, cm^{-1}): 2918.44, 1707.23, 1602.43, 1317.78, 1168.69, 1000.30, 817.29, 513.78.

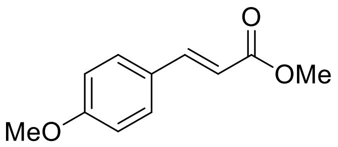

(E)-methyl 3-(4-methoxyphenyl) acrylate (4q): This compound was prepared by the general procedure described above and was obtained as white solid (24 h, 93%). R_f (PE: EtOAc = 3:1): 0.5; ^{1}H NMR (500 MHz, $CDCl_3$) δ 7.57 (d, *J* = 15.97 Hz, 1H), 7.39 (d , *J* = 7.00 Hz, 2 H), 6.82 (d, *J* = 7.00 Hz, 2 H), 6.23 (d, *J* = 15.96 Hz, 1H), 3.75 (s, 3H) , 3.71 (s, 3H); ^{13}C NMR (500 MHz, $CDCl_3$) δ167.76, 161.41, 144.53, 129.72, 127.14, 115.29, 114.34, 55.36, 51.56. FT-IR (neat, cm^{-1}): 2946.73, 1715.93, 1639.10, 1513.02, 1290.54, 1192.13, 1020.35, 983.50, 553.94.

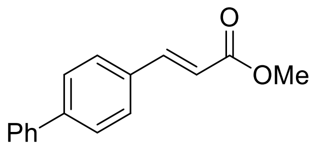

(E)-methyl 3-([1,1'-biphenyl]-4-yl) acrylate (4r): This compound was prepared by the general procedure described above and was obtained as white solid (24 h, 78%). R_f (PE: EtOAc = 3:1): 0.5. ^{1}H NMR (500 MHz, $CDCl_3$) δ 7.65 (d, *J* = 16.02 Hz, 1H), 7.53-7.49 (m, 6H), 7.38-7.34 (m, 2H), 7.28 (d, *J* = 6.80 Hz, 1H), 3.73 (s, 3H); ^{13}C NMR (500 MHz, $CDCl_3$) δ 167.48, 144.42, 143.09, 140.16, 133.37, 128.93, 128.60, 127.87, 127.56, 127.06, 117.67, 51.73. FT-IR (neat, cm^{-1}): 3423.47, 3379.49, 1715.24, 1192.04, 1169.06, 980.04, 694.35, 470.65.

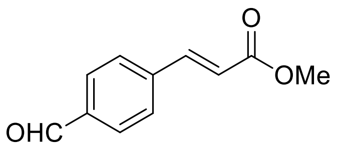

(E)-methyl 3-(4-formylphenyl) acrylate (4s): This compound was prepared by the general procedure described above and was obtained as white solid (24 h, 75%). R_f (PE: EtOAc =

3:1): 0.5. ^{1}H NMR (500 MHz, $CDCl_3$) δ 10.02 (s, 1H), 7.89 (d, *J* = 7.78 Hz, 2H), 7.66 (d, *J* = 7.86 Hz, 3H), 6.54 (d, *J* = 16.30 Hz, 1H), 3.82 (s,3H); ^{13}C NMR (500 MHz, $CDCl_3$) δ 191.35, 166.73, 143.07, 140.02, 137.22, 130.13, 129.62, 128.49, 128.27, 120.99, 51.89. FT-IR (neat, cm^{-1}): 3412.98, 2917.39, 1692.06, 1590.28, 1319.7, 1166.05, 923.40, 715.30, 494.57.

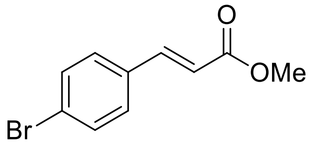

(E)-methyl 3-(4-bromophenyl) acrylate (4t): This compound was prepared by the general procedure described above and was obtained as white solid (24 h, 95%). R_f (PE: EtOAc = 3:1): 0.5. ^{1}H NMR (500 MHz, $CDCl_3$) δ 7.54 (d, *J* = 16.03 Hz, 1H), 7.44 (d , *J* = 8.50 Hz, 2 H), 7.30 (d, *J* = 8.50 Hz, 2 H), 6.34(d, *J* = 16.02 Hz, 1H), 3.73 (s, 3H); ^{13}C NMR (500 MHz, $CDCl_3$) δ167.15, 143.48, 133.31, 132.15, 129.44, 124.55, 118.52, 51.79. FT-IR (neat, cm^{-1}): 2921.34, 1706.16, 1485.07, 1315.68, 1163.05, 1066.66, 689.60, 465.85.

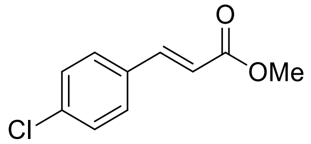

(E)-methyl 3-(4-chlorophenyl) acrylate (4u): This compound was prepared by the general procedure described above and was obtained as white solid (24 h, 93%). R_f (PE: EtOAc = 3:1): 0.5. ^{1}H NMR (500 MHz, $CDCl_3$) δ 7.55 (d, *J* = 16.03 Hz, 1H), 7.36 (d , *J* = 6.50 Hz, 2 H), 7.27 (d, *J* = 6.50 Hz, 2 H), 6.32 (d, *J* = 16.02 Hz, 1H), 3.72 (s, 3H); ^{13}C NMR (500 MHz, $CDCl_3$) δ167.15, 143.39, 136.21, 132.88, 129.22, 129.17, 118.40, 51.77. FT-IR (neat, cm^{-1}): 2947.43, 1705.81, 1588.80, 1432.30, 1197.98, 1080.63, 1006.93, 827.06, 503.65.

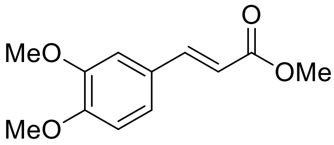

(E)-methyl 3-(3,4-dimethoxyphenyl) acrylate (4v): This compound was prepared by the

general procedure described above and was obtained as colorless oil (24 h, 90%). R_f (PE: EtOAc = 3:1): 0.5. ^1H NMR (500 MHz, $CDCl_3$) δ 7.64 (d, *J* = 15.94 Hz, 1H), 7.12-7.05 (m, 2H), 6.85 (d, *J* = 2.00 Hz, 1H), 6.31 (d, *J* = 15.93 Hz, 1H), 3.91 (s, 6H), 3.80 (s, 3H); ^{13}C NMR (500 MHz, $CDCl_3$) δ 167.64, 151.14, 149.21, 144.77, 127.36, 122.58, 115.48, 111.05, 109.66, 55.95, 55.87, 51.59. FT-IR (neat, cm^{-1}): 3436.39, 2921.24, 1695.33, 1591.95, 1250.36, 1021.25, 810.69, 411.44.

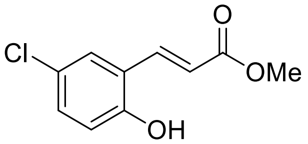

(E)-methyl 3-(5-chloro-2-hydroxyphenyl) acrylate (4w): This compound was prepared by the general procedure described above and was obtained as white solid (24 h, 70%). R_f (PE: EtOAc = 3:1): 0.5. ^1H NMR (500 MHz, $CDCl_3$) δ 7.87 (d, *J* = 16.17 Hz, 1H), 7.47 (s, 1H), 7.17 (d, *J* = 8.50 Hz, 1H), 6.82 (d, *J* = 8.72 Hz, 1H), 6.59 (d, *J* = 16.20 Hz, 1H), 4.90 (s, 1H), 3.77 (s, 3H); ^{13}C NMR (500 MHz, $CDCl_3$) δ 169.13, 155.55, 139.34, 130.68, 127.80, 124.06, 122.72, 118.02, 117.02, 50.75. FT-IR (neat, cm^{-1}): 3397.62, 1699.51, 1624.43, 1427.45, 1229.06, 1330.00, 1176.33, 415.98.

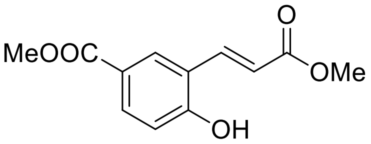

(E)-methyl 4-hydroxy-3-(3-methoxy-3-oxoprop-1-en-1-yl) benzoate (4x): This compound was prepared by the general procedure described above and was obtained as white solid (24 h, 69%). R_f (PE: EtOAc = 3:1): 0.5. ^1H NMR (500 MHz, $CDCl_3$) δ 8.12 (d, *J* = 2.14 Hz, 1H), 7.90 (d, *J* = 16.19 Hz, 1H), 7.86-7.84 (m,1H), 6.89 (d, *J* = 8.59 Hz, 1H), 6.63 (d, *J*=16.18 Hz, 1H), 4.89 (s, 1H), 3.87 (s, 3H), 3.89 (s, 3H); ^{13}C NMR (500 MHz, $CDCl_3$) δ 168.13, 166.68, 160.99, 139.71, 132.43, 130.69, 121.28, 121.17, 118.07, 115.50, 51.06, 50.75. FT-IR (neat, cm^{-1}): 3393.44, 2946.38, 1722.92, 1601.38, 1177.72, 992.81, 757.56, 484.79.

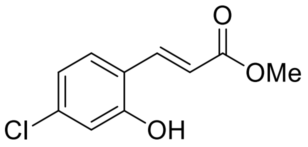

(E)-methyl 3-(4-chloro-2-hydroxyphenyl) acrylate (4y): This compound was prepared by the general procedure described above and was obtained as white solid (24 h, 80%). R_f (PE: EtOAc = 3:1): 0.5. ^1H NMR (500 MHz, $CDCl_3$) δ 7.87 (d, *J* = 16.18 Hz, 1H), 7.48 (d, *J* = 2.57 Hz, 1H), 7.18 (dd, *J* = 8.20 Hz, 1H), 6.82 (d, *J* = 8.73 Hz, 1H), 6.59 (d, *J* = 16.17 Hz, 1H), 4.89 (s, 1H), 3.77 (s, 3H); ^{13}C NMR (500 MHz, $CDCl_3$) δ 169.13, 155.55, 139.34, 130.68, 127.80, 124.07, 122.72, 118.02, 117.02, 50.75. FT-IR (neat, cm^{-1}): 3396.33, 2919.50, 1698.82, 1492.06, 1330.00, 1175.63, 854.31, 464.18.

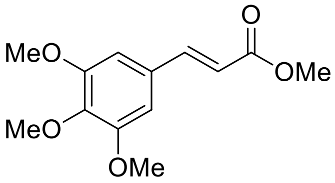

(E)-methyl 3-(3,4,5-trimethoxyphenyl) acrylate (4z): This compound was prepared by the general procedure described above and was obtained as white solid (24 h, 75%). R_f (PE: EtOAc = 3:1): 0.5. ^1H NMR (500 MHz, $CDCl_3$) δ 7.53 (d, *J* = 15.91 Hz, 1H), 6.68 (s, 2H), 6.27 (d, *J* = 15.91 Hz, 1H), 3.81 (d, *J* = 3.80 Hz, 9H), 3.73 (d, *J* = 0.71 Hz, 3H). ^{13}C NMR (500 MHz, $CDCl_3$) δ 167.38, 153.42, 144.85, 140.09, 129.87, 117.02, 105.19, 60.95, 56.13, 51.70. FT-IR (neat, cm^{-1}): 2947.43, 2837.06, 1697.06, 1561.12, 1336.98, 1126.72, 981.09, 835.60, 629.73.

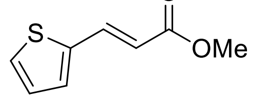

(E)-methyl 3-(thiophen-2-yl) acrylate (4ab): This compound was prepared by the general procedure described above and was obtained as white solid (24 h, 80%). R_f (PE: EtOAc = 3:1): 0.5. ^1H NMR (500 MHz, $CDCl_3$) δ 7.72 (d, *J* = 15.71 Hz, 1H), 7.30 (d , *J* = 5.01 Hz, 1 H), 7.18 (d, *J* = 3.58 Hz, 1 H), 6.97 (t, *J* = 4.40 Hz, 1H), 6.17 (d, *J* = 15.71 Hz, 1 H) 3.71(s, 3H); ^{13}C NMR (500 MHz, $CDCl_3$) δ 167.31, 139.51, 137.31, 130.96, 128.47, 128.09, 116.54,

51.73. FT-IR (neat, cm^{-1}): 2942.54, 1704.56, 1623.03, 1425.70, 1123.75, 983.18, 831.95, 483.70.

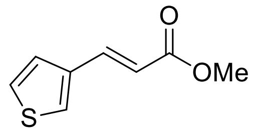

(E)-methyl 3-(thiophen-3yl) acrylate (4ac): This compound was prepared by the general procedure described above and was obtained as white solid (24 h, 85%). R_f (PE: EtOAc = 3:1): 0.5. ^{1}H NMR (500 MHz, $CDCl_3$) δ 7.60 (d, *J*=15.92 Hz, 1H), 7.41 (d , *J* = 2.24 Hz, 1 H), 7.26-7.18 (m, 2 H), 6.18 (d, *J* = 15.91 Hz, 1H), 3.71 (s, 3H); ^{13}C NMR (500 MHz, $CDCl_3$) δ 167.64, 138.31, 137.55, 128.07, 126.96, 125.17, 117.49, 51.62. FT-IR (neat, cm^{-1}): 3422.78, 3093.05, 1705.80, 1309.05, 1173.88, 981.79, 794.58, 458.59, 424.02.

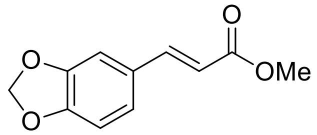

(E)-methyl 3-(benzo[d] [1,3] dioxol-5-yl) acrylate (4ad): This compound was prepared by the general procedure described above and was obtained as white solid (24 h, 85%). R_f (PE: EtOAc = 3:1): 0.5. ^{1}H NMR (500 MHz, $CDCl_3$) δ 7.51 (d, *J* = 15.92 Hz, 1H), 6.95–6.91 (m, 2H), 6.73 (d, *J* = 8.00 Hz, 1H), 6.18 (d, *J* = 15.91 Hz, 1H), 5.92 (s, 2H), 3.71 (s, 3H); ^{13}C NMR (500 MHz, $CDCl_3$) δ 167.60, 149.02, 148.35, 144.56, 128.81, 124.44, 115.72, 108.54, 106.48, 101.57, 51.62. FT-IR (neat, cm^{-1}): 3419.63, 1703.71, 1625.48, 1451.89, 1173.59, 1036.27, 924.51, 520.06.

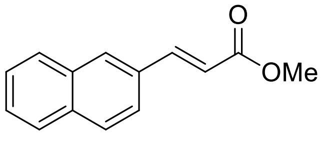

(E)-methyl 3-(naphthalen-2-yl) acrylate (4ae): This compound was prepared by the general procedure described above and was obtained as white solid (24 h, 84%). R_f (PE: EtOAc = 3:1): 0.5; ^{1}H NMR (500 MHz, $CDCl_3$) δ7.78 (d, *J* = 11.18 Hz, 1H), 7.72 (dd , *J* =

7.00 Hz, 4 H), 7.54 (d, J = 8.47 Hz, 1 H), 70.41-7.38 (m, 2H), 6.44 (d, J =15.97 Hz, 1 H), 3.73 (s, 3H); ^{13}C NMR (500 MHz, $CDCl_3$) δ 167.53, 144.95, 131.89, 129.99, 128.72, 128.60, 127.80, 127.27, 126.74, 123.49, 117.95 , 51.76. FT-IR (neat, cm^{-1}): 3418.23, 2948.83, 1714.51, 1438.97, 1271.42, 980.04, 823.22, 474.69.

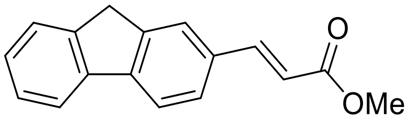

(E)-methyl 3-(9H-fluoren-2-yl) acrylate (4af): This compound was prepared by the general procedure described above and was obtained as white solid (24 h, 80%). R_f (PE: EtOAc = 3:1): 0.5. ^{1}H NMR (500 MHz, $CDCl_3$) δ 7.81–7.76 (m, 3H), 7.72-7.76 (m, 2H), 7.6 (d, J = 7.34 Hz, 1H), 7.43-7.35 (m, 2H), 6.67 (d, J = 16.04 Hz, 1H), 3.94 (s, 2H), 3.74 (s, 3H); ^{13}C NMR (500 MHz, $CDCl_3$) δ 167.31, 145.41, 144.25, 144.09, 128.25, 127.99, 127.39, 125.73, 125.31, 121.08, 120.84, 117.32, 51.91, 36.71. FT-IR (neat, cm^{-1}): 2942.89, 1714.88, 1605.97, 1317.78, 1170.00, 834.06, 741.95.

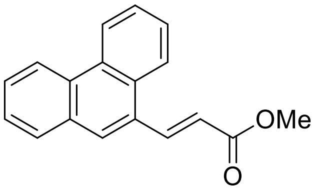

(E)-methyl 3-(phenanthren-9-yl) acrylate (4ag): This compound was prepared by the general procedure described above and was obtained as white solid (24 h, 85%). R_f (PE: EtOAc = 3:1): 0.5. ^{1}H NMR (500 MHz, $CDCl_3$) δ 8.58-8.50 (m, 2H), 8.40 (d, J = 15.69 Hz, 1H), 8.07 (d, J = 7.60 Hz, 1H), 7.82-7.75 (m, 2H), 6.67-7.48 (m, 4H), 6.48 (d, J = 15.67 Hz, 1H), 3.77 (s, 3H); ^{13}C NMR (500 MHz, $CDCl_3$) δ 167.29, 129.23, 127.67, 127.08, 127.04, 126.95, 126.54, 124.33, 123.21, 122.62, 51.87. FT-IR (neat, cm^{-1}): 3087.67, 2965.12, 1715.58, 1609.76, 1306.69, 1162.70, 972.00, 724.73, 421.75.

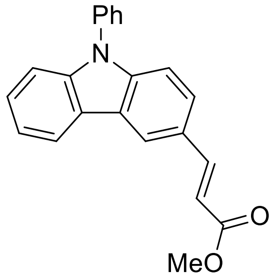

(E)-methyl 3-(9-phenyl-9H-carbazol-3-yl) acrylate (4ah): This compound was prepared by the general procedure described above and was obtained as colorless oil (24 h, 87%). R_f (PE: EtOAc = 3:1): 0.5. ESI *m/z* 350.1150 $[M+Na]^+$. ^{1}H NMR (500 MHz, $CDCl_3$) δ 8.20(s, 1H), 8.05(d, *J* =7.66Hz, 1H), 7.82(d, *J* = 15.89Hz, 1H), 7.51 (d, *J* = 12.00 Hz, 3H), 7.42 (d, *J*=12.85 Hz, 3H), 7.33-7.24 (m, 4H), 6.4 (d, *J* = 15.91 Hz, 1H), 3.73 (s, 3H); ^{13}C NMR (500 MHz, $CDCl_3$) δ 167.98, 130.03, 127.92, 127.08, 126.59, 125.95, 120.65, 120.48, 114.96, 110.23, 110.17, 51.61. FT-IR (neat, cm^{-1}): 3390.64, 3056.60, 2616.10, 1709.98, 1467.72, 1456.43, 686.44, 456.97.

Copies of the ^{1}H and ^{13}C NMR spectra

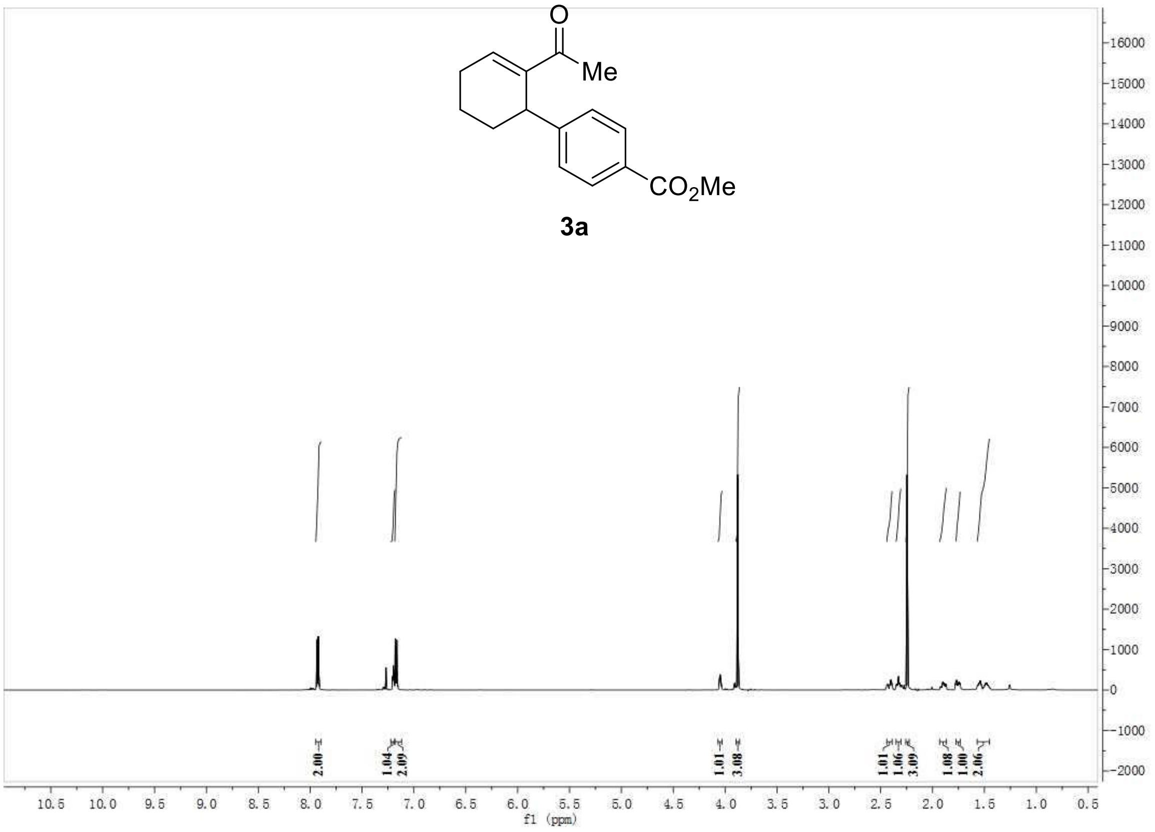

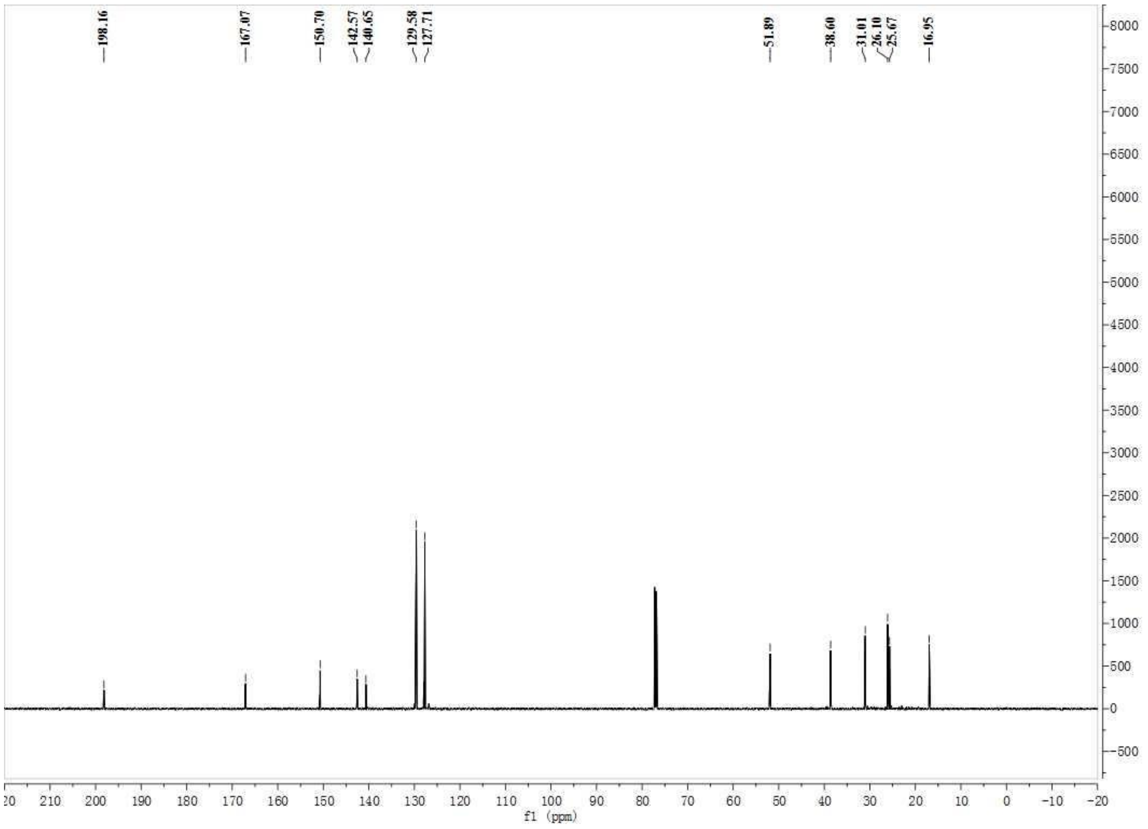

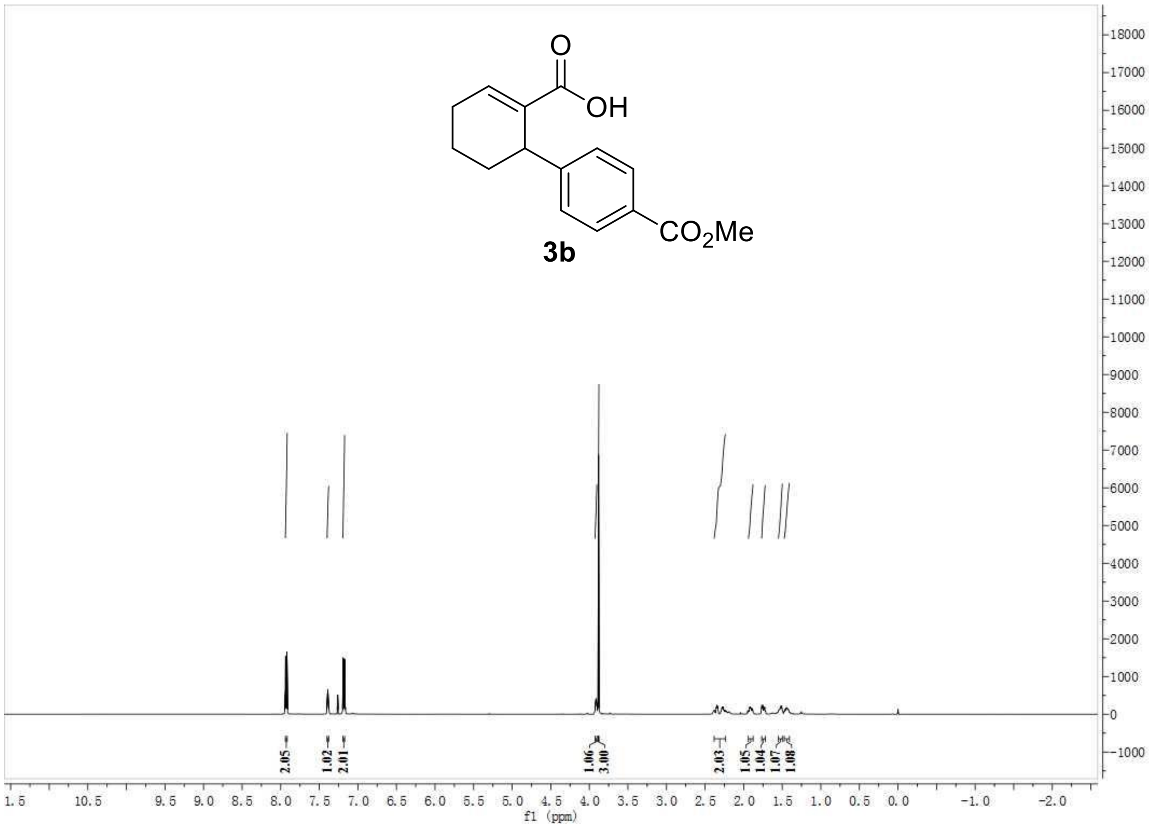

OH
O
CO2Me
3b
f1 (ppm)

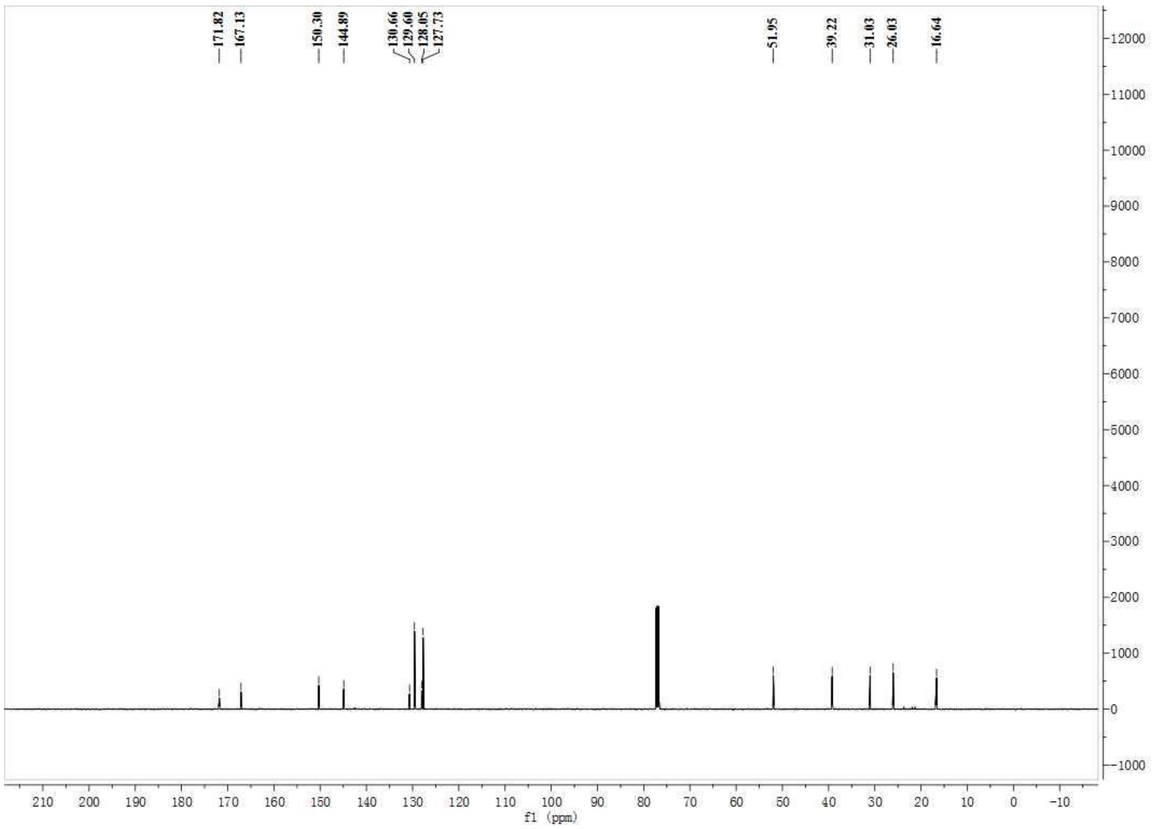

171.82
167.13
150.30
144.89
130.66
129.60
128.05
127.73
51.95
39.22
31.03
26.03
16.64
f1 (ppm)

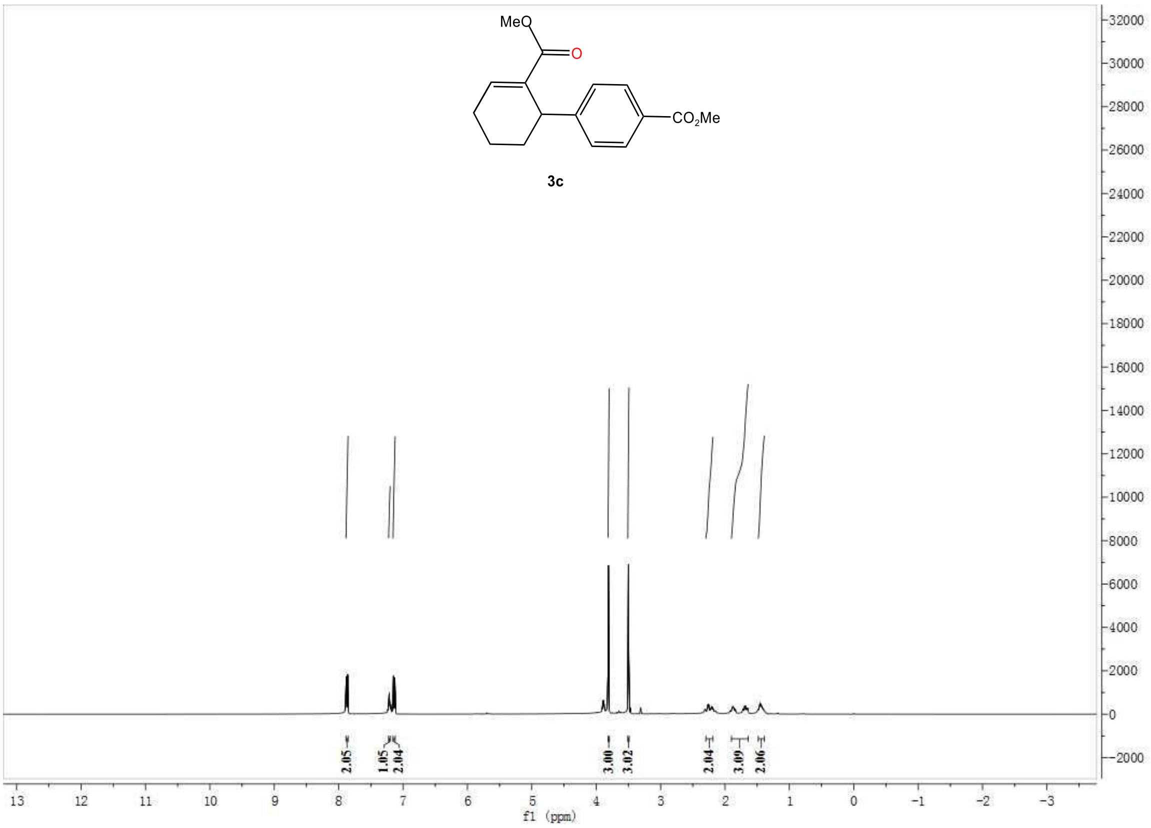
MeO
O
CO2Me
3c
2.05
1.05
2.04
3.00
3.02
2.04
3.09
2.06
f1 (ppm)

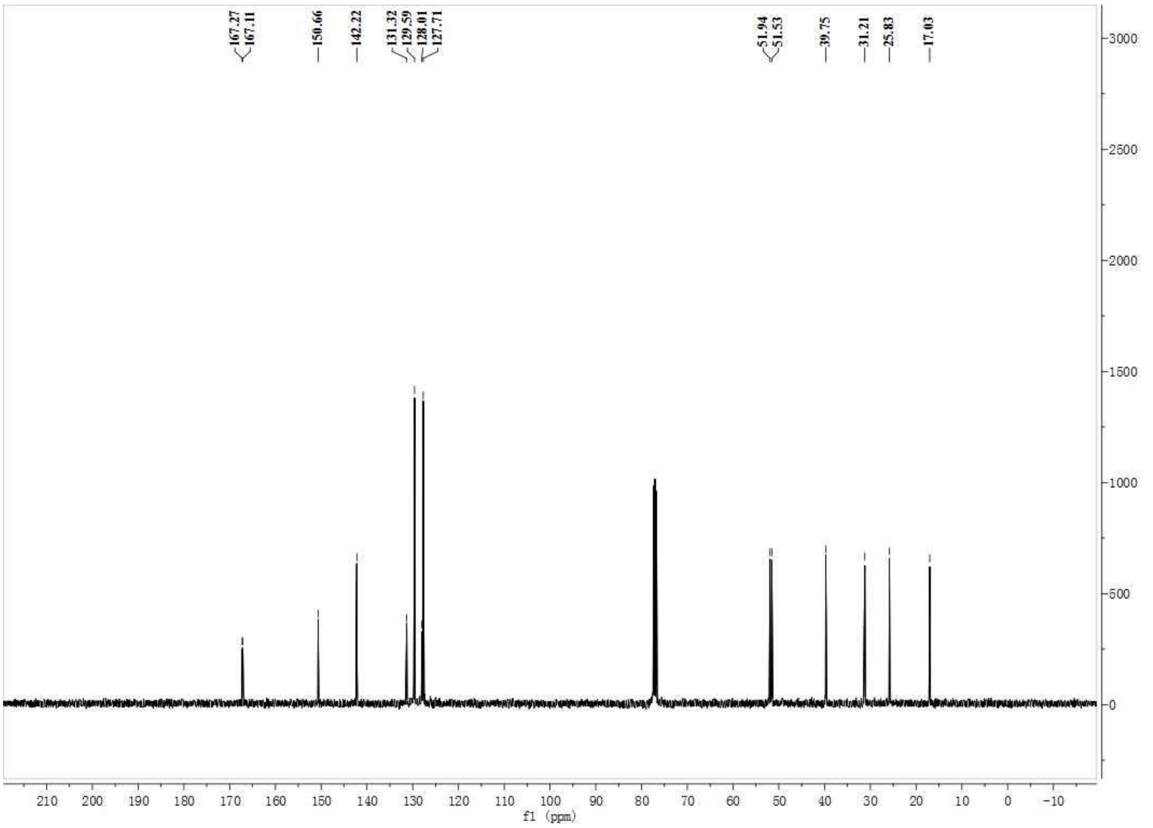
167.27
167.11
150.66
142.22
131.32
129.59
128.01
127.71
51.94
51.53
39.75
31.21
25.83
17.03
f1 (ppm)

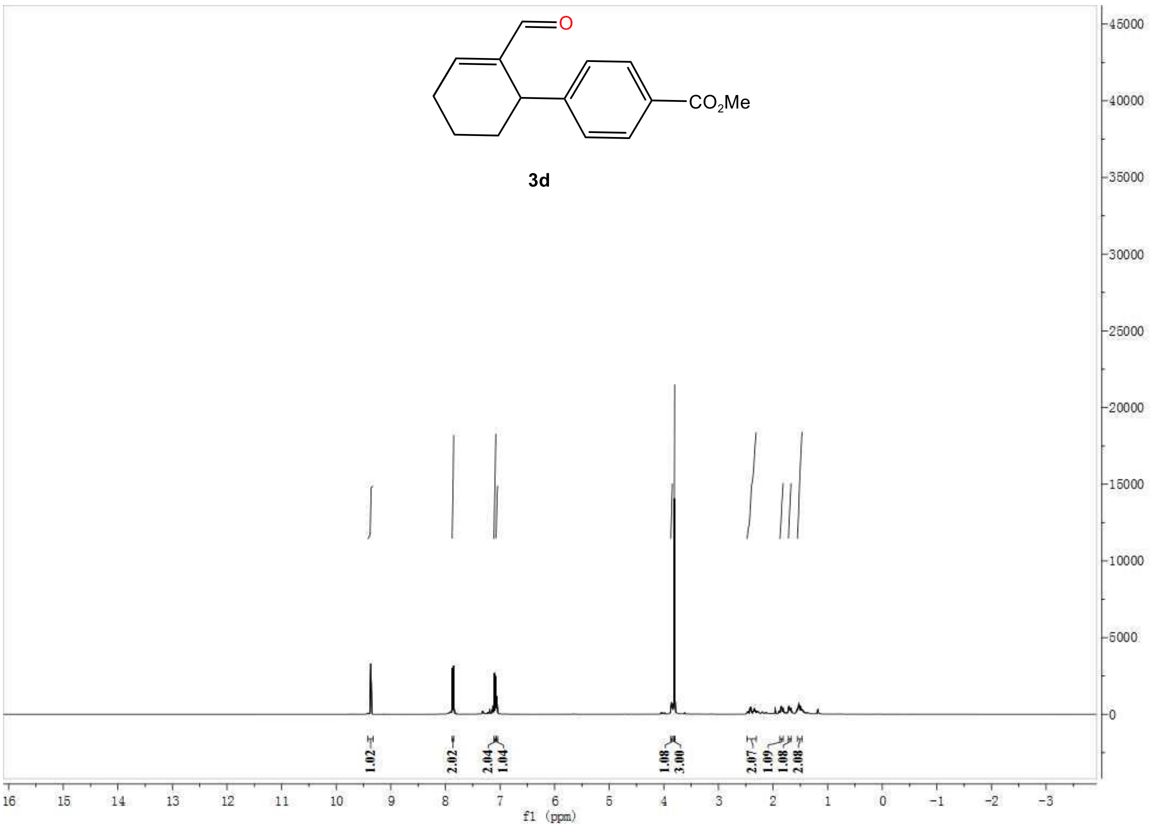

O
CO2Me
3d
1.02
2.02
2.04
1.04
1.08
3.00
2.07
1.09
1.08
2.08
f1 (ppm)

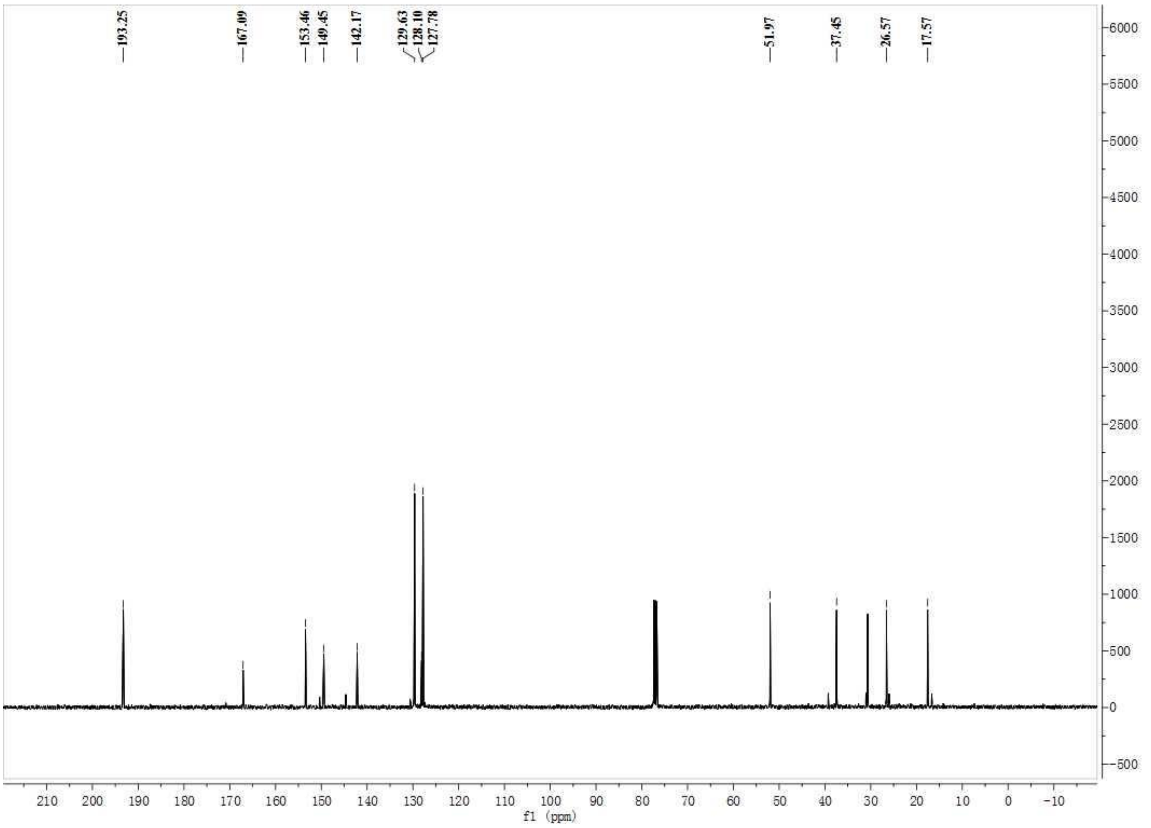

193.25
167.09
153.46
149.45
142.17
129.63
128.10
127.78
51.97
37.45
26.57
17.57
f1 (ppm)

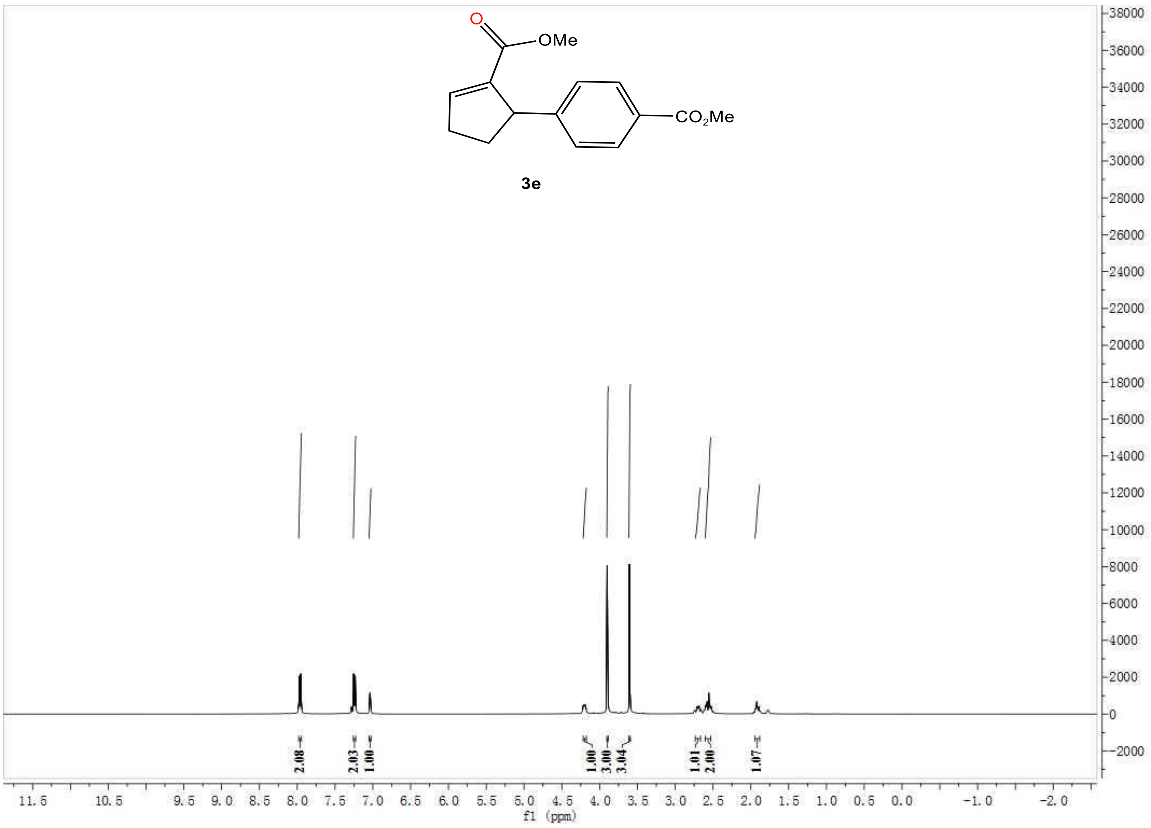

OMe
CO2Me
3e
2.08
2.03
1.00
1.00
3.00
3.04
1.01
2.00
1.07
f1 (ppm)

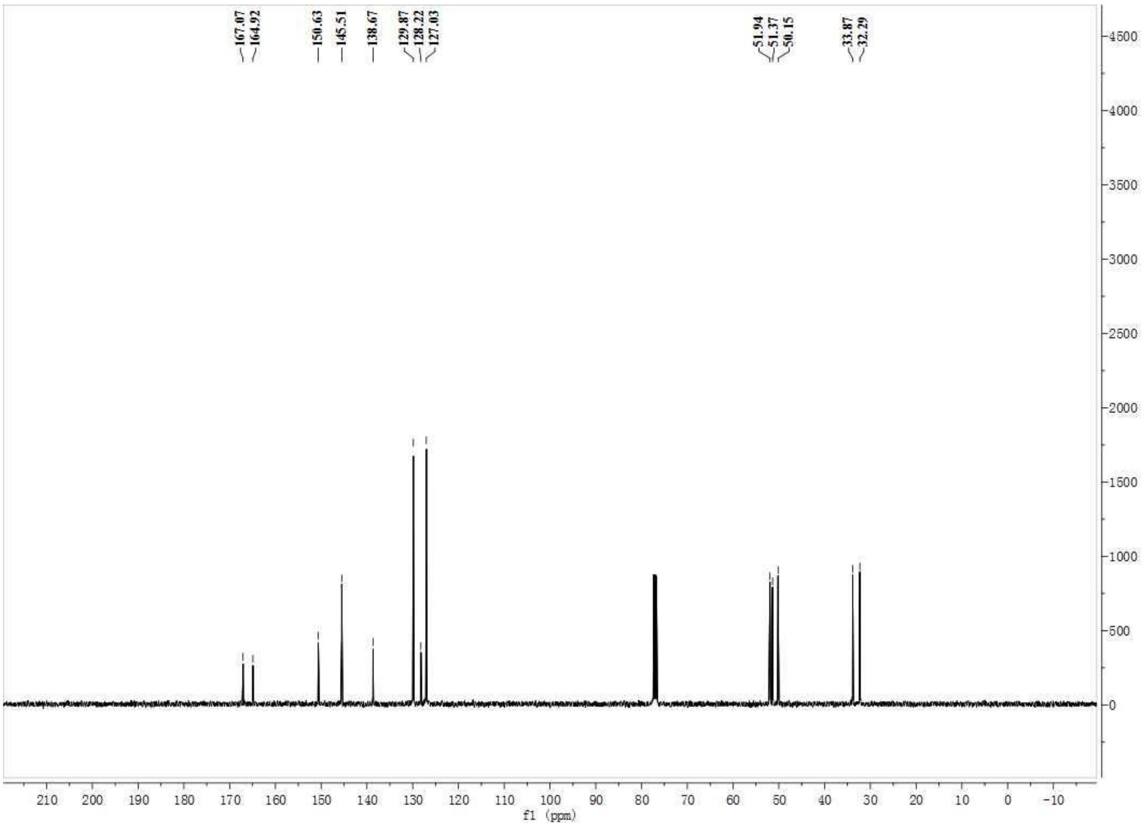

167.07
164.92
150.63
145.51
138.67
129.87
128.22
127.03
51.94
51.37
50.15
33.87
32.29
f1 (ppm)

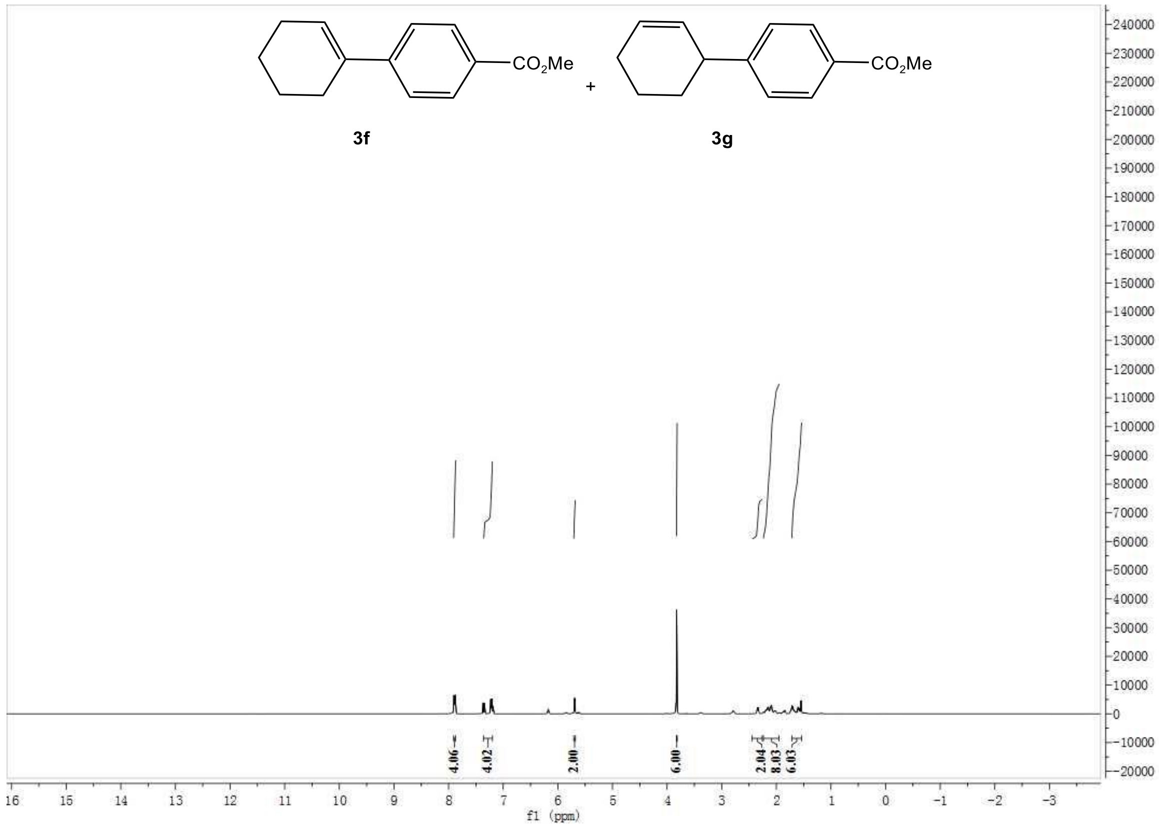
CO2Me
+
CO2Me
3f
3g
4.06
4.02
2.00
6.00
2.04
8.03
6.03
f1 (ppm)

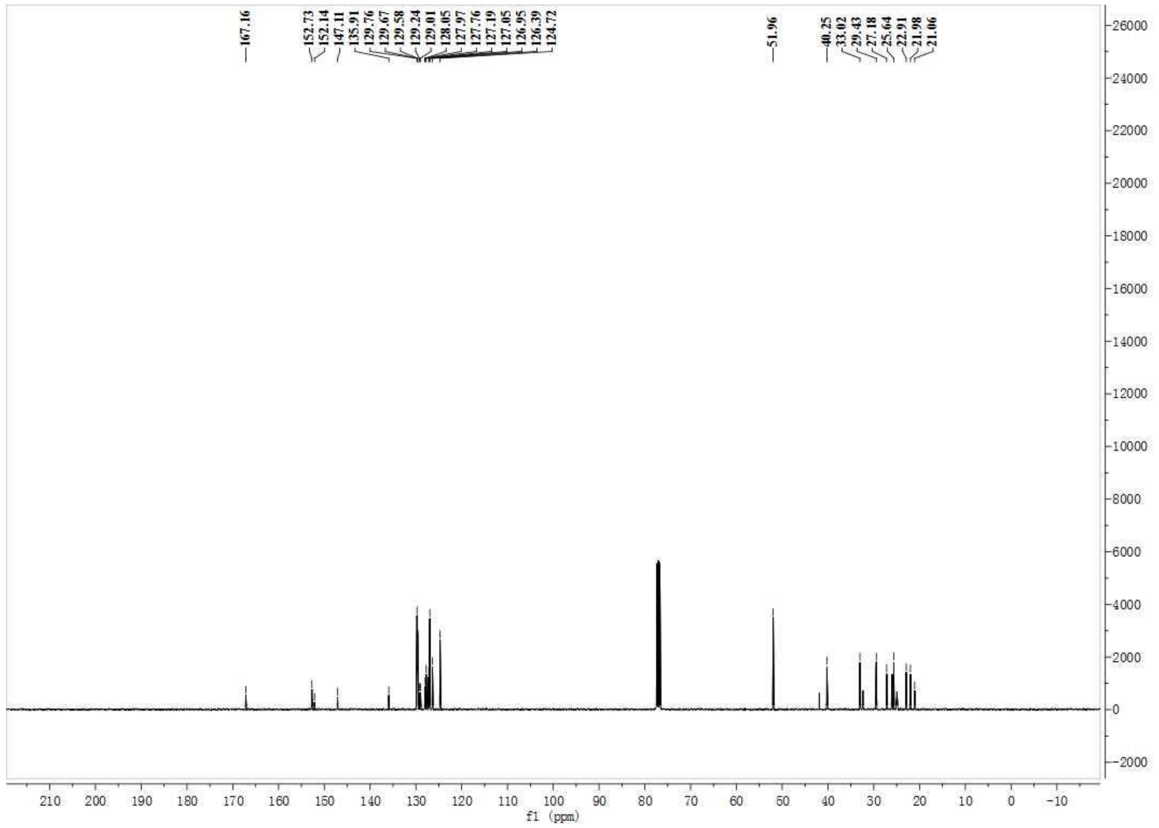
167.16
152.73
152.14
147.11
135.91
129.76
129.67
129.58
129.24
129.01
128.05
127.97
127.76
127.19
127.05
126.95
126.39
124.72
51.96
40.25
33.02
29.43
27.18
25.64
22.91
21.98
21.06
f1 (ppm)

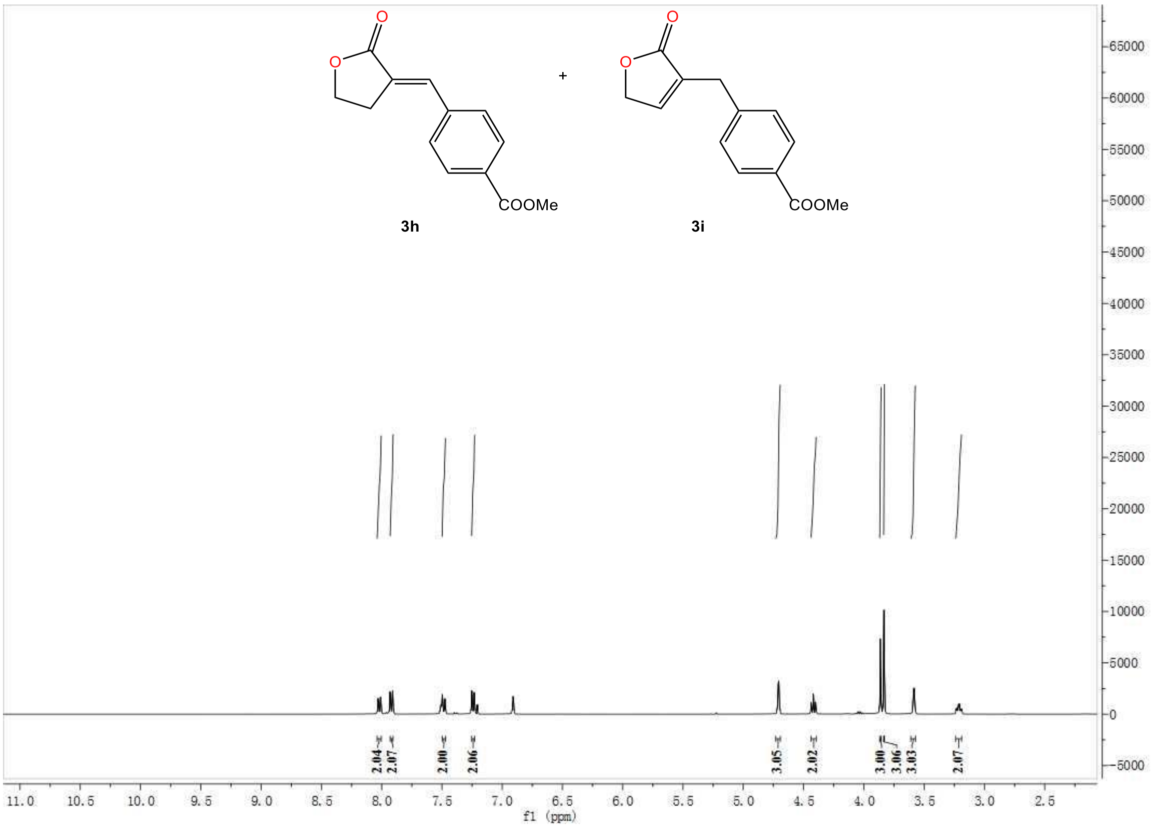
COOMe
3h
COOMe
3i
f1 (ppm)

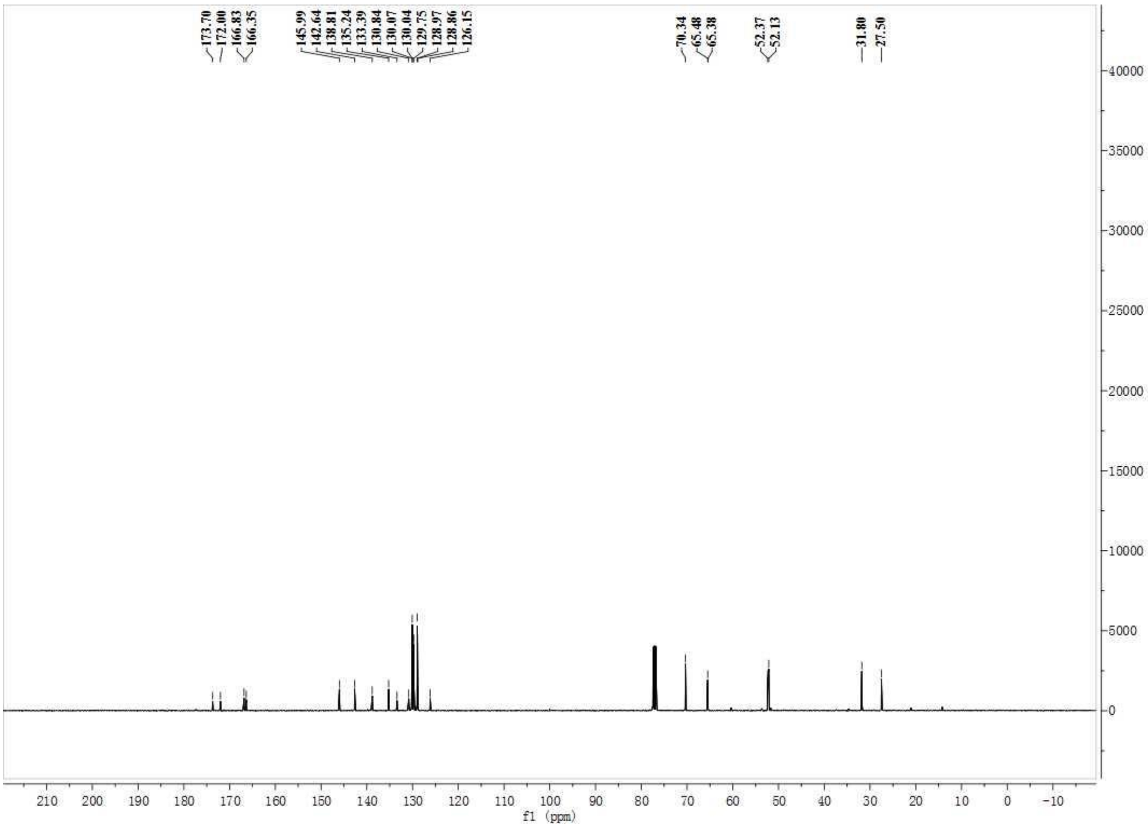
173.70
172.00
166.83
166.35
145.99
142.64
138.81
135.24
133.39
130.84
130.07
130.04
129.75
128.97
128.86
126.15
70.34
65.48
65.38
52.37
52.13
31.80
27.50
f1 (ppm)

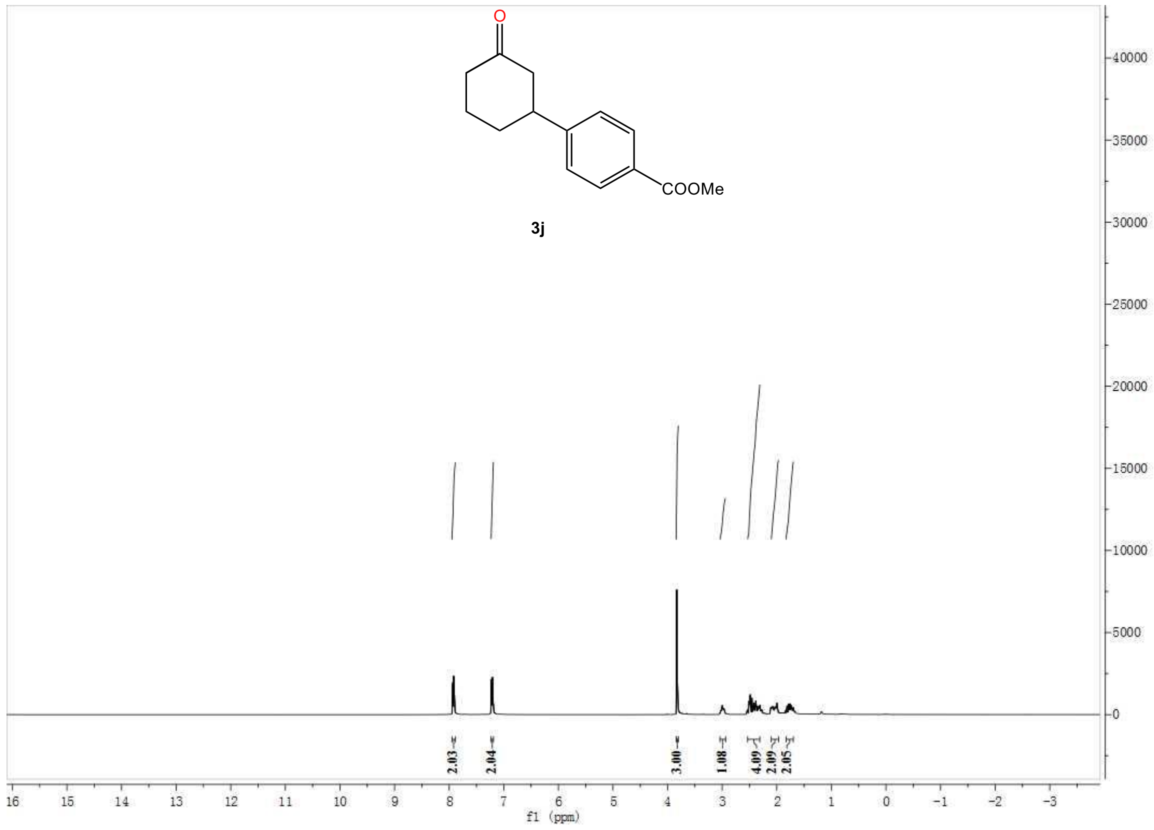
O
COOMe
3j
2.03
2.04
3.00
1.08
4.09
2.09
2.05
f1 (ppm)

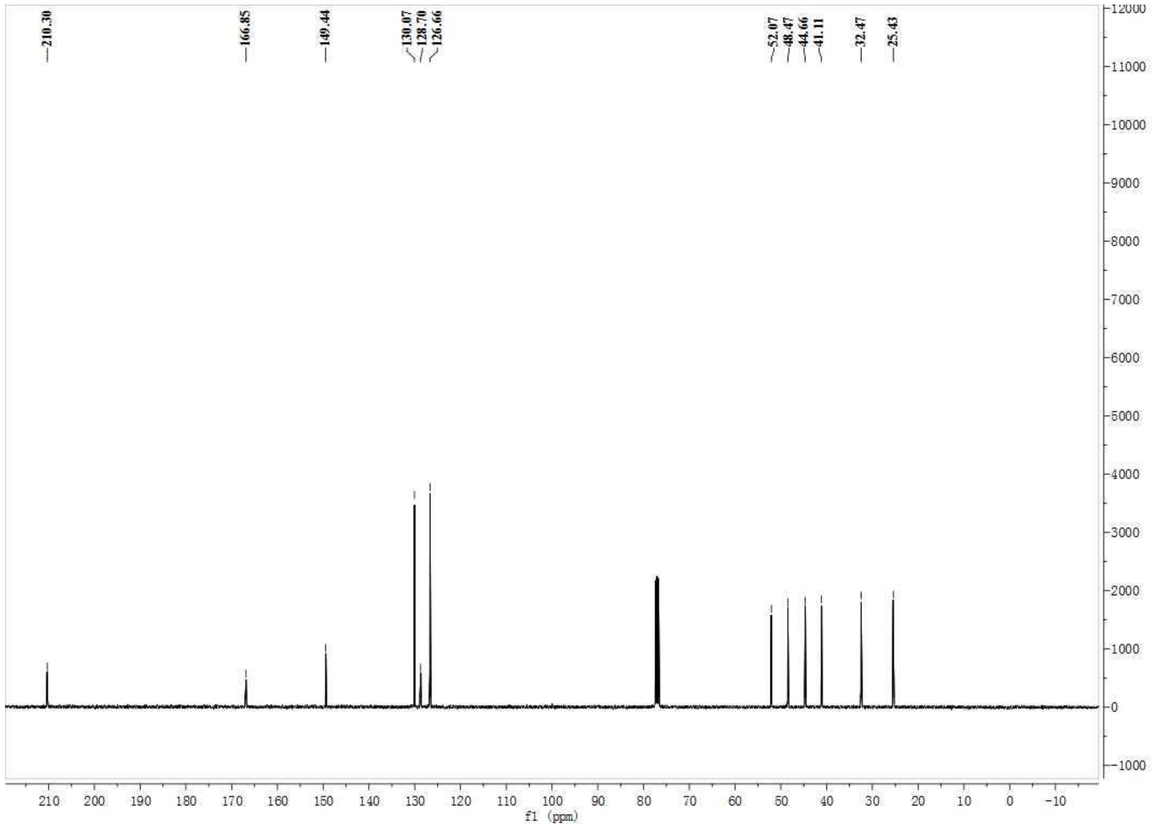
210.30
166.85
149.44
130.07
128.70
126.66
52.07
48.47
44.66
41.11
32.47
25.43
f1 (ppm)

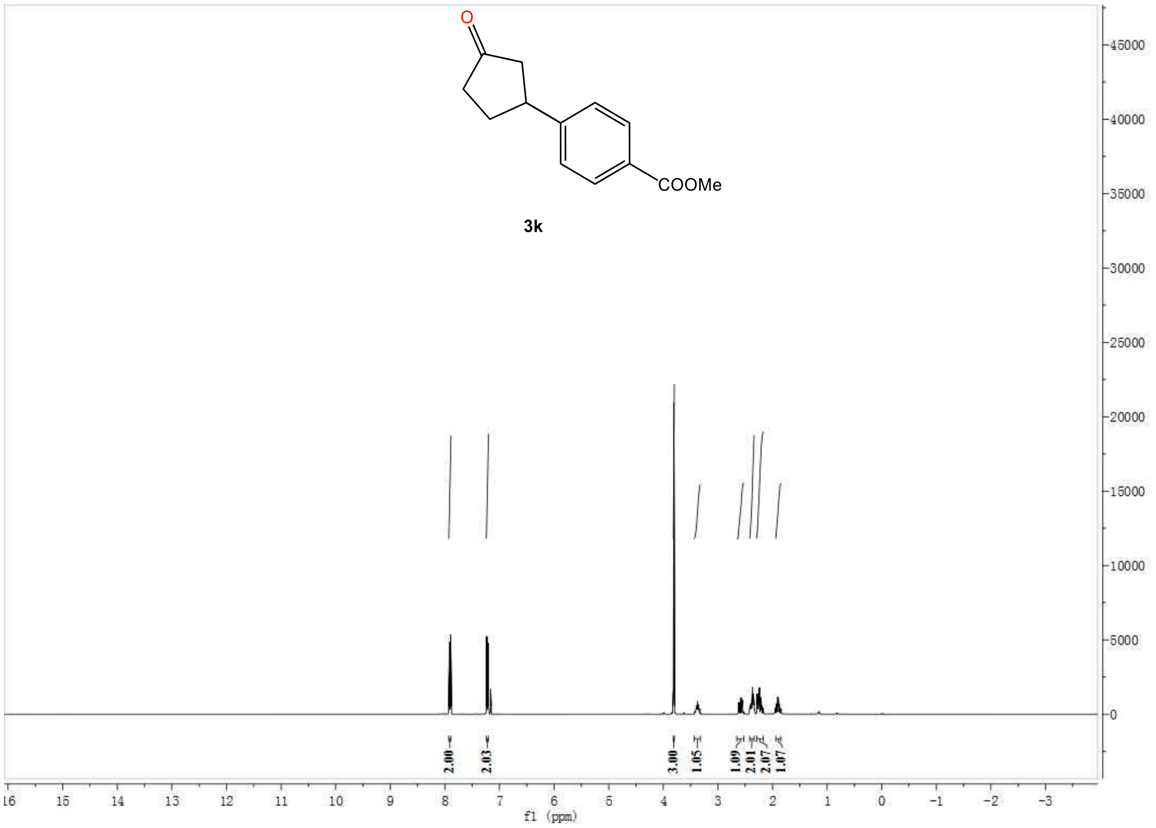

O
COOMe
3k
45000
40000
35000
30000
25000
20000
15000
10000
5000
0
2.00
2.03
3.00
1.05
1.09
2.01
2.07
1.07
16
15
14
13
12
11
10
9
8
7
6
5
4
3
2
1
0
-1
-2
-3
f1 (ppm)

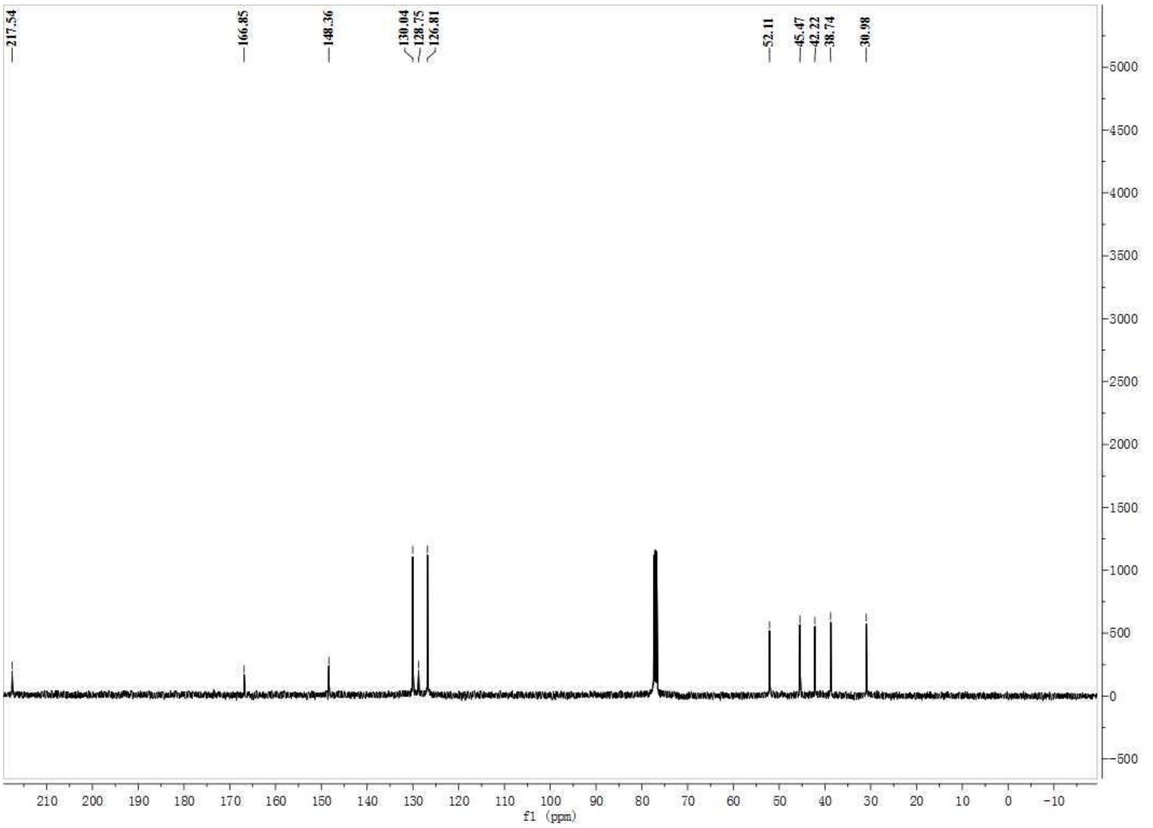

217.54
166.85
148.36
130.04
128.75
126.81
52.11
45.47
42.22
38.74
30.98
5000
4500
4000
3500
3000
2500
2000
1500
1000
500
0
-500
210
200
190
180
170
160
150
140
130
120
110
100
90
80
70
60
50
40
30
20
10
0
-10
f1 (ppm)

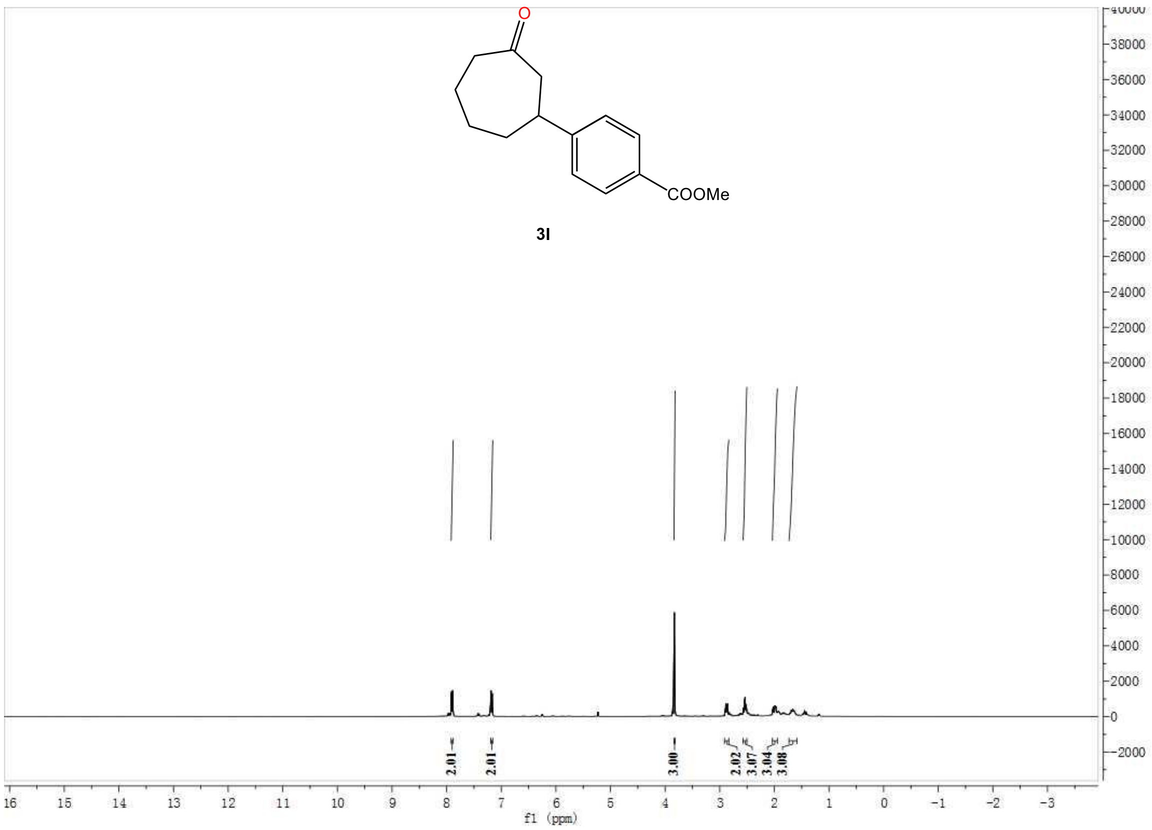
COOMe
3l
2.01
2.01
3.00
2.02
3.07
3.04
3.08
f1 (ppm)

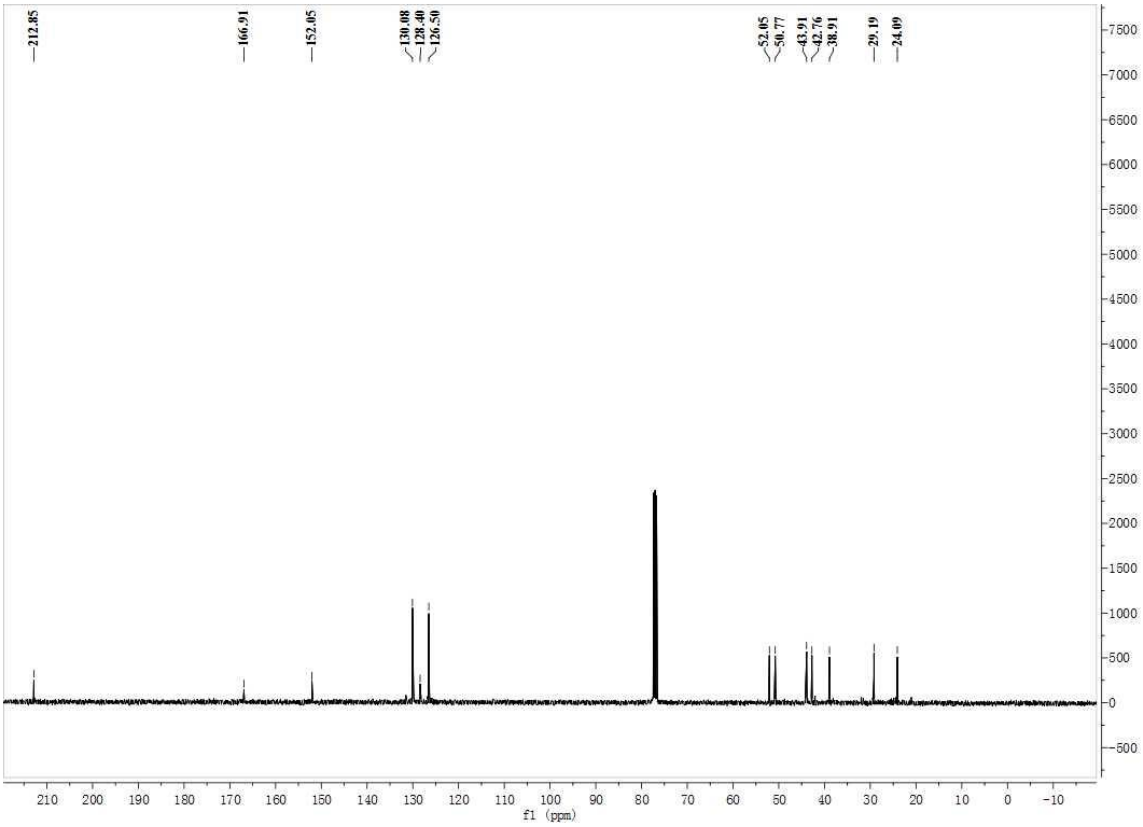
212.85
166.91
152.05
130.08
128.40
126.50
52.05
50.77
43.91
42.76
38.91
29.19
24.09
f1 (ppm)

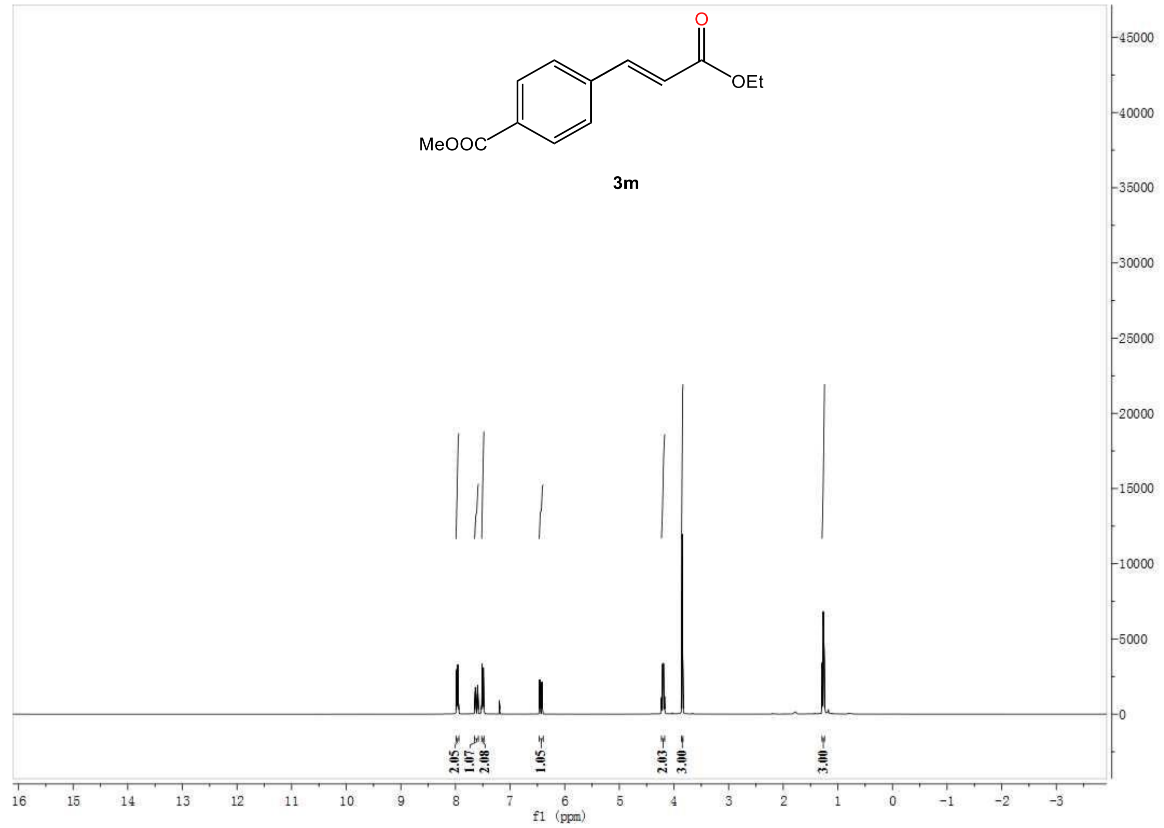
O
OEt
MeOOC
3m
2.05
1.07
2.08
1.05
2.03
3.00
3.00
f1 (ppm)

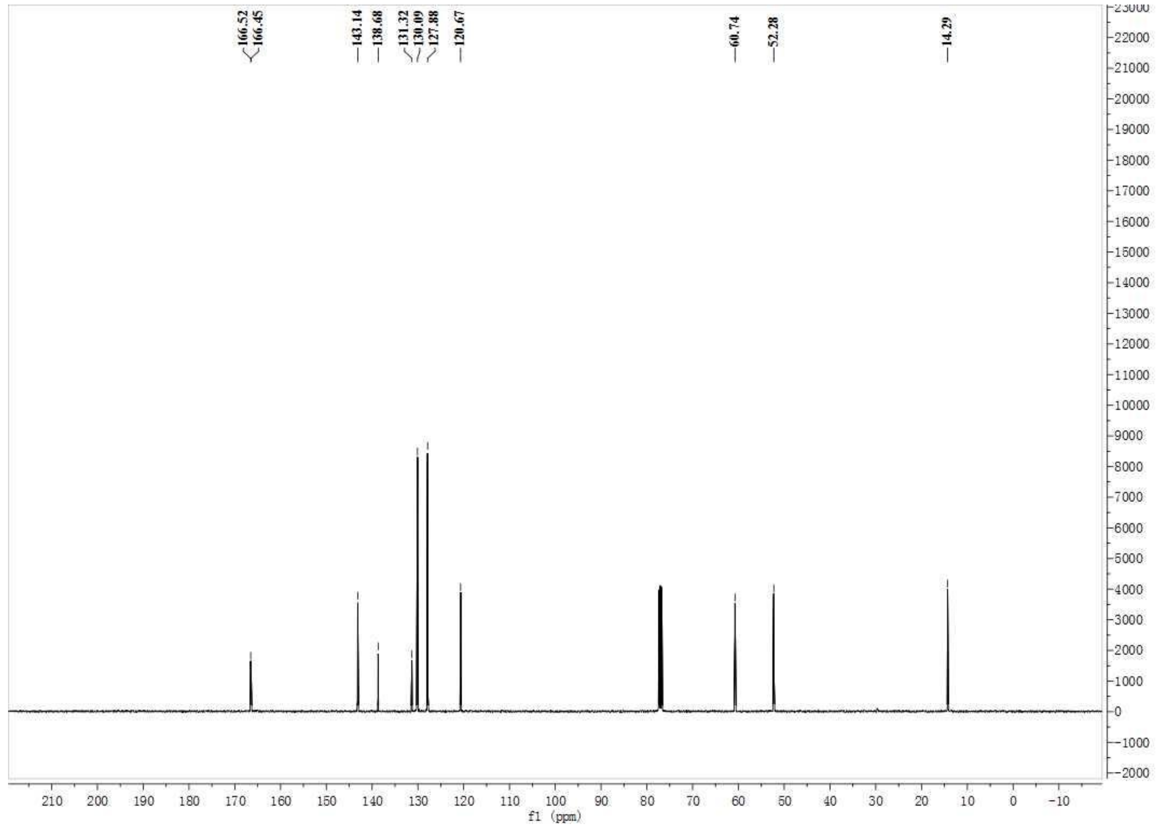
166.52
166.45
143.14
138.68
131.32
130.09
127.88
120.67
60.74
52.28
14.29
f1 (ppm)

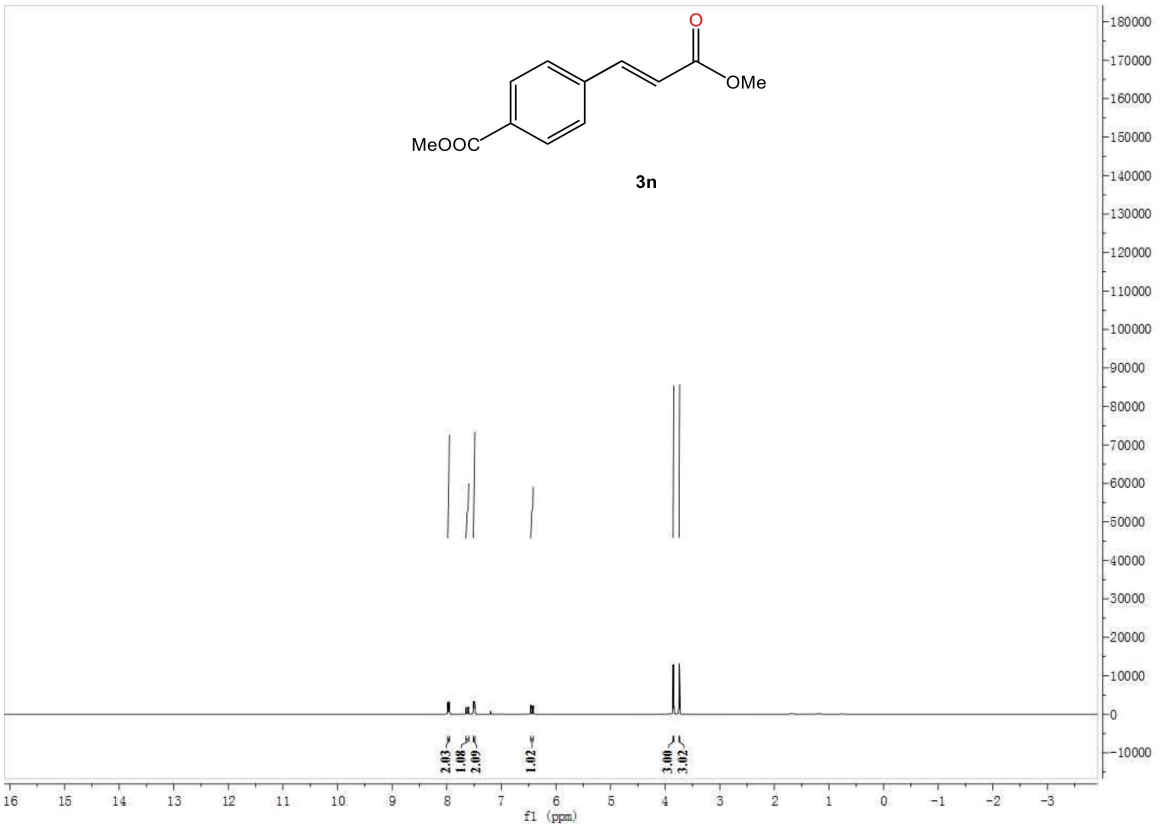
MeOOC
O
OMe
3n
2.03
1.08
2.09
1.02
3.00
3.02
f1 (ppm)

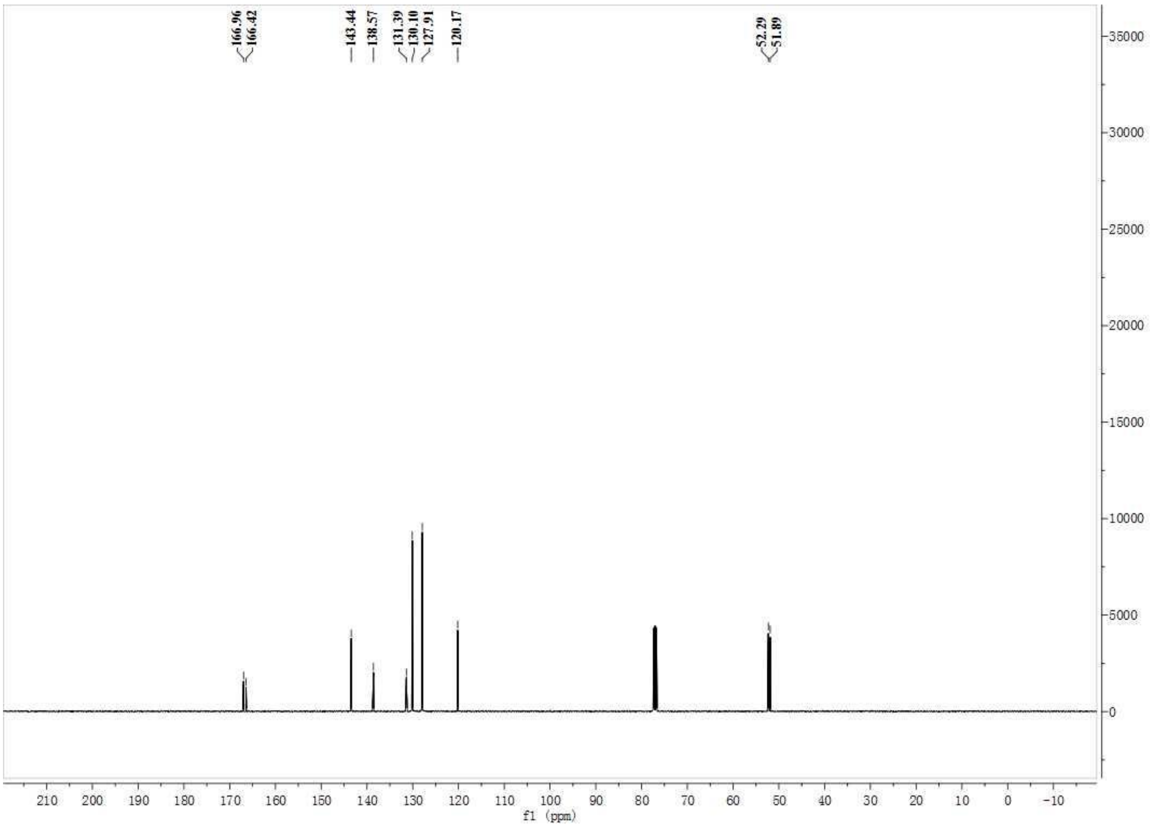
166.96
166.42
143.44
138.57
131.39
130.10
127.91
120.17
52.29
51.89
f1 (ppm)

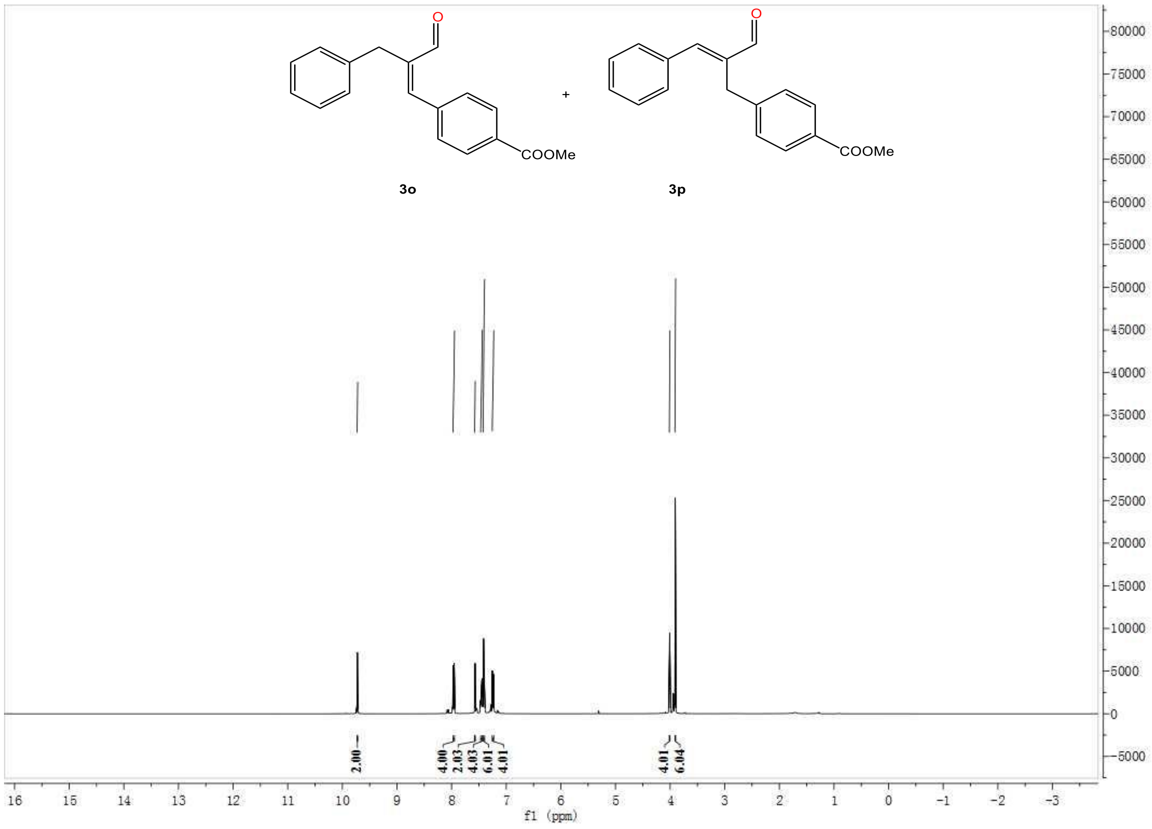
COOMe
COOMe
3o
3p
2.00
4.00
2.03
4.03
6.01
4.01
4.01
6.04
f1 (ppm)

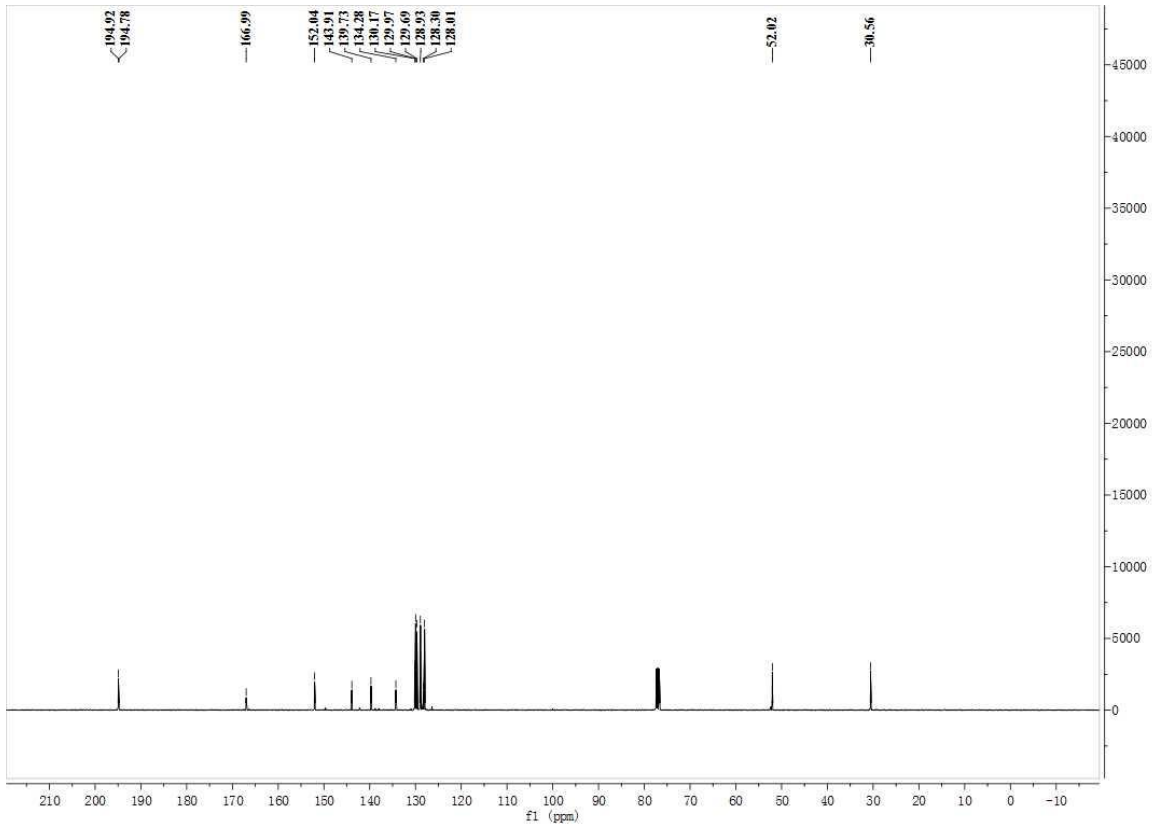
194.92
194.78
166.99
152.04
143.91
139.73
134.28
130.17
129.97
129.69
128.93
128.30
128.01
52.02
30.56
f1 (ppm)

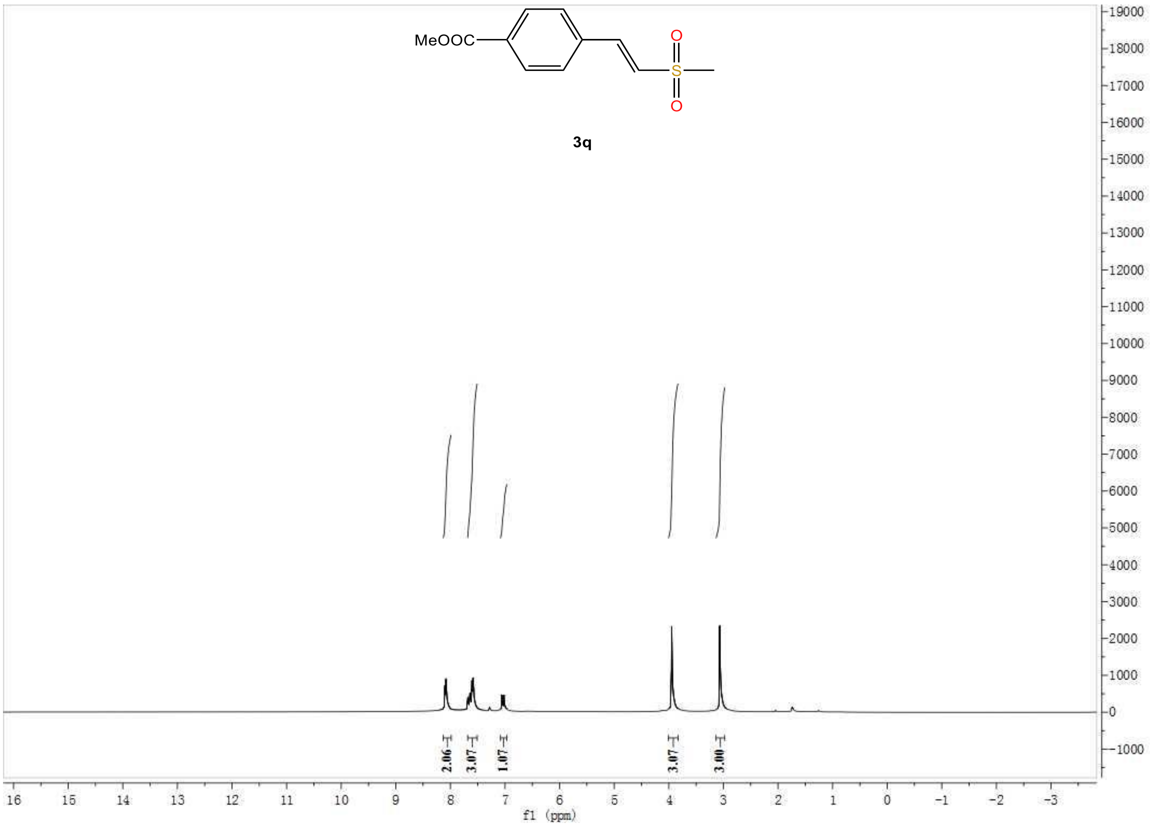
MeOOC
O
S
O
3q
2.06
3.07
1.07
3.07
3.00
f1 (ppm)

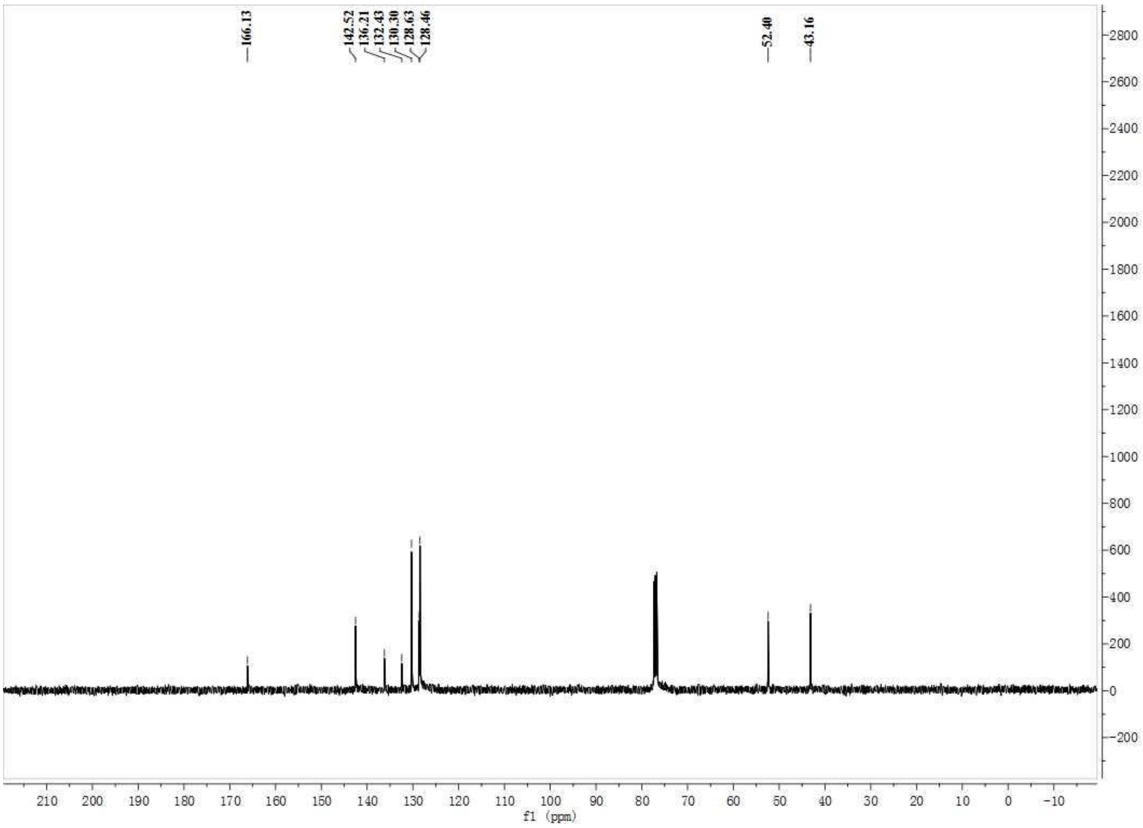
166.13
142.52
136.21
132.43
130.30
128.63
128.46
52.40
43.16
f1 (ppm)

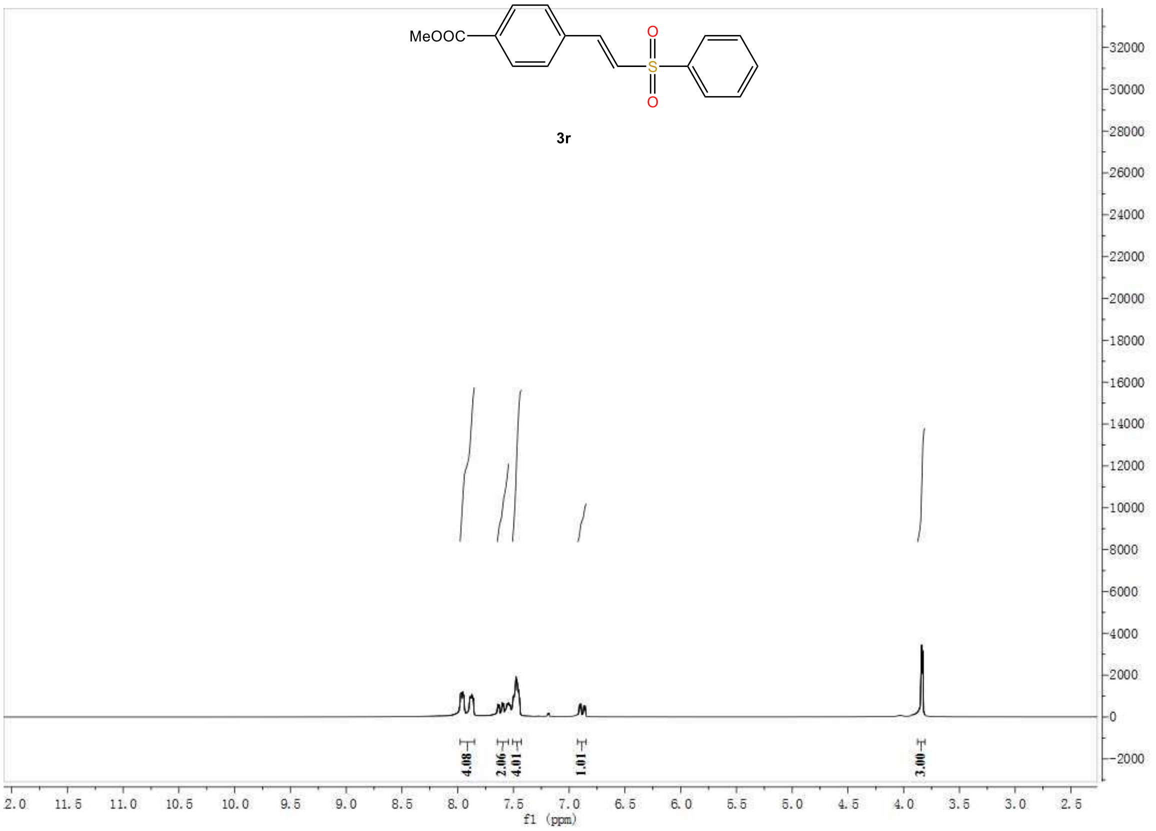
MeOOC
3r
4.08
2.06
4.01
1.01
3.00
f1 (ppm)

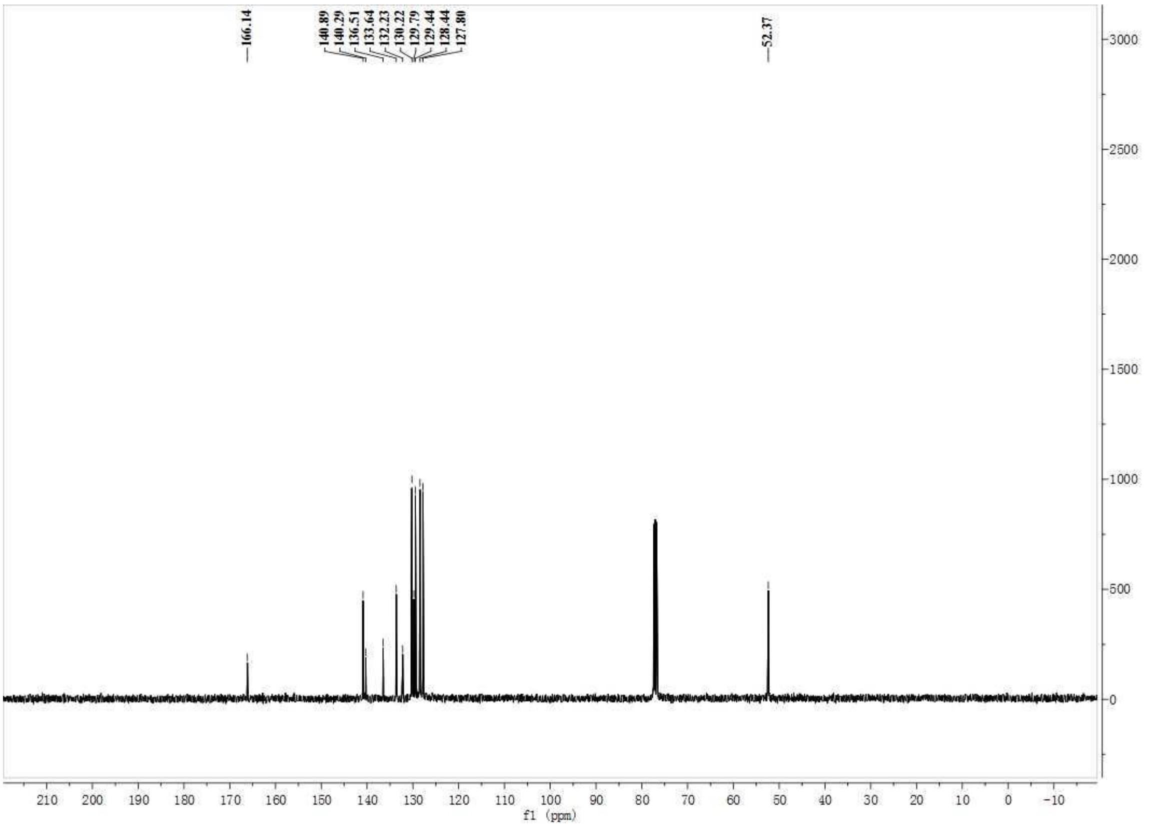
166.14
140.89
140.29
136.51
133.64
132.23
130.22
129.79
129.44
128.44
127.80
52.37
f1 (ppm)

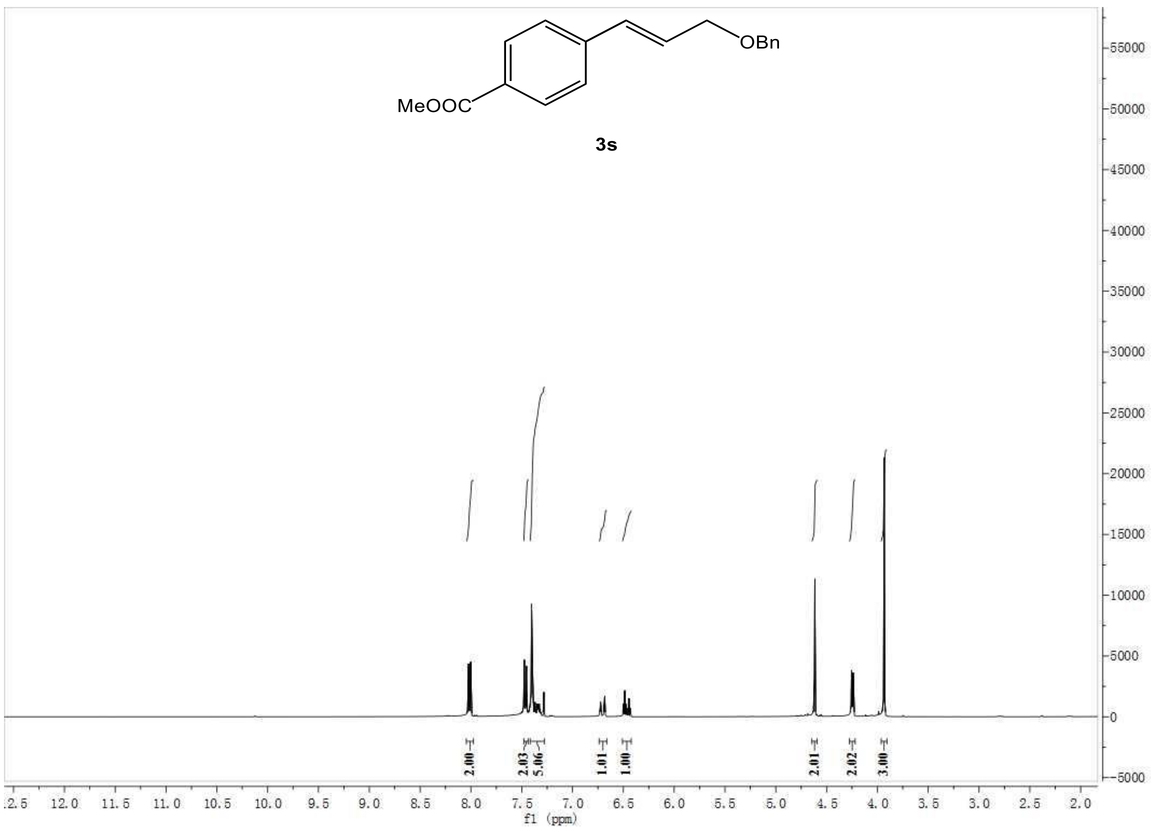
OBn
MeOOC
3s
2.00
2.03
5.06
1.01
1.00
2.01
2.02
3.00
f1 (ppm)

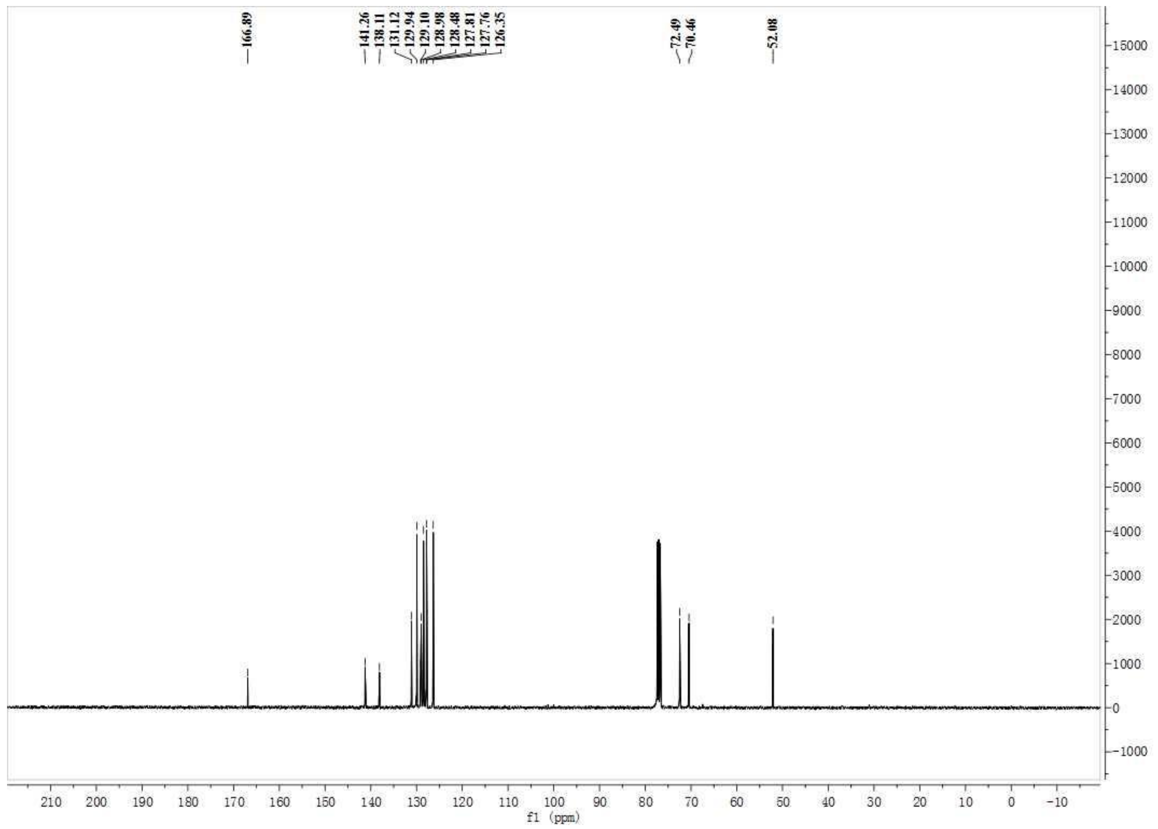
166.89
141.26
138.11
131.12
129.94
129.10
128.98
128.48
127.81
127.76
126.35
72.49
70.46
52.08
f1 (ppm)

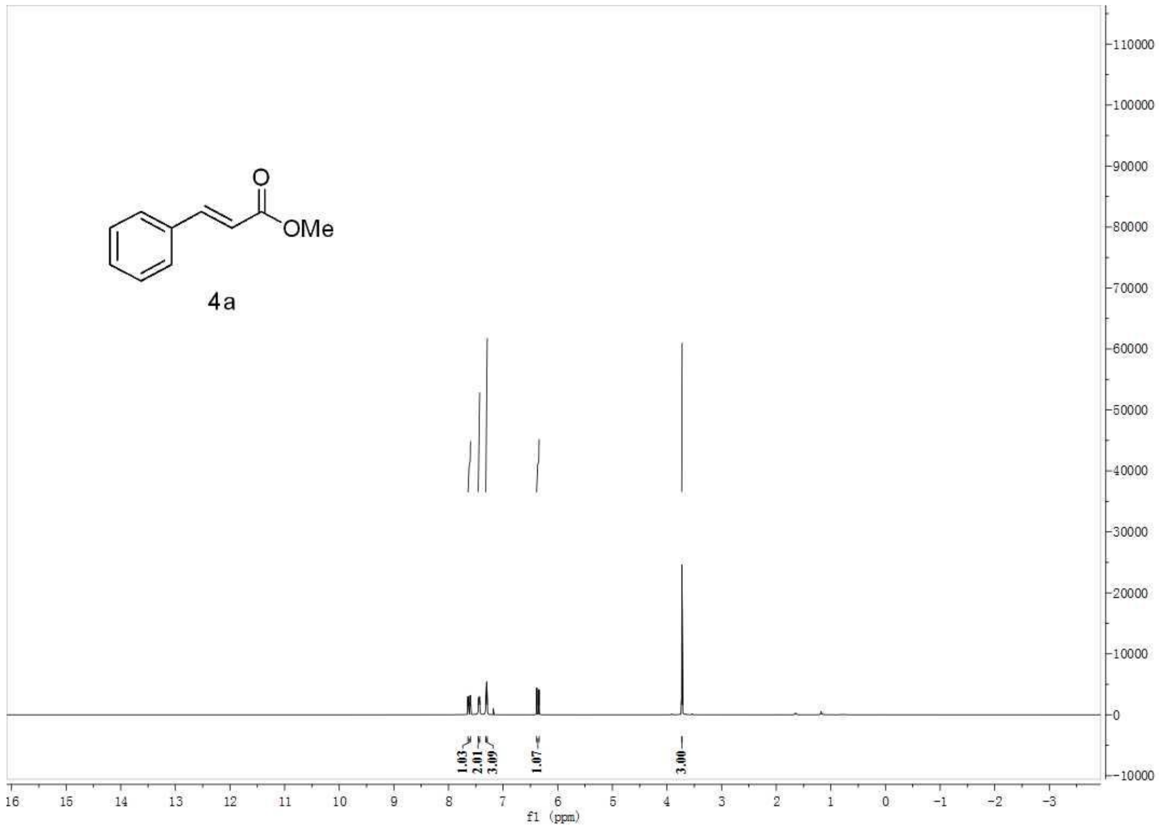
OMe
O
4a
1.03
2.01
3.09
1.07
3.00
f1 (ppm)

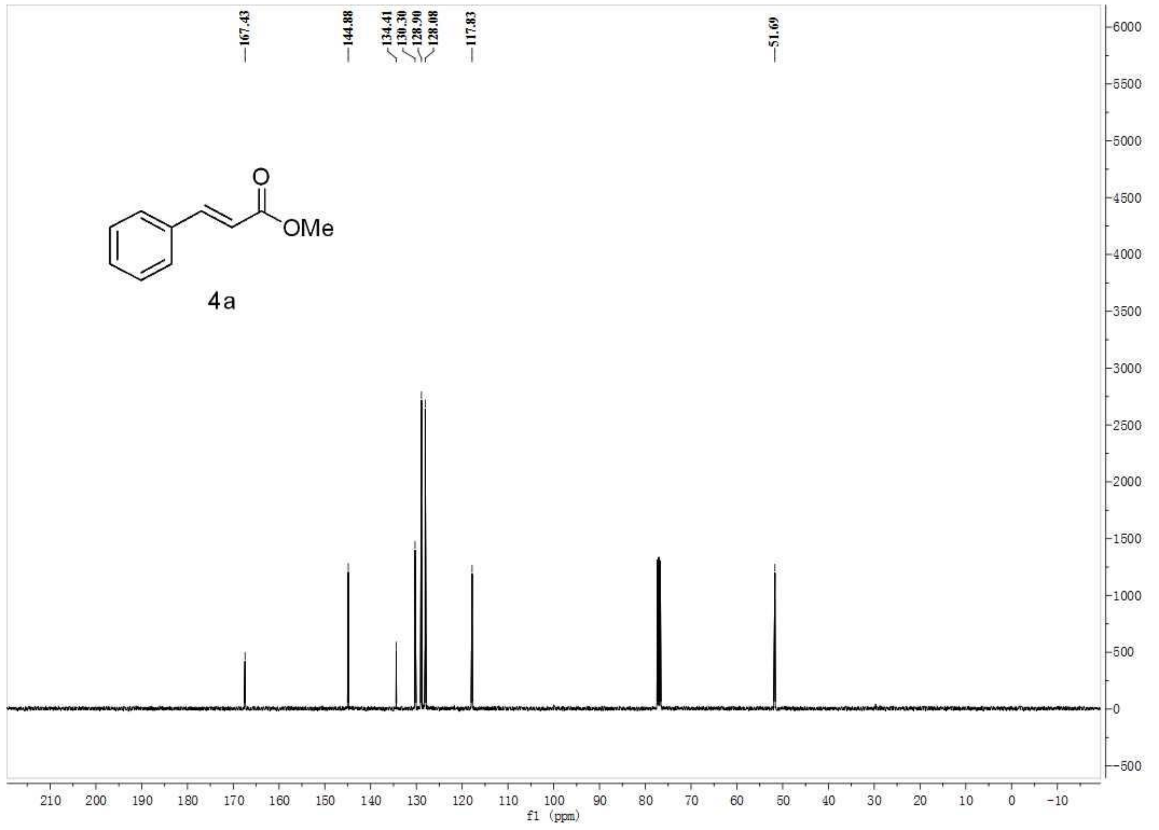
167.43
144.88
134.41
130.30
128.90
128.08
117.83
51.69
OMe
O
4a
f1 (ppm)

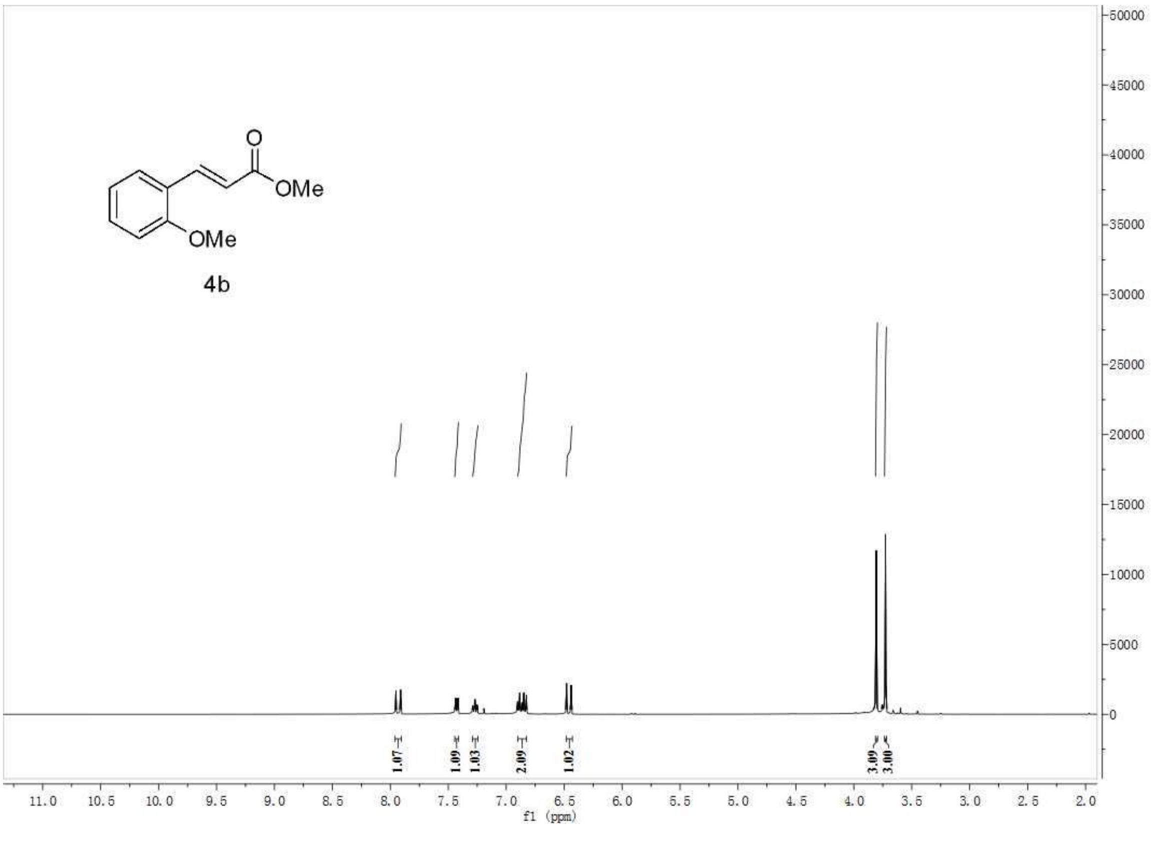
OMe
OMe
4b
1.07
1.09
1.03
2.09
1.02
3.09
3.00
f1 (ppm)

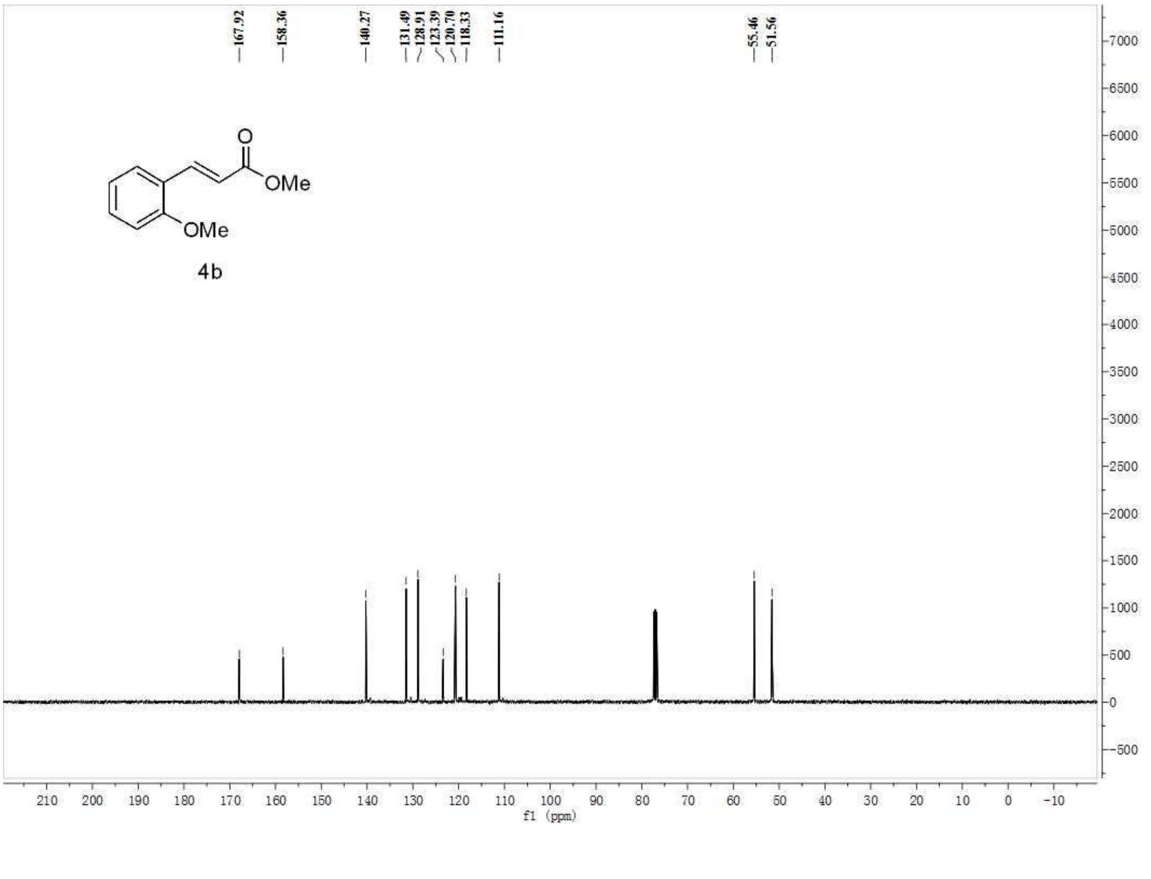
167.92
158.36
140.27
131.49
128.91
123.39
120.70
118.33
111.16
55.46
51.56
OMe
OMe
4b
f1 (ppm)

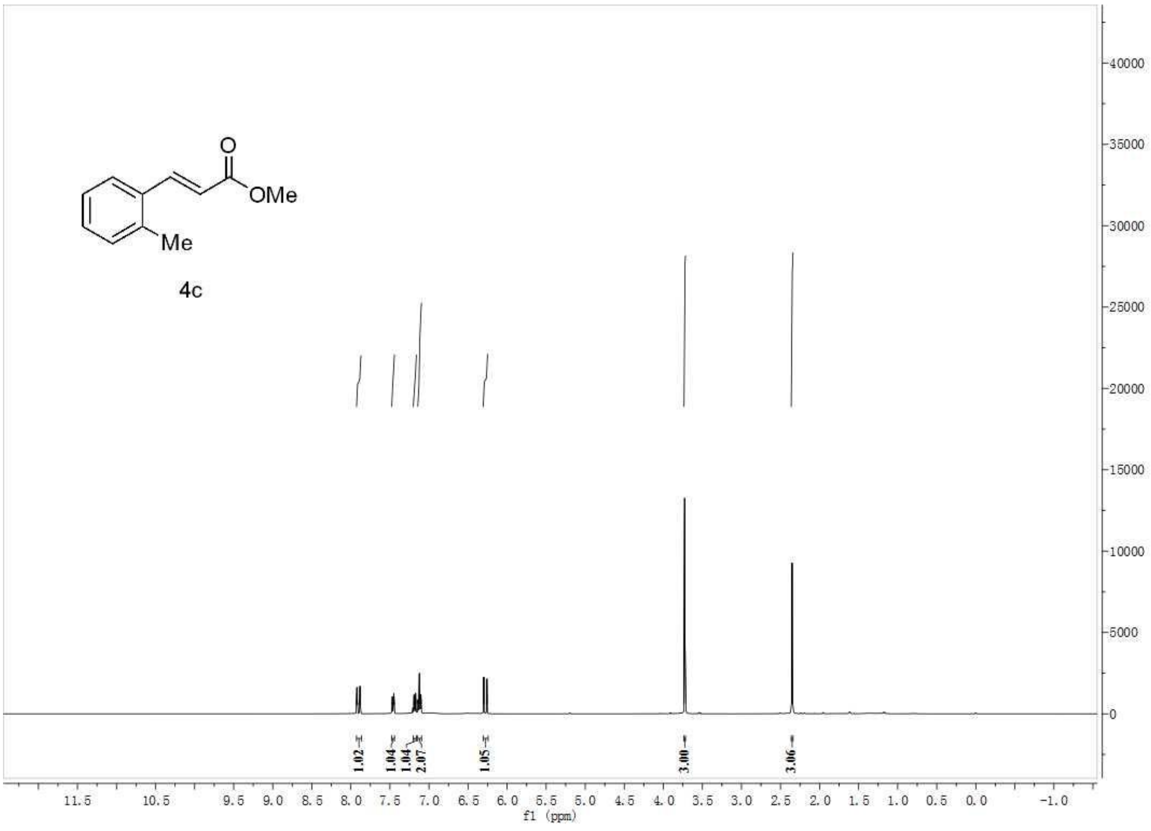

OMe
Me
1.02
1.04
1.04
2.07
1.05
3.00
3.06
f1 (ppm)

4c

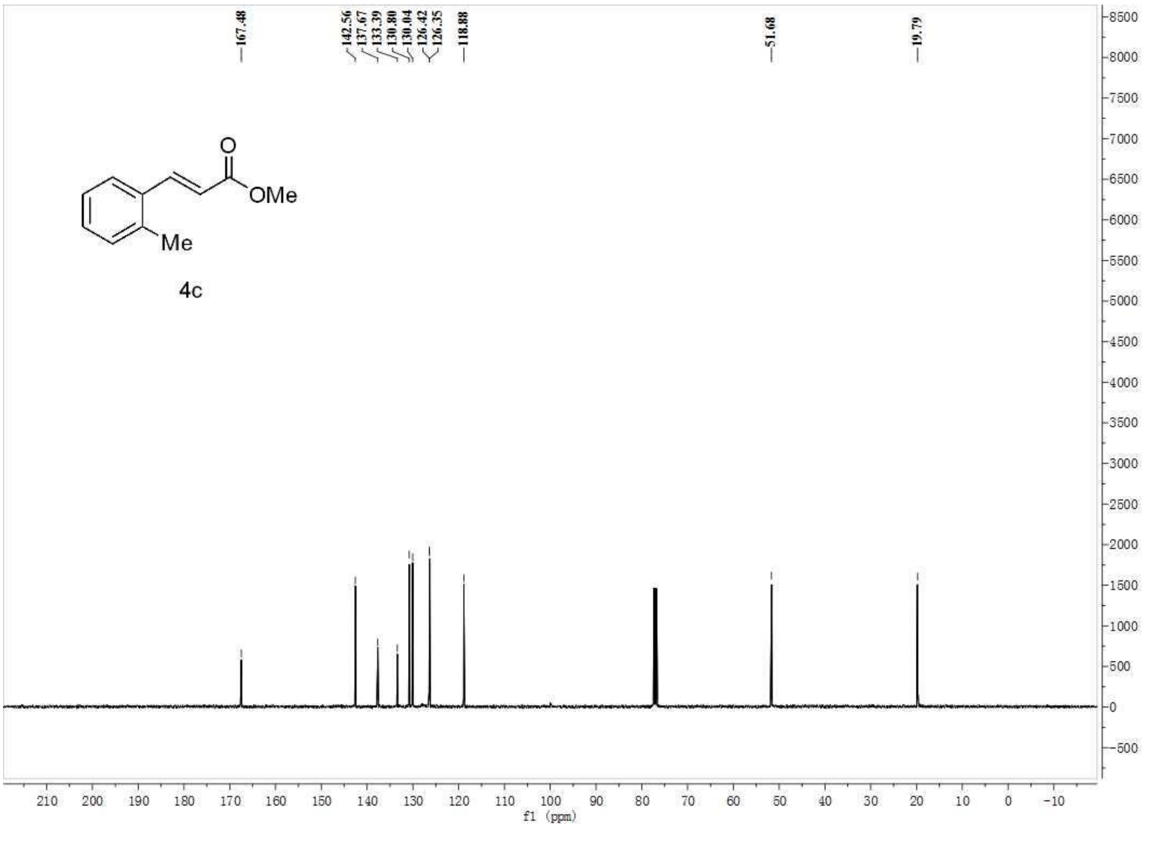

167.48
142.56
137.67
133.39
130.80
130.04
126.42
126.35
118.88
51.68
19.79
OMe
Me
f1 (ppm)

4c

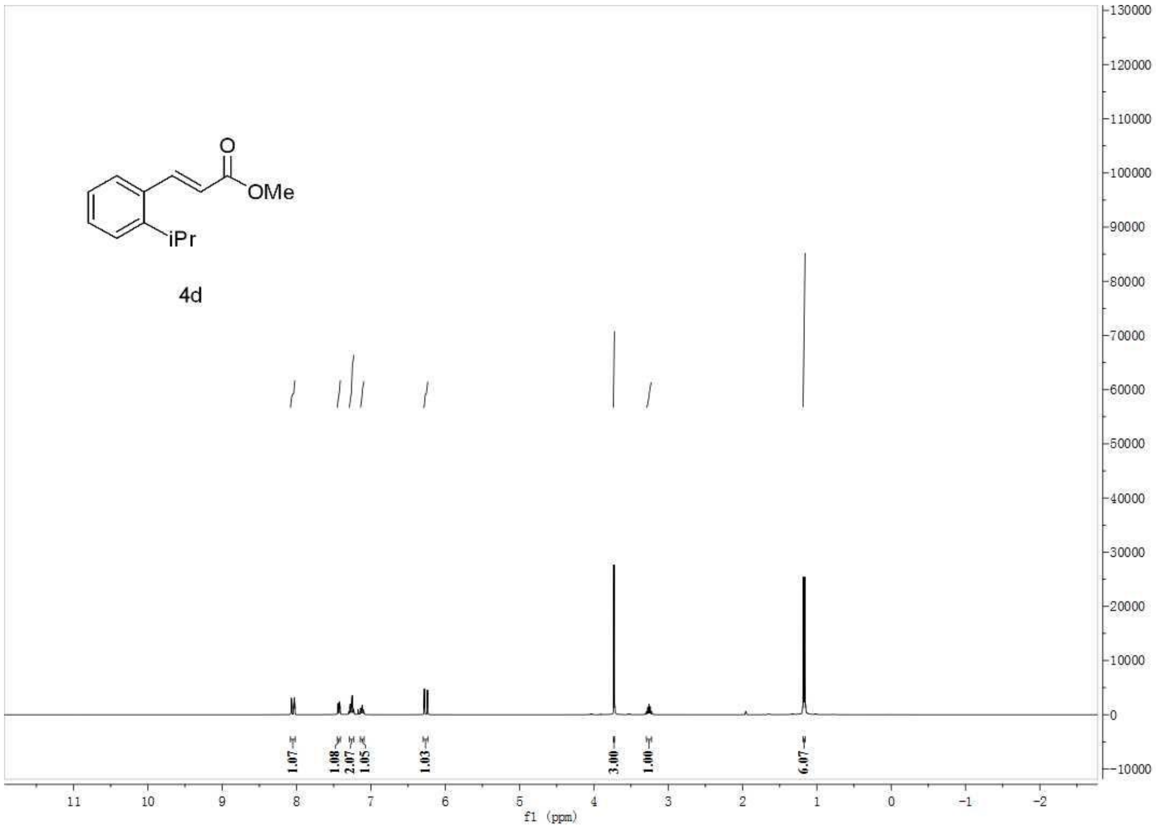
OMe
iPr
4d
1.07
1.08
2.07
1.05
1.03
3.00
1.00
6.07
f1 (ppm)

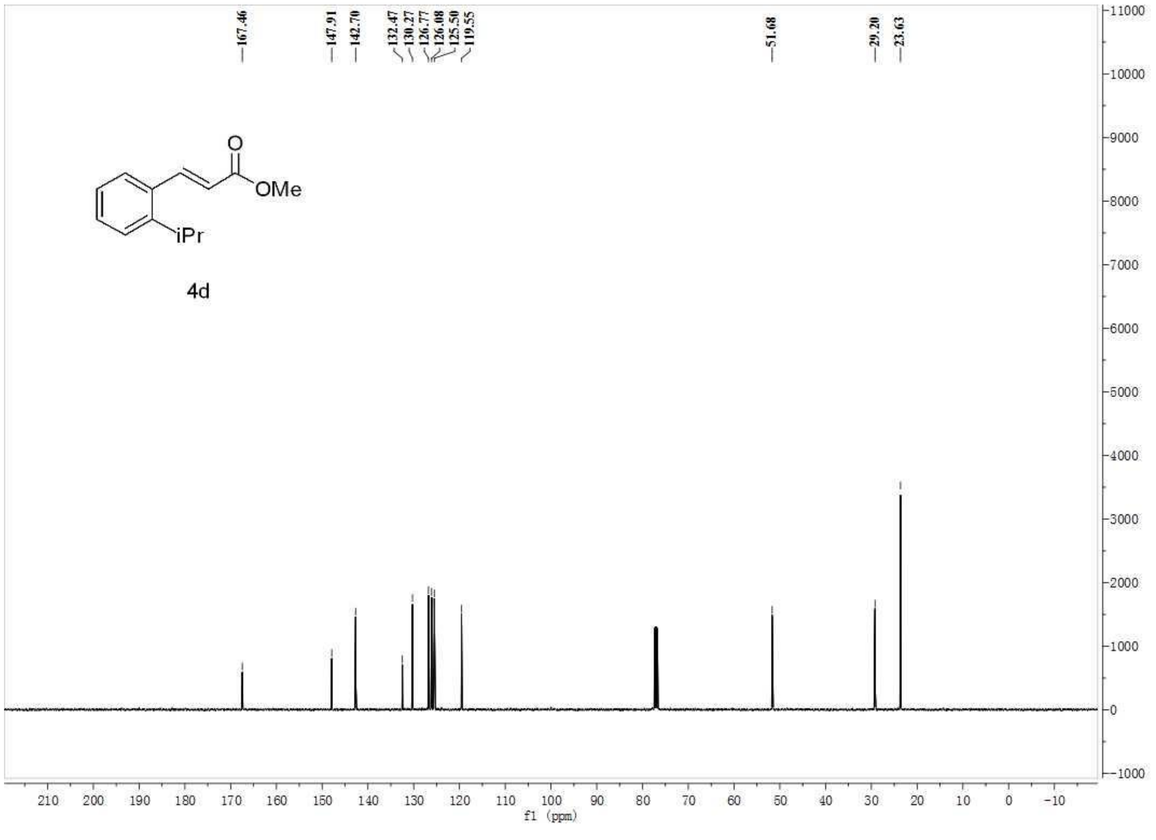
167.46
147.91
142.70
132.47
130.27
126.77
126.08
125.50
119.55
51.68
29.20
23.63
OMe
iPr
4d
f1 (ppm)

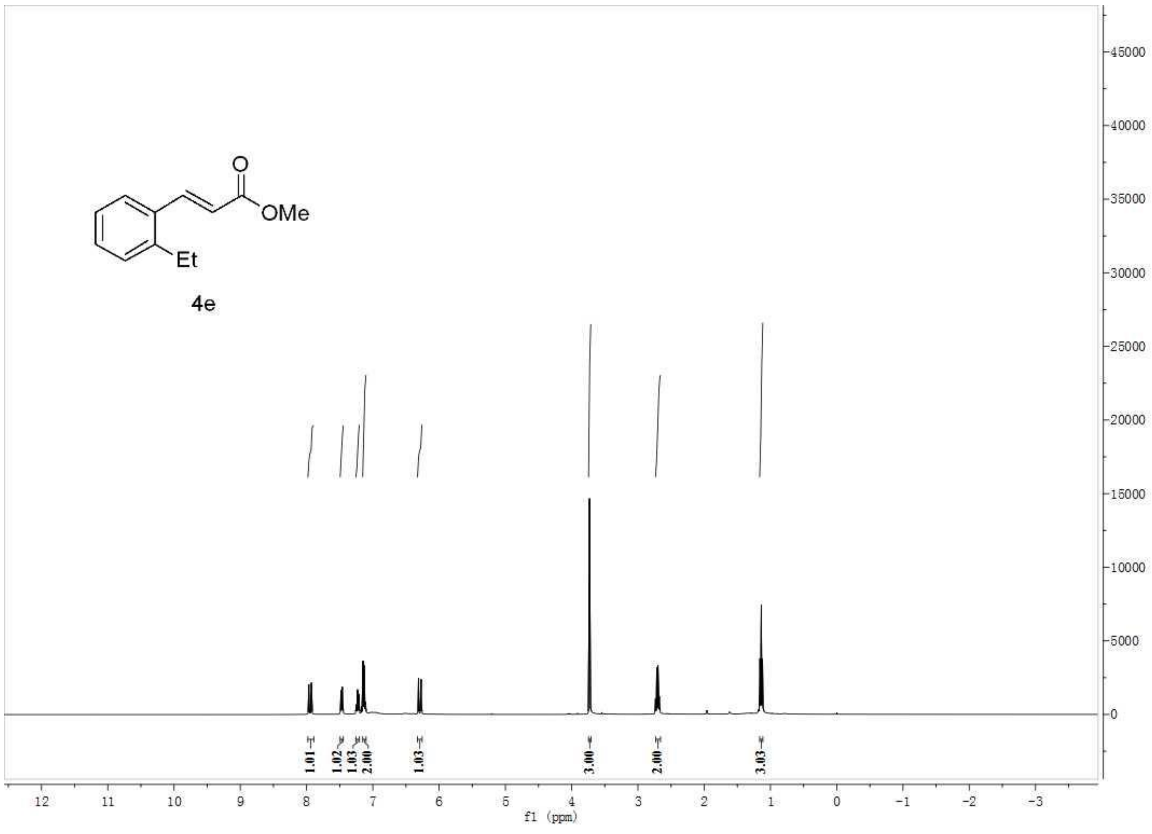
OMe
Et
4e
1.01
1.02
1.03
2.00
1.03
3.00
2.00
3.03
f1 (ppm)

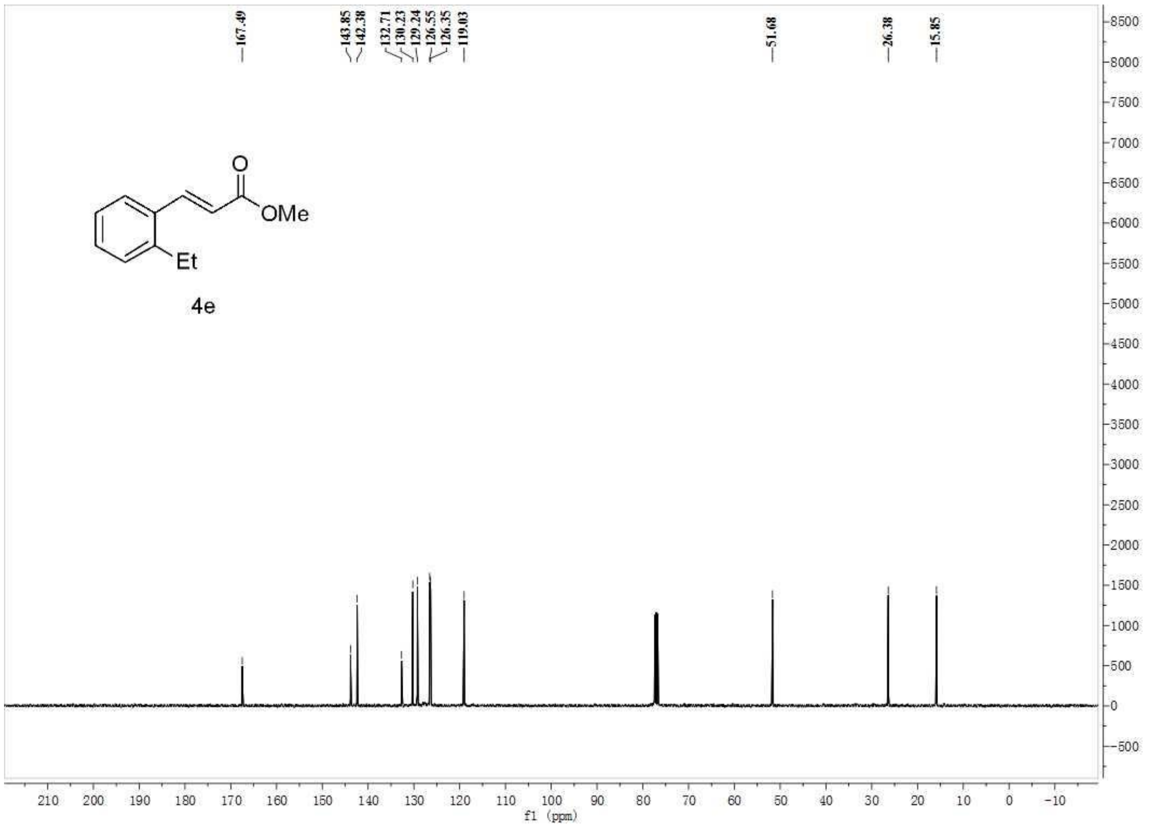
167.49
143.85
142.38
132.71
130.23
129.24
126.55
126.35
119.03
51.68
26.38
15.85
OMe
Et
4e
f1 (ppm)

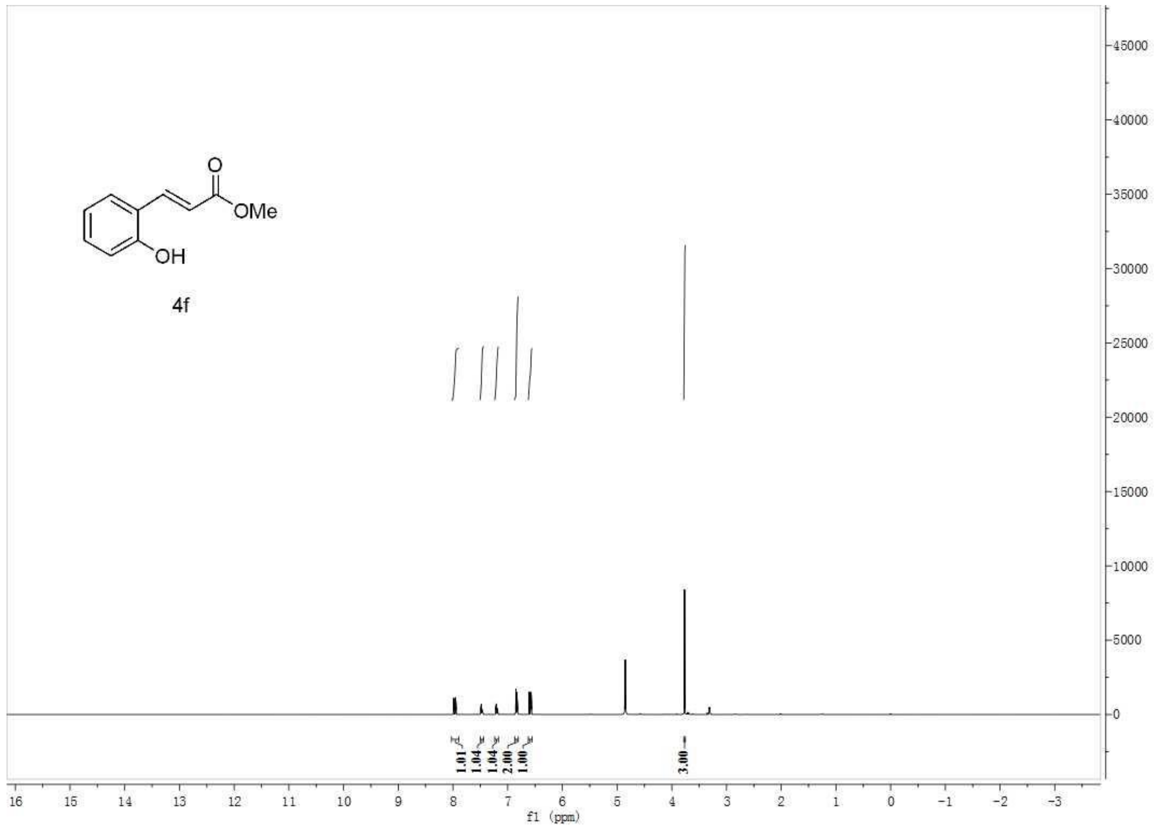
O
OMe
OH
4f
1.01
1.04
1.04
2.00
1.00
3.00
f1 (ppm)

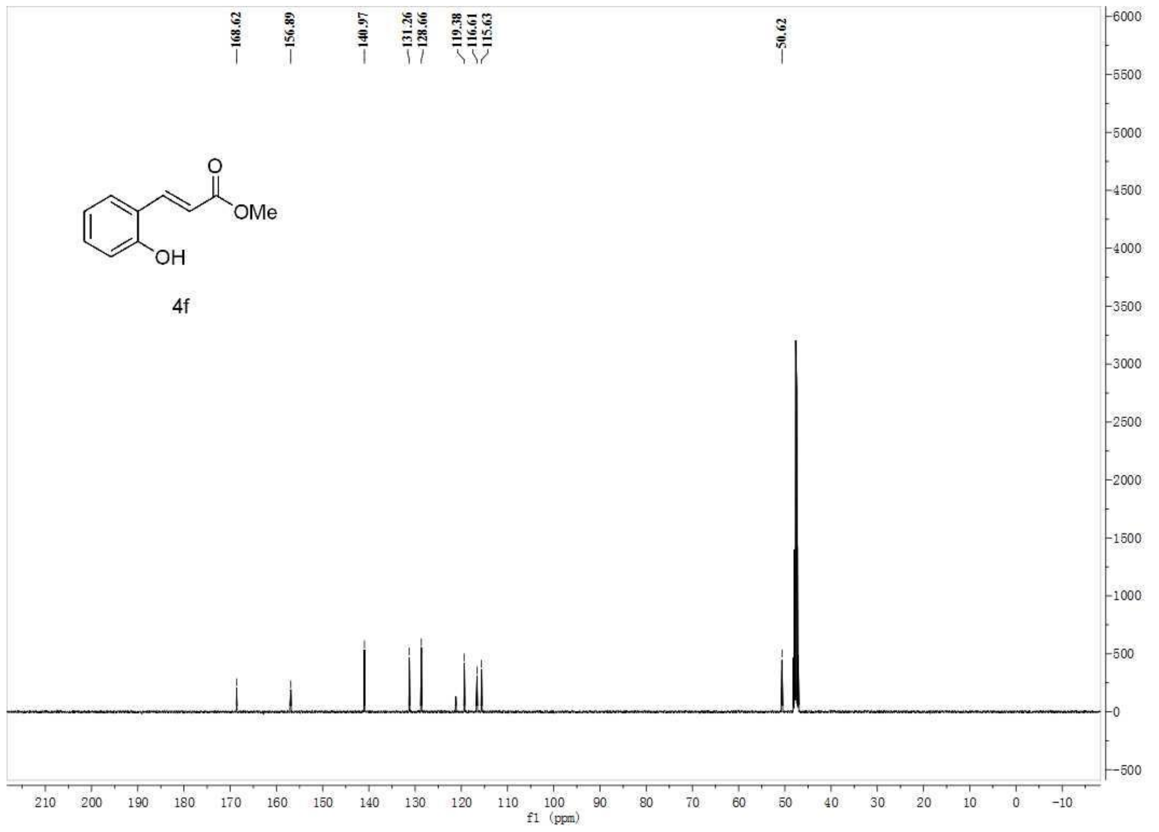
168.62
156.89
140.97
131.26
128.66
119.38
116.61
115.63
50.62
O
OMe
OH
4f
f1 (ppm)

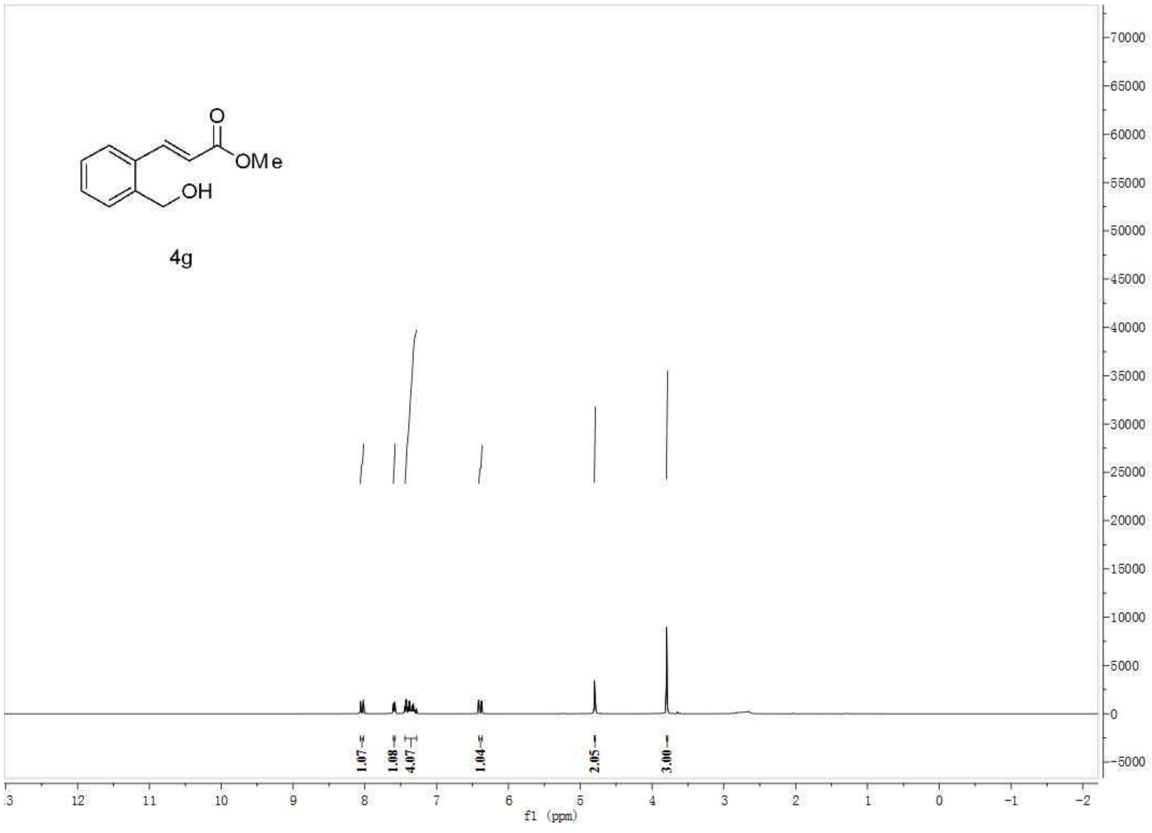

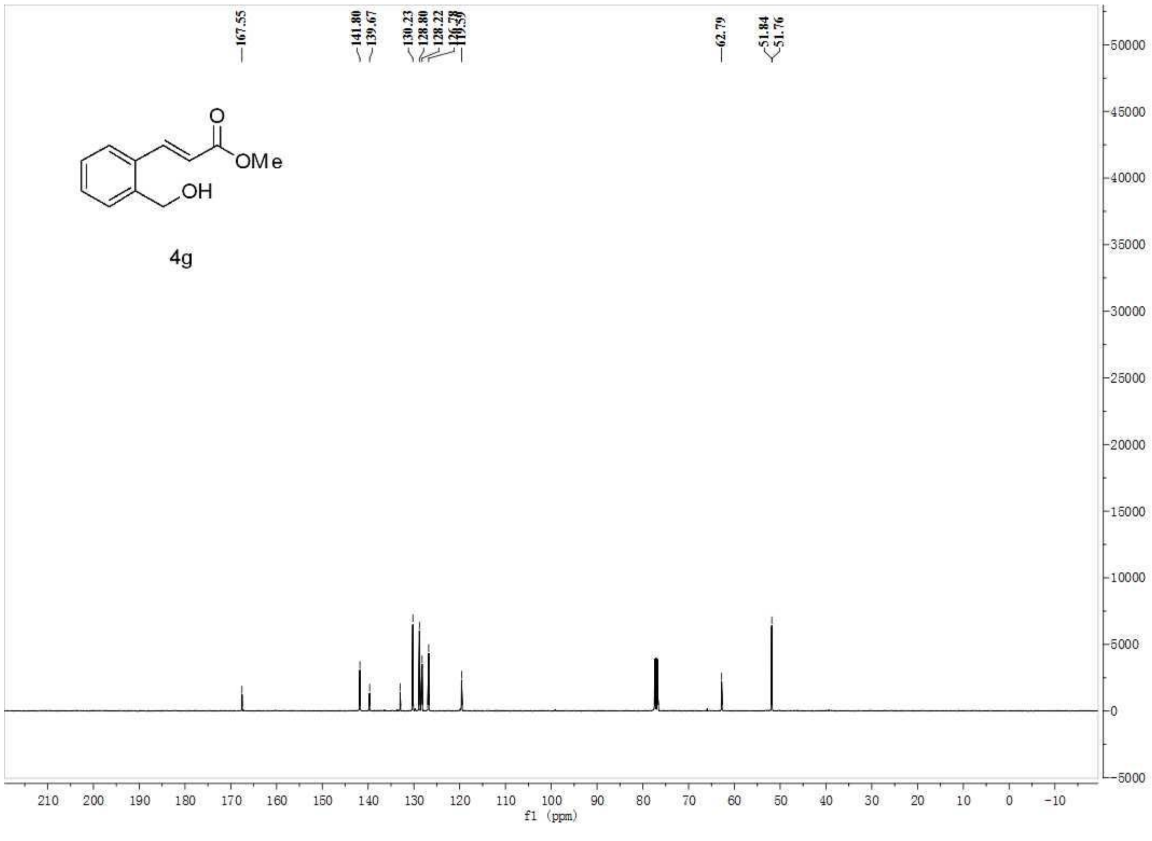

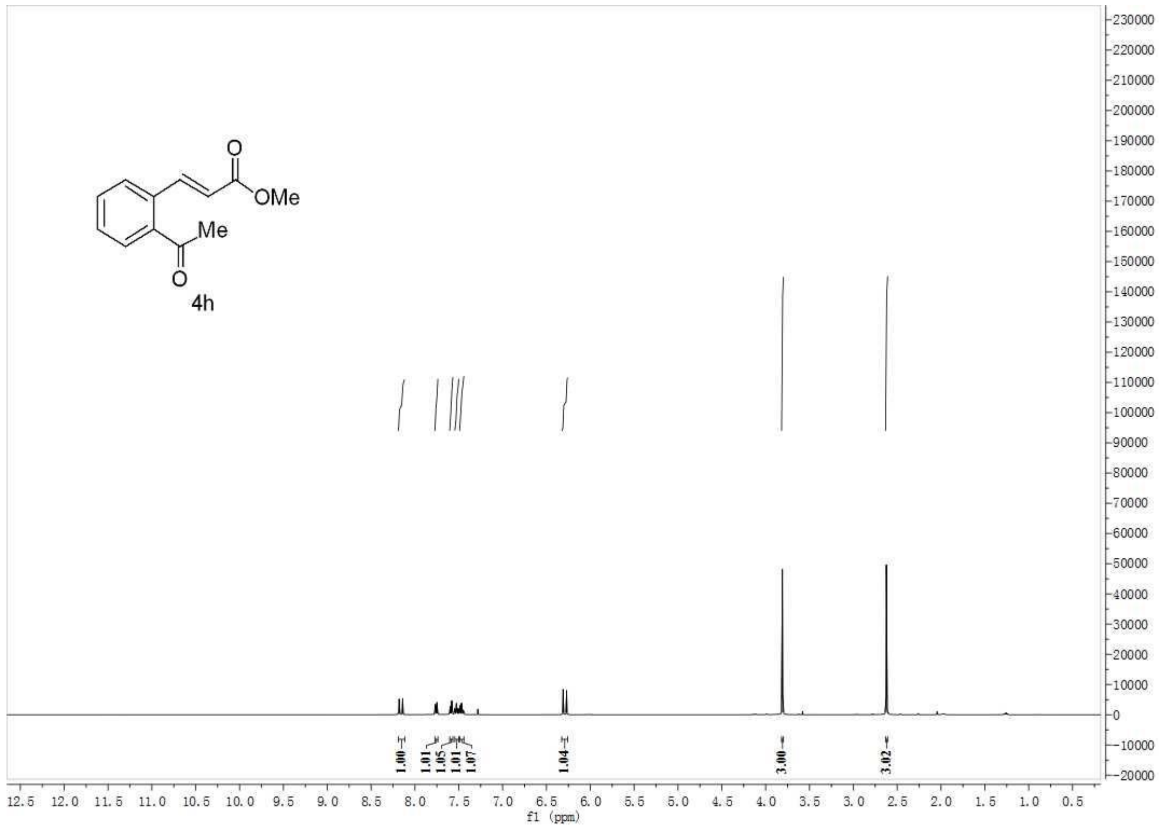

OMe
Me
4h
1.00
1.01
1.05
1.01
1.07
1.04
3.00
3.02
f1 (ppm)

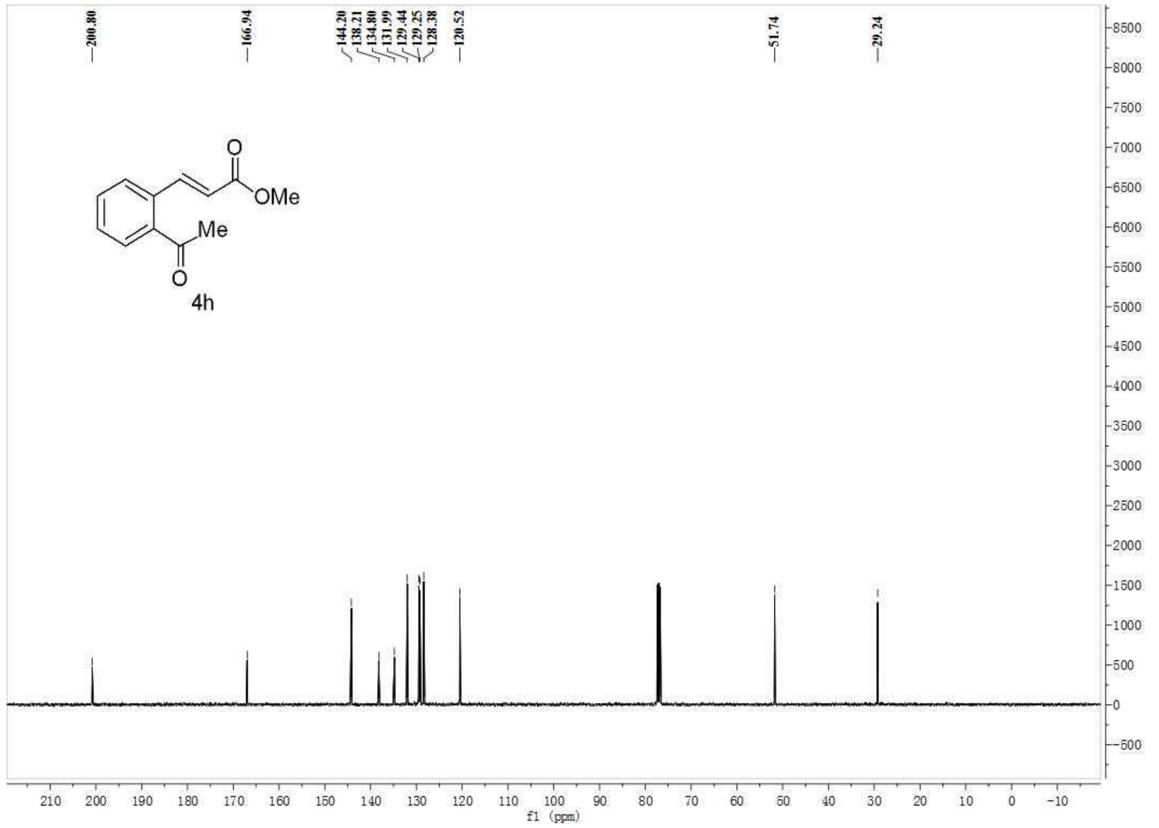

200.80
166.94
144.20
138.21
134.80
131.99
129.44
129.25
128.38
120.52
51.74
29.24
OMe
Me
4h
f1 (ppm)

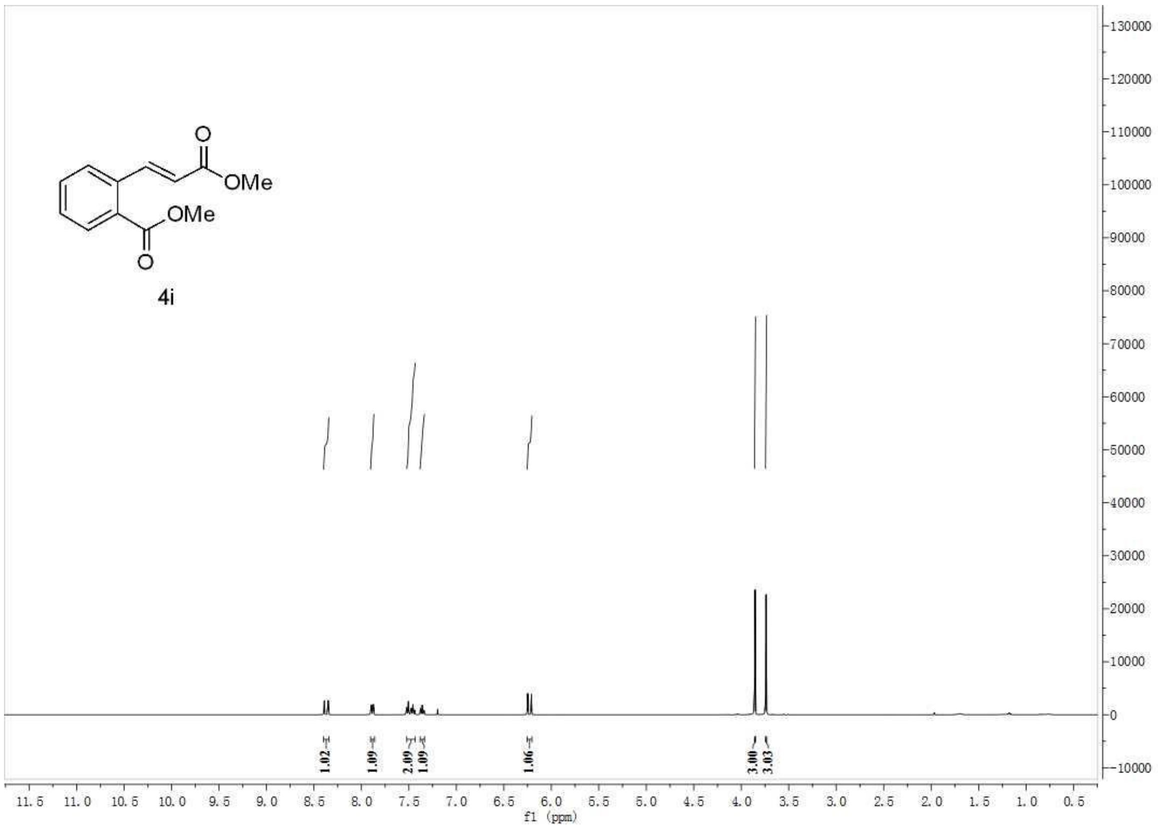
OMe
OMe
4i
1.02
1.09
2.09
1.09
1.06
3.00
3.03
f1 (ppm)

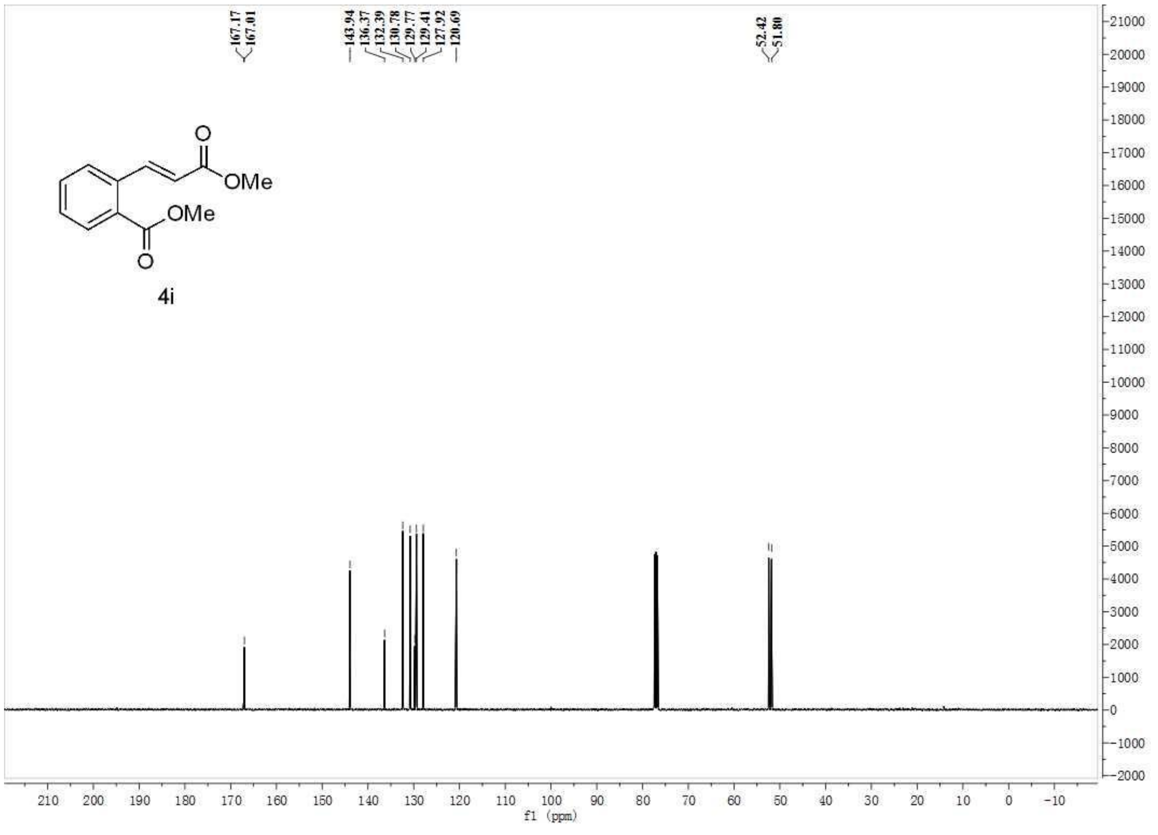
167.17
167.01
143.94
136.37
132.39
130.78
129.77
129.41
127.92
120.69
52.42
51.80
OMe
OMe
4i
f1 (ppm)

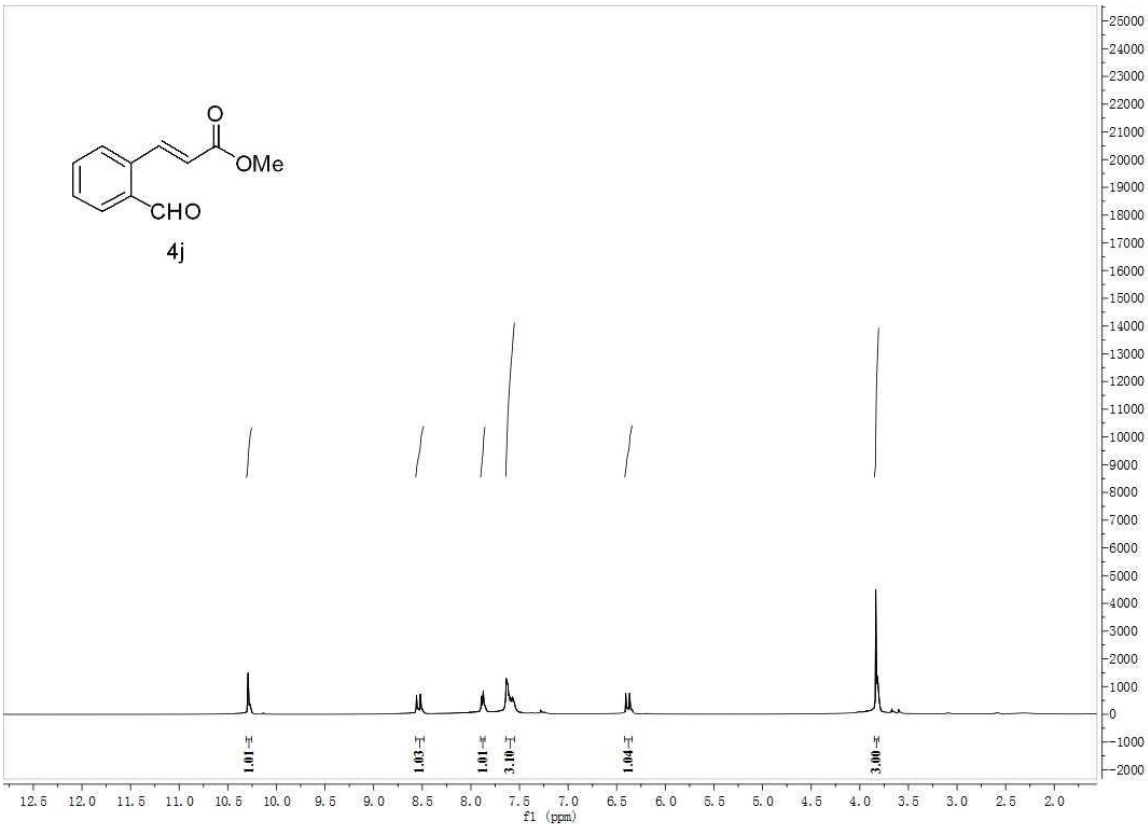
O
OMe
CHO
4j
1.01
1.03
1.01
3.10
1.04
3.00
f1 (ppm)

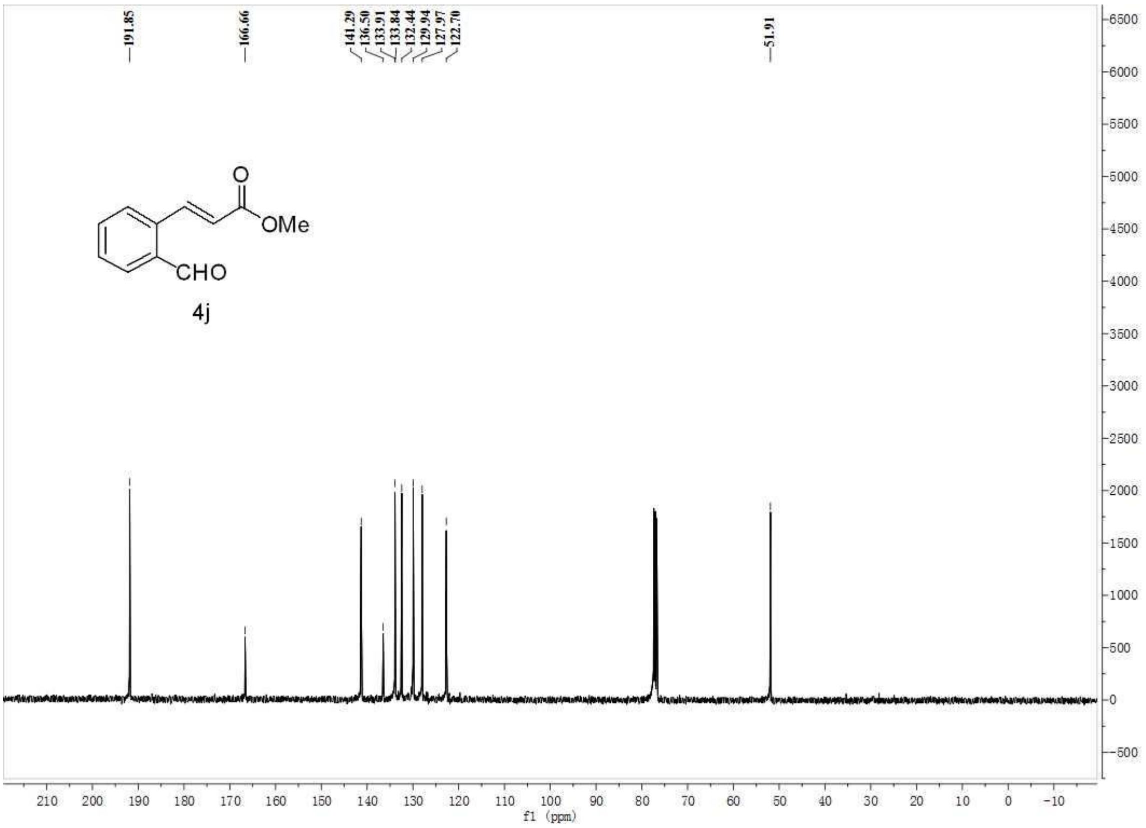
191.85
166.66
141.29
136.50
133.91
133.84
132.44
129.94
127.97
122.70
51.91
O
OMe
CHO
4j
f1 (ppm)

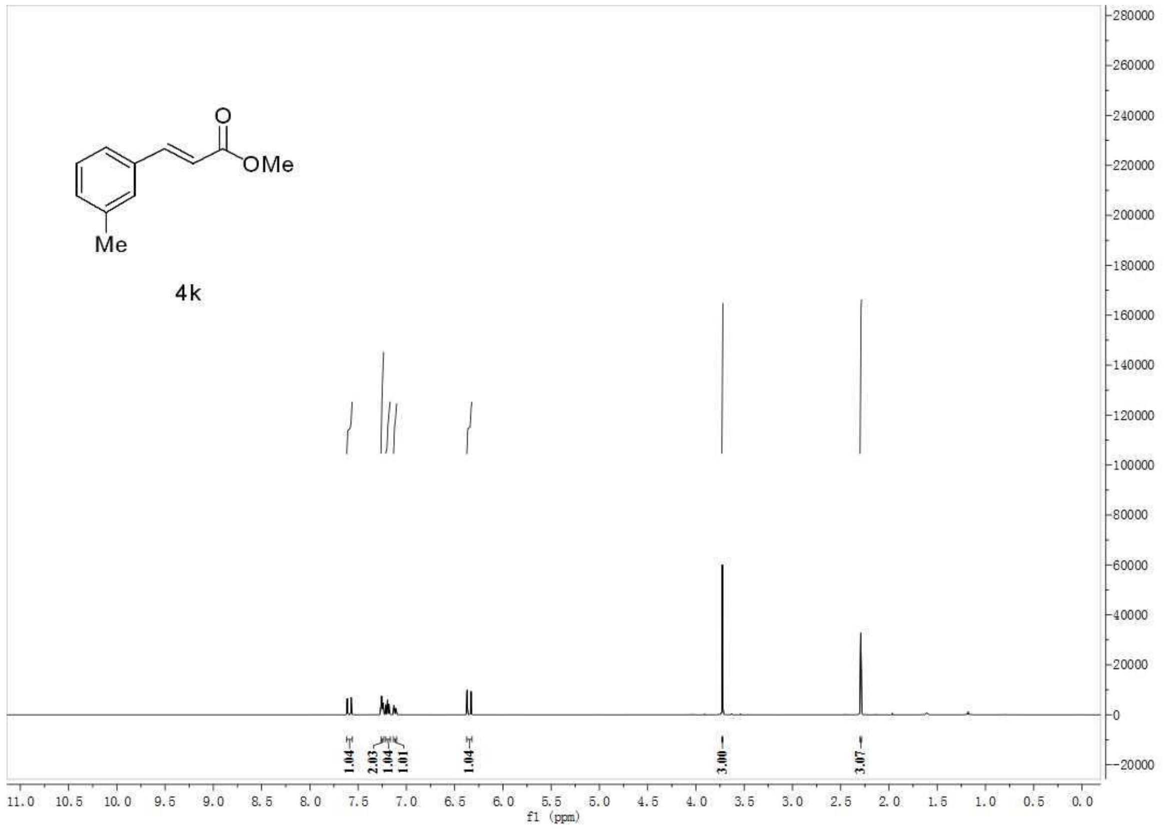

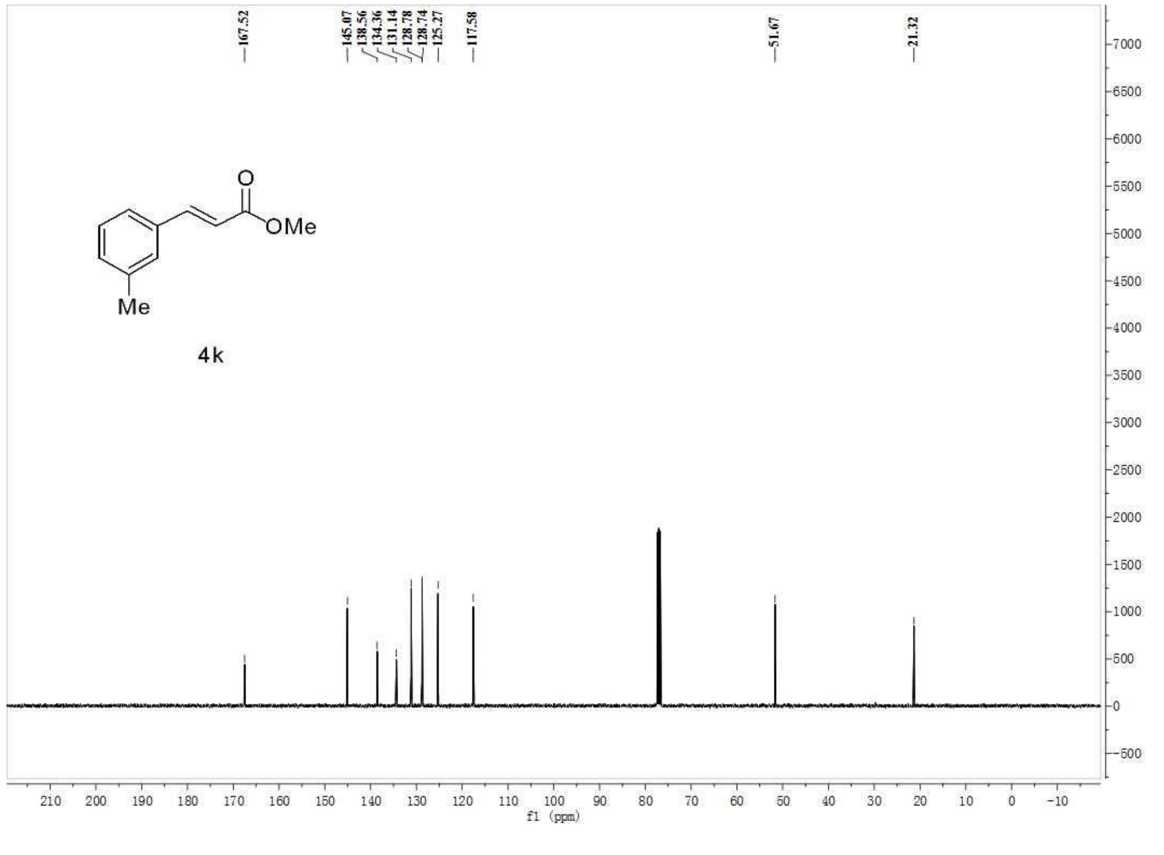

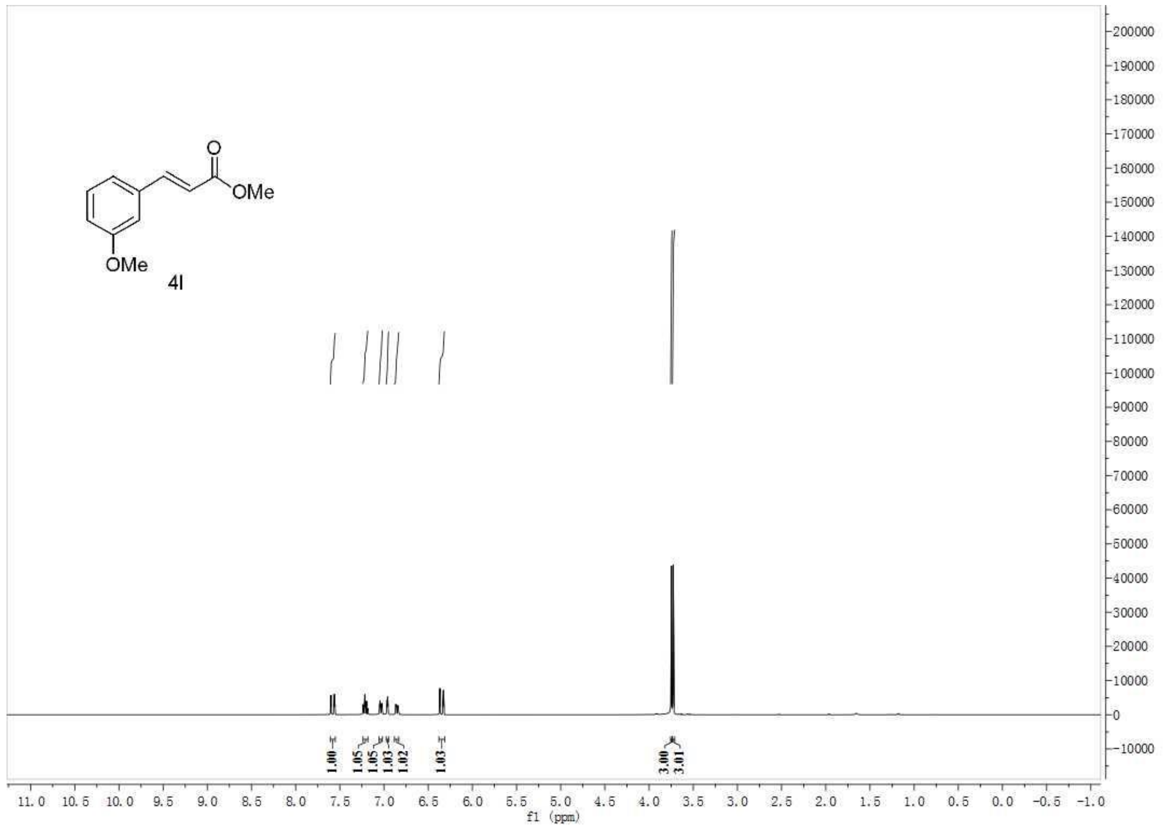
OMe
OMe
4l
1.00
1.05
1.05
1.03
1.02
1.03
3.00
3.01
f1 (ppm)

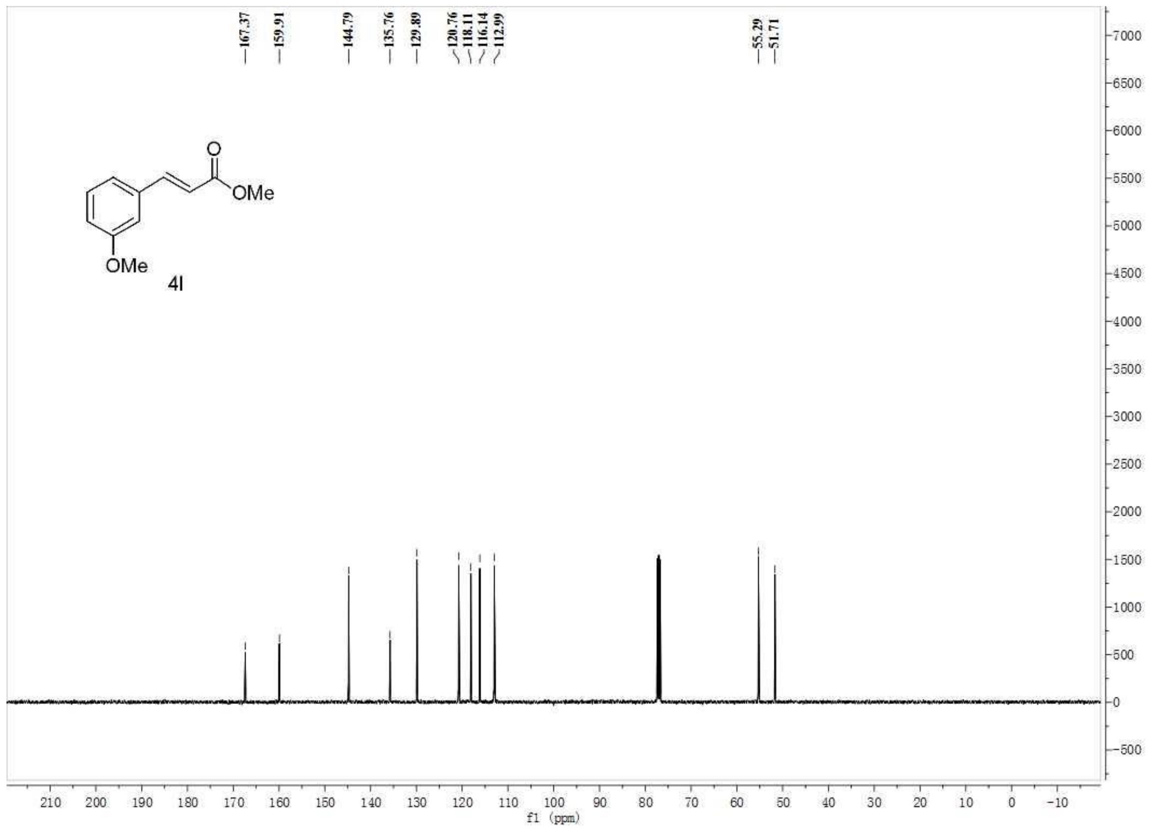
167.37
159.91
144.79
135.76
129.89
120.76
118.11
116.14
112.99
55.29
51.71
OMe
OMe
4l
f1 (ppm)

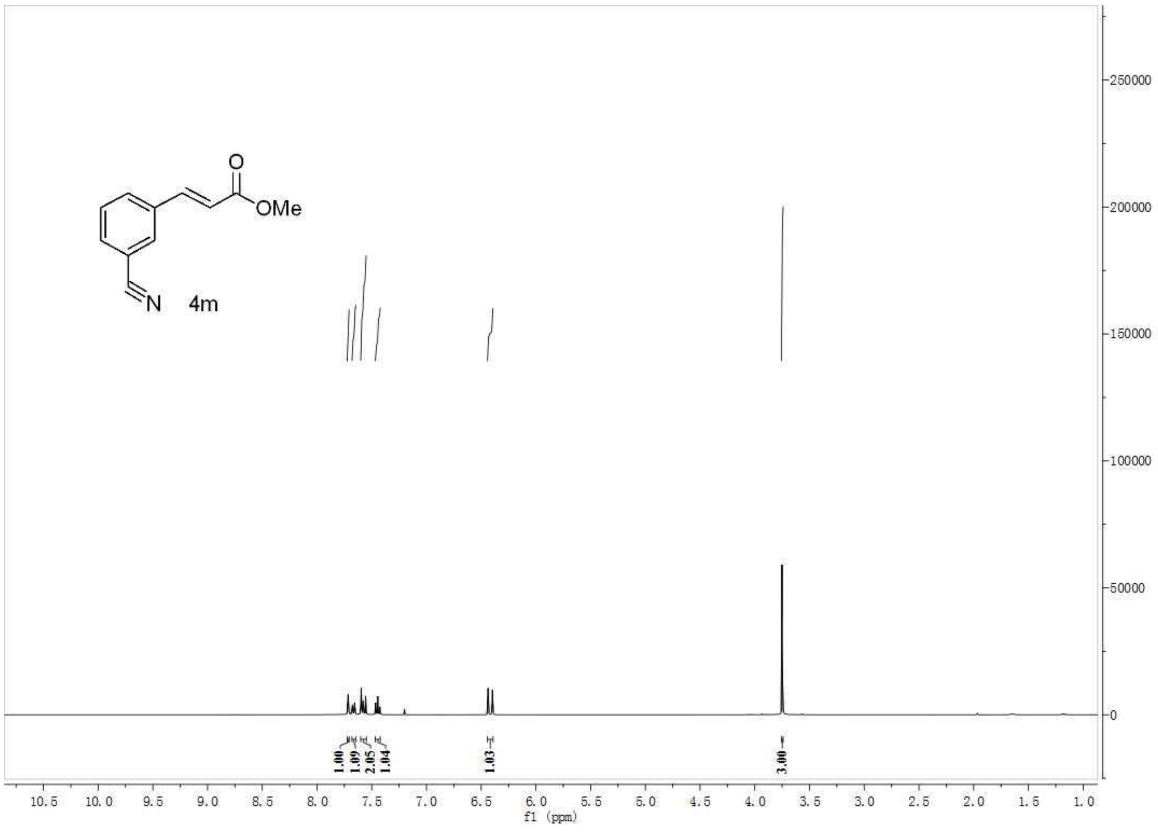
OMe
O
N
4m
1.00
1.09
2.05
1.04
1.03
3.00
f1 (ppm)

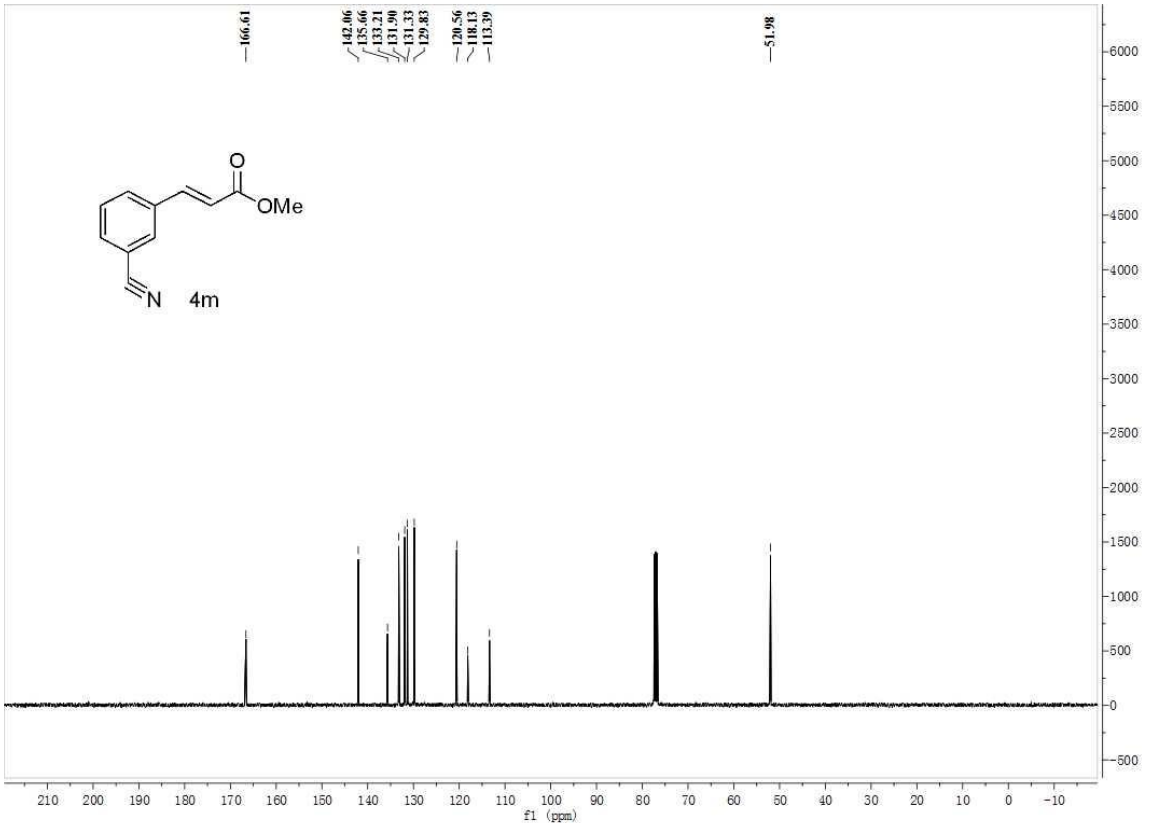
166.61
142.06
135.66
133.21
131.90
131.33
129.83
120.56
118.13
113.39
51.98
OMe
O
N
4m
f1 (ppm)

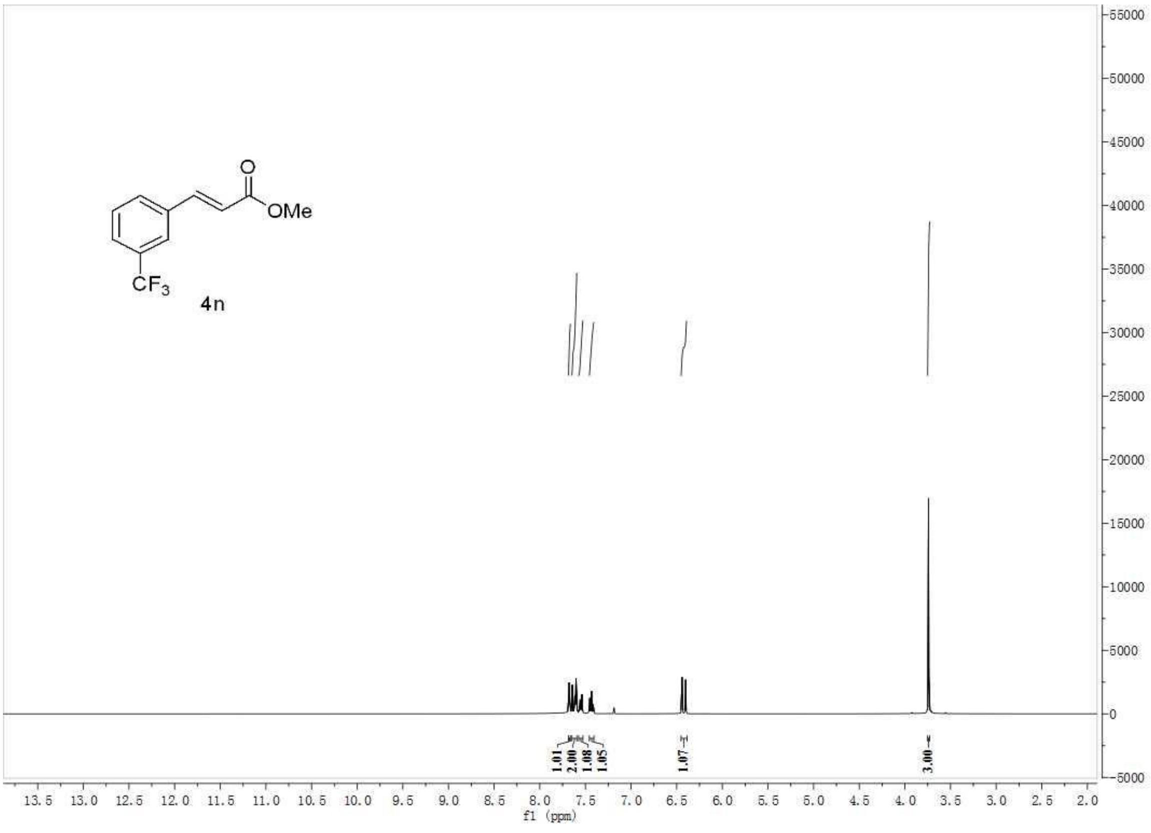
OMe
CF_3
4n
1.01
2.00
1.08
1.05
1.07
3.00
f1 (ppm)

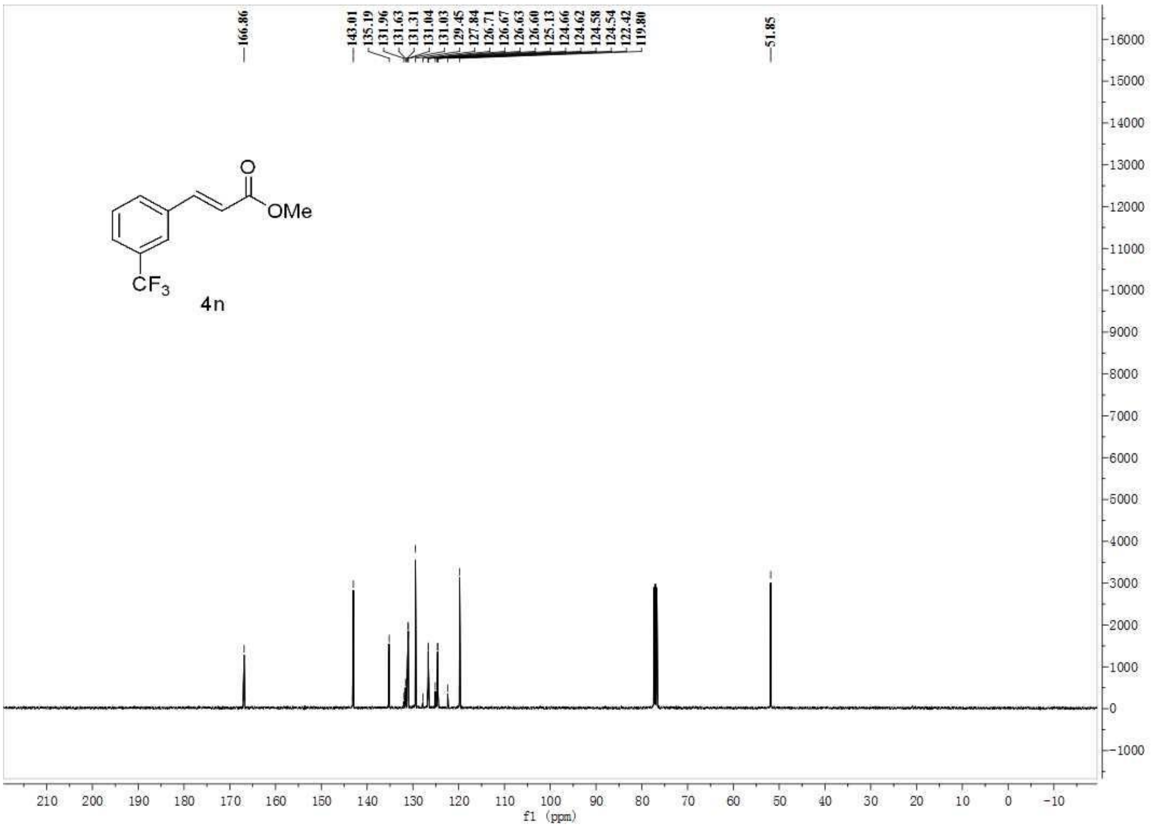
166.86
143.01
135.19
131.96
131.63
131.31
131.04
131.03
129.45
127.84
126.71
126.67
126.63
126.60
125.13
124.66
124.62
124.58
124.54
122.42
119.80
51.85
OMe
CF_3
4n
f1 (ppm)

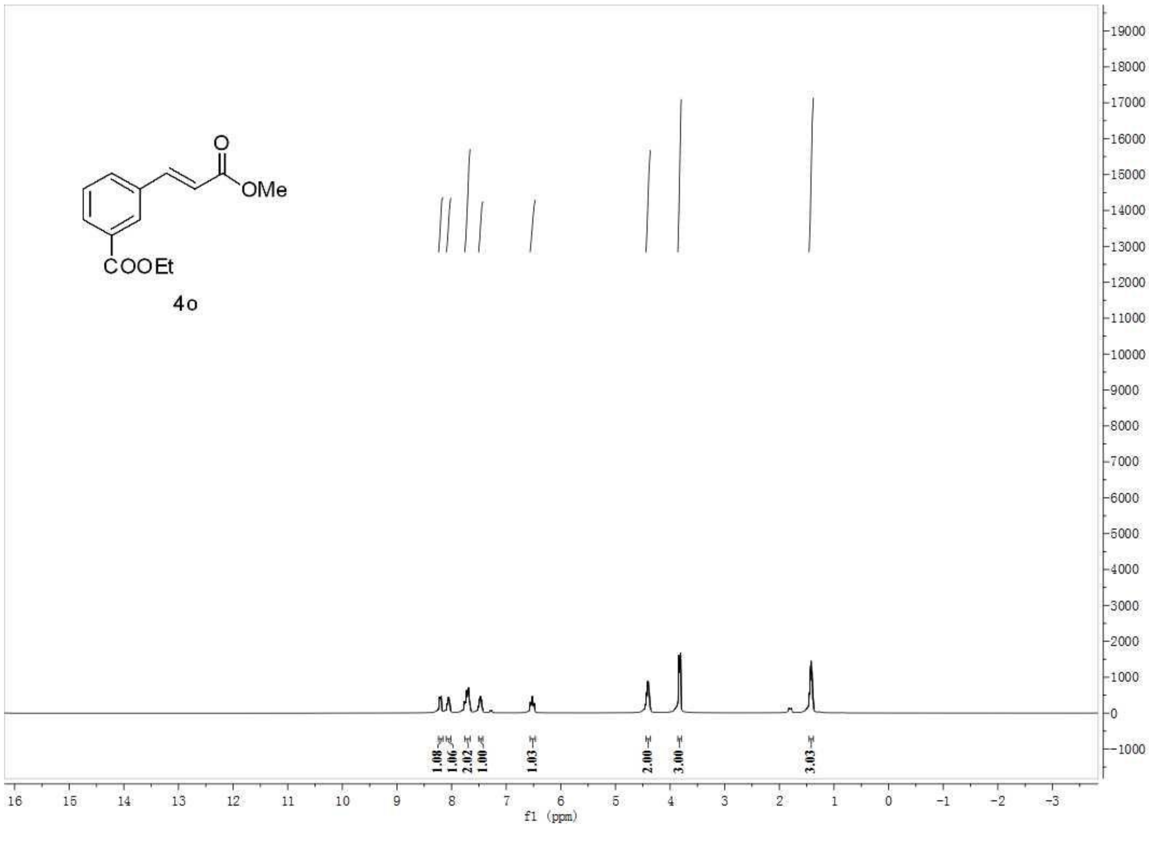
OMe
COOEt
4o
1.08
1.06
2.02
1.00
1.03
2.00
3.00
3.03
f1 (ppm)

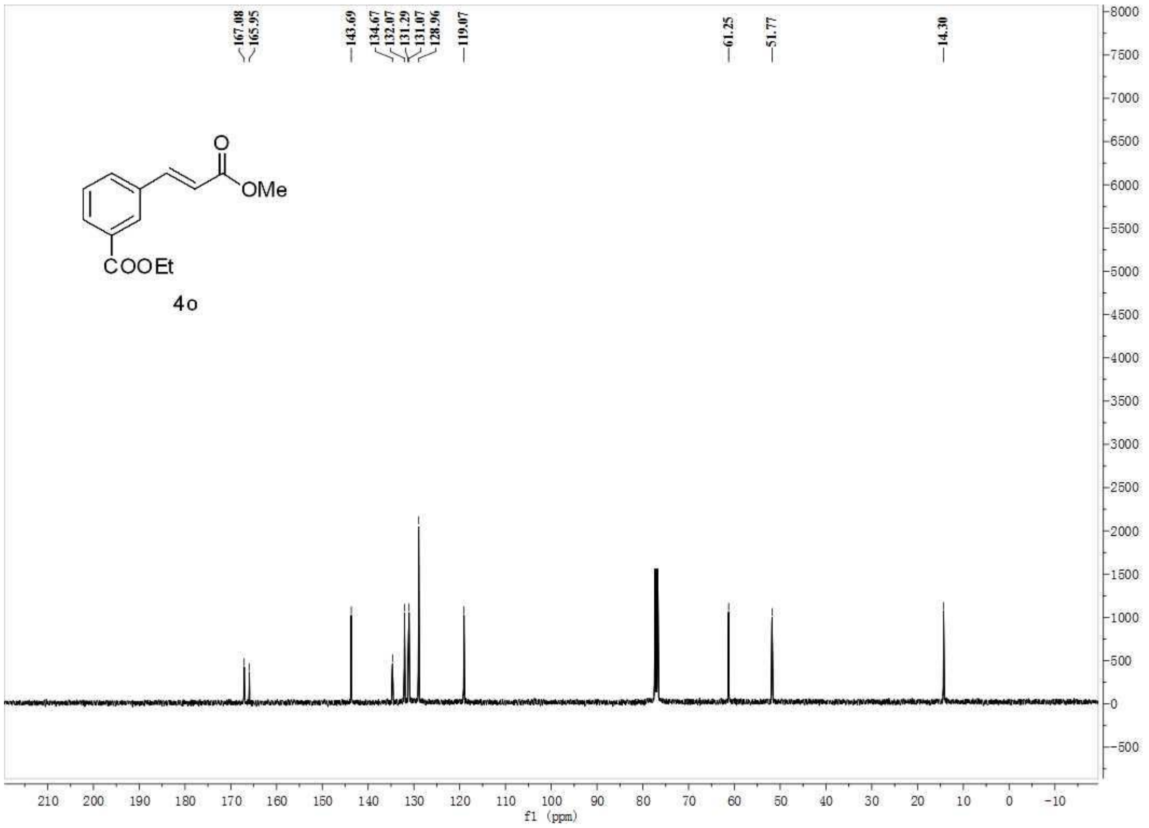
167.08
165.95
143.69
134.67
132.07
131.29
131.07
128.96
119.07
61.25
51.77
14.30
OMe
COOEt
4o
f1 (ppm)

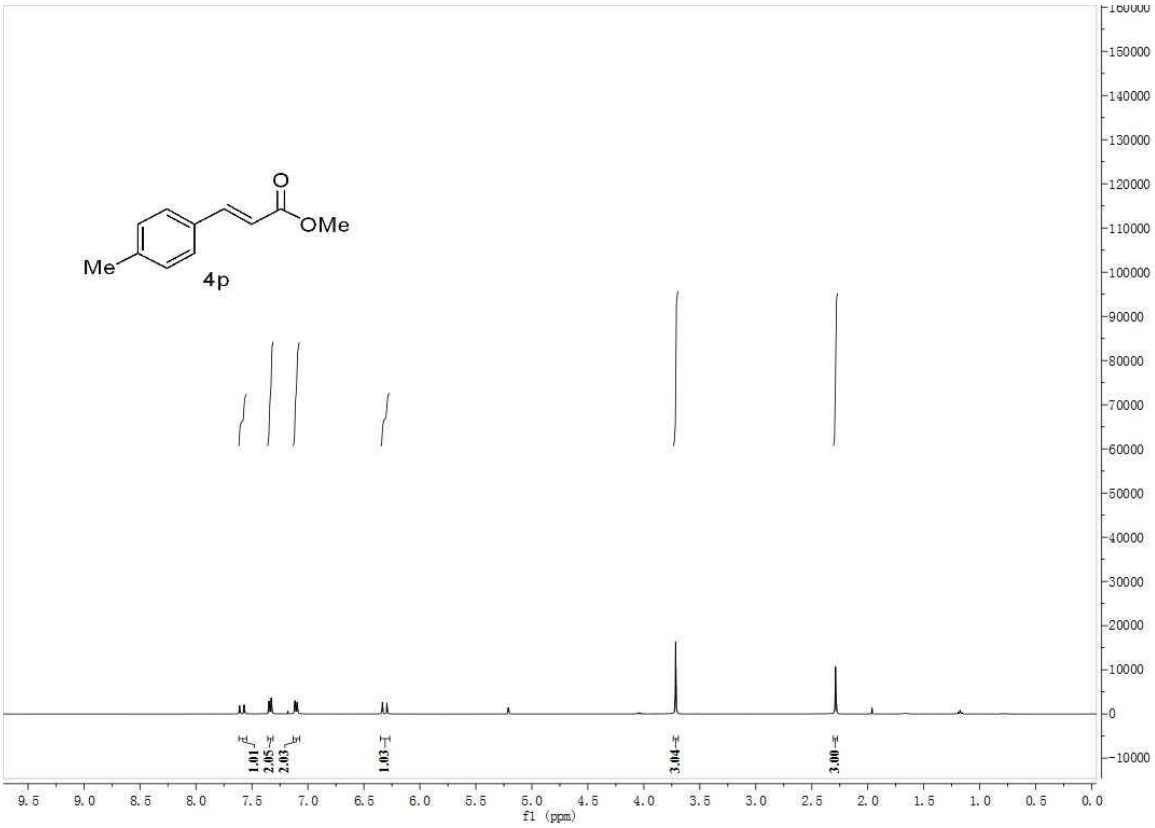

OMe
Me
4p
1.01
2.05
2.03
1.03
3.04
3.00
f1 (ppm)

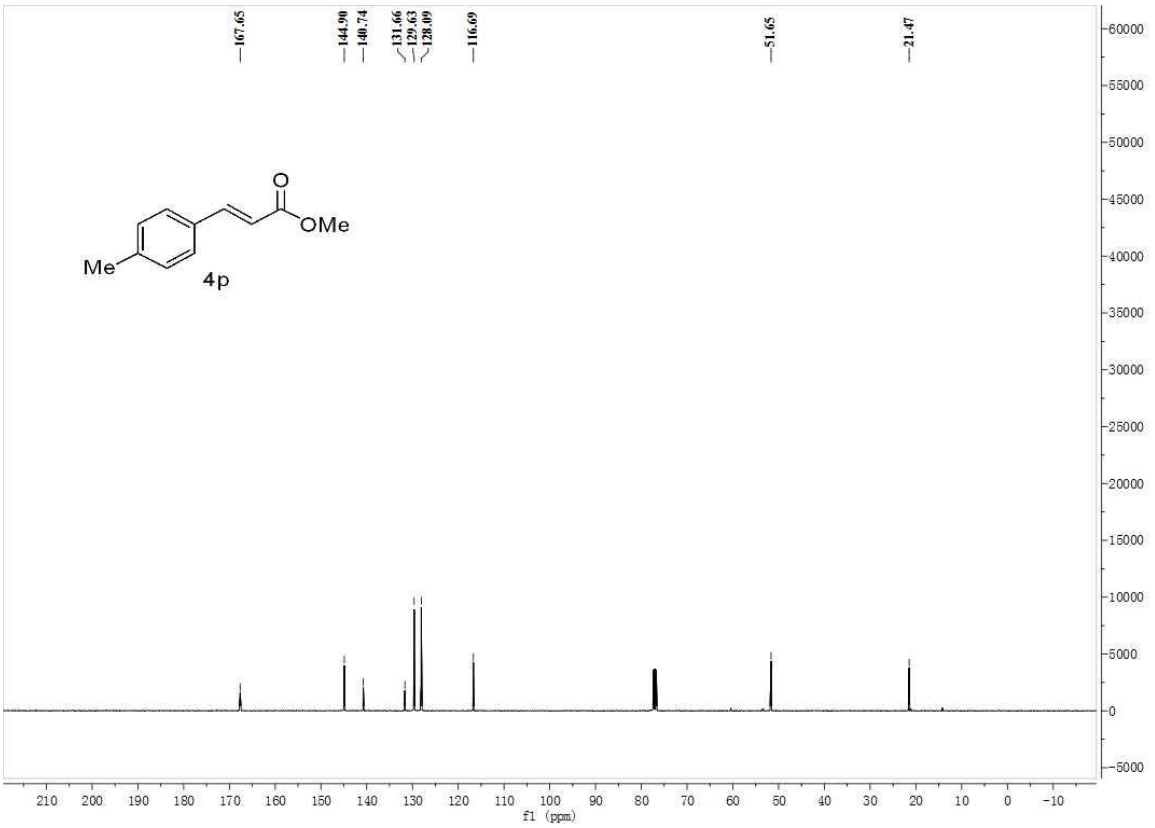

167.65
144.90
140.74
131.66
129.63
128.09
116.69
51.65
21.47
OMe
Me
4p
f1 (ppm)

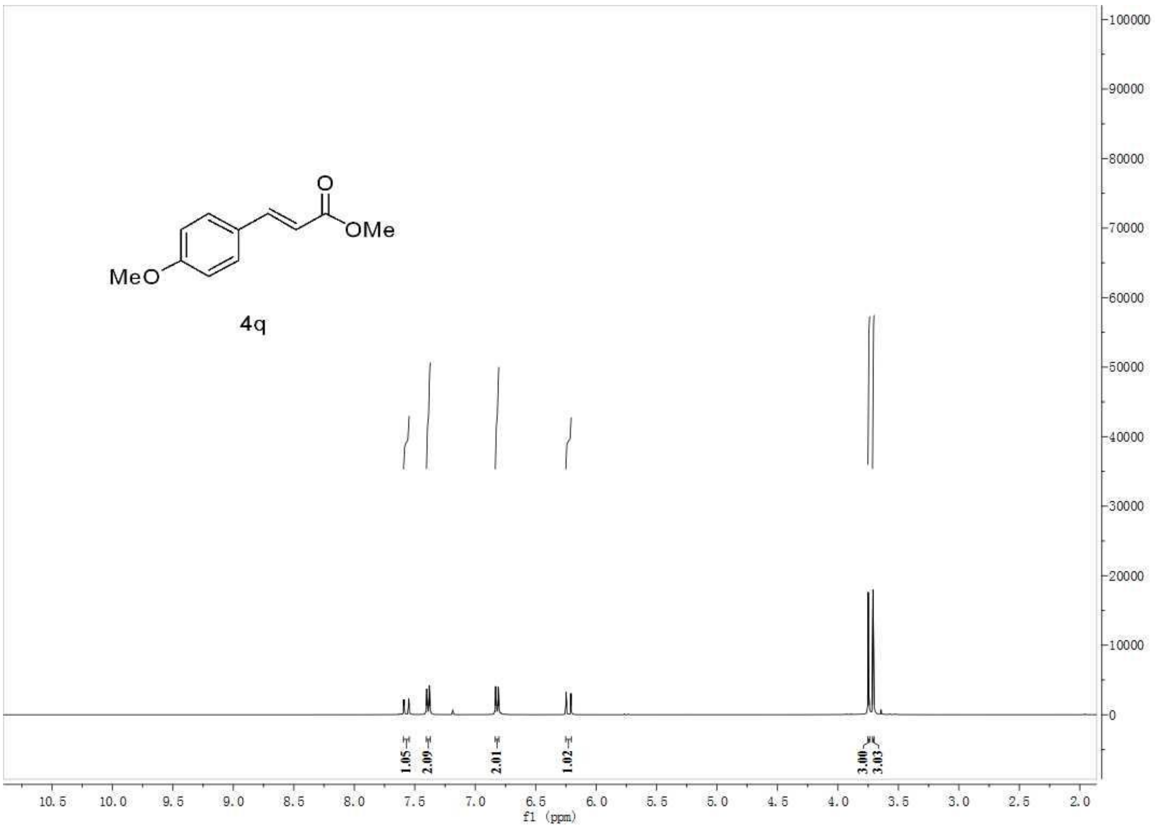

MeO
OMe
4q
1.05
2.09
2.01
1.02
3.00
3.03
f1 (ppm)

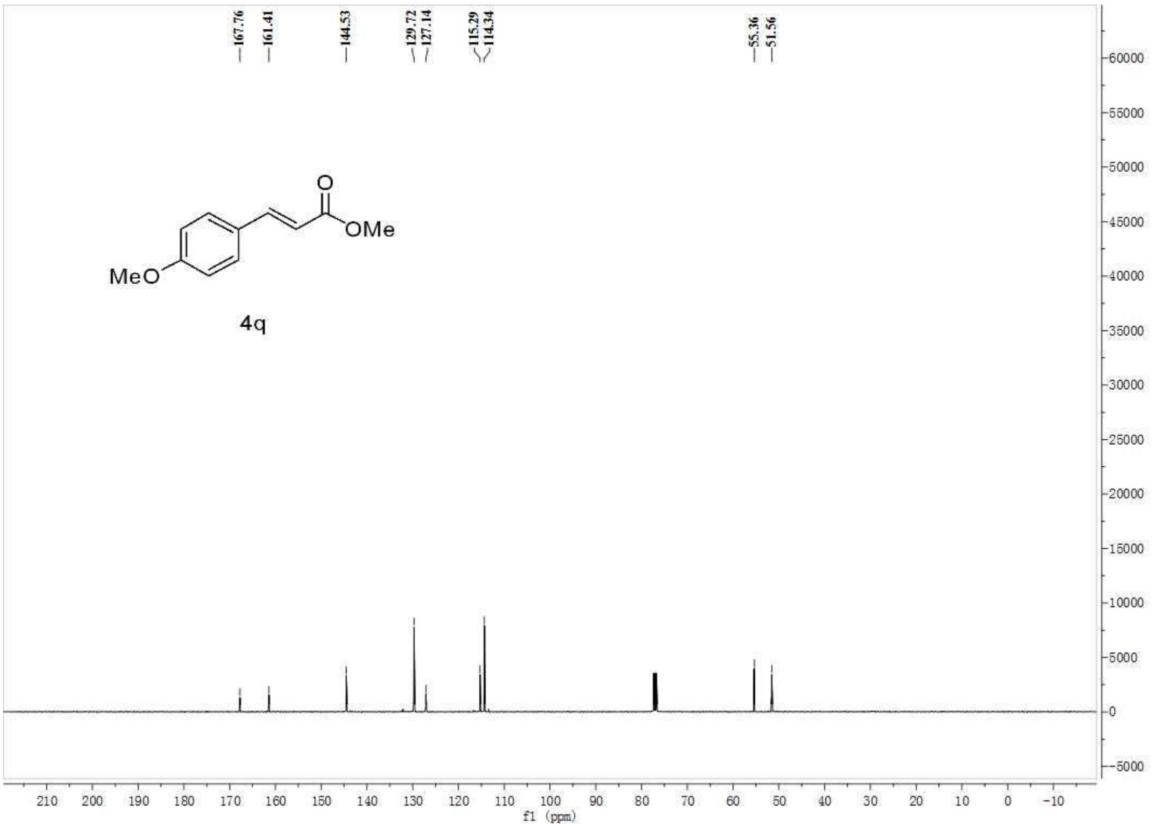

167.76
161.41
144.53
129.72
127.14
115.29
114.34
55.36
51.56
MeO
OMe
4q
f1 (ppm)

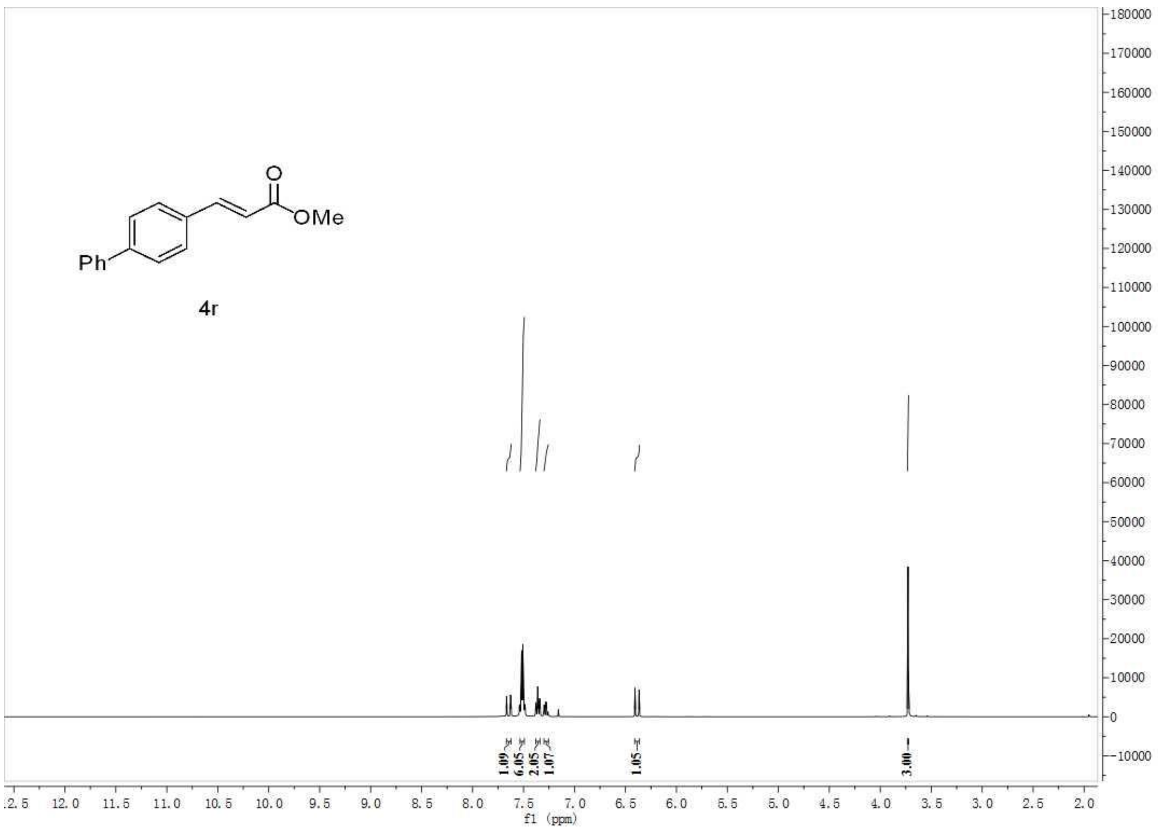

OMe
Ph
4r
1.09
6.05
2.05
1.07
1.05
3.00
f1 (ppm)

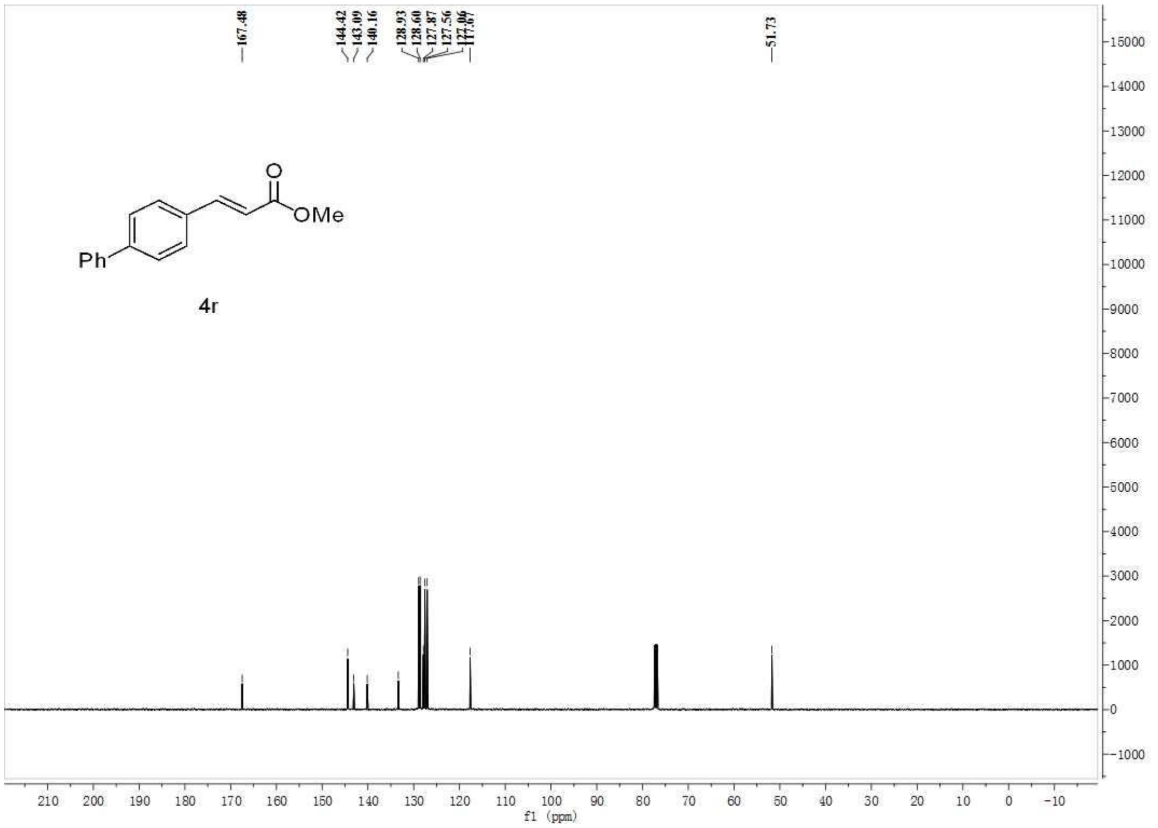

167.48
144.42
143.09
140.16
128.93
128.60
127.87
127.56
51.73
OMe
Ph
4r
f1 (ppm)

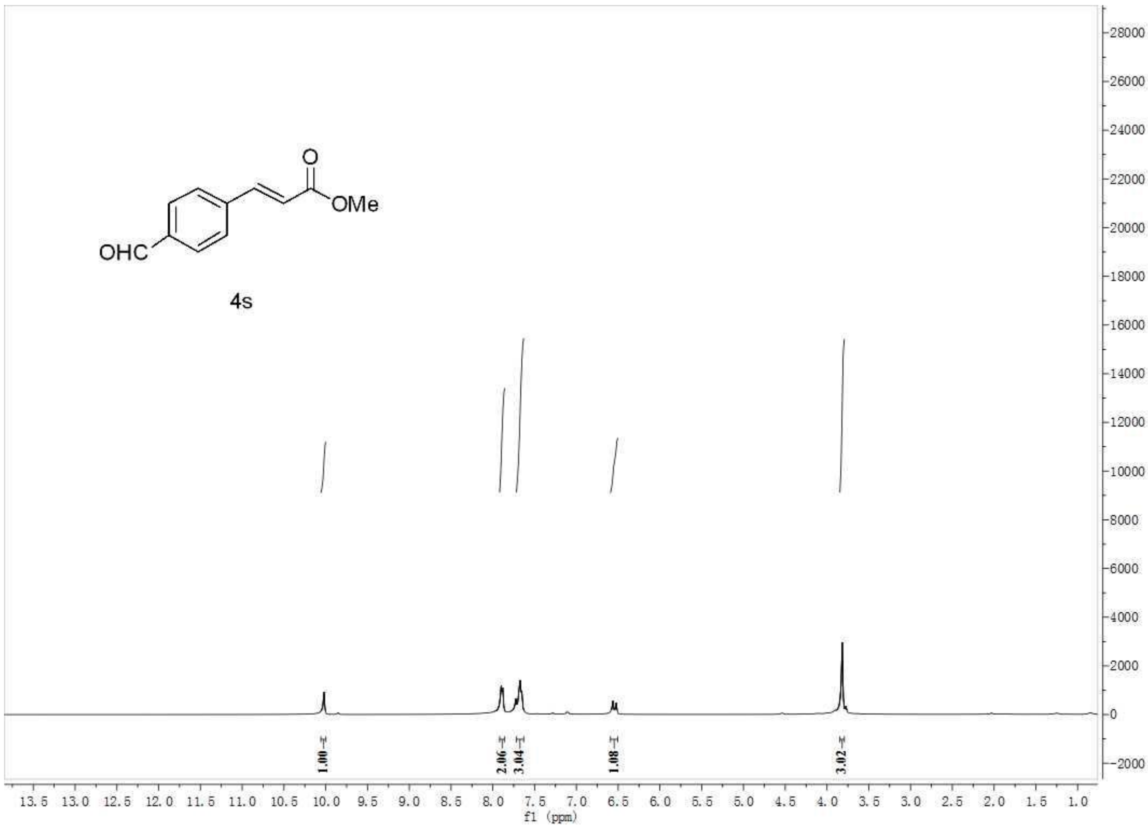

O
OMe
OHC
4s
1.00
2.06
3.04
1.08
3.02
f1 (ppm)

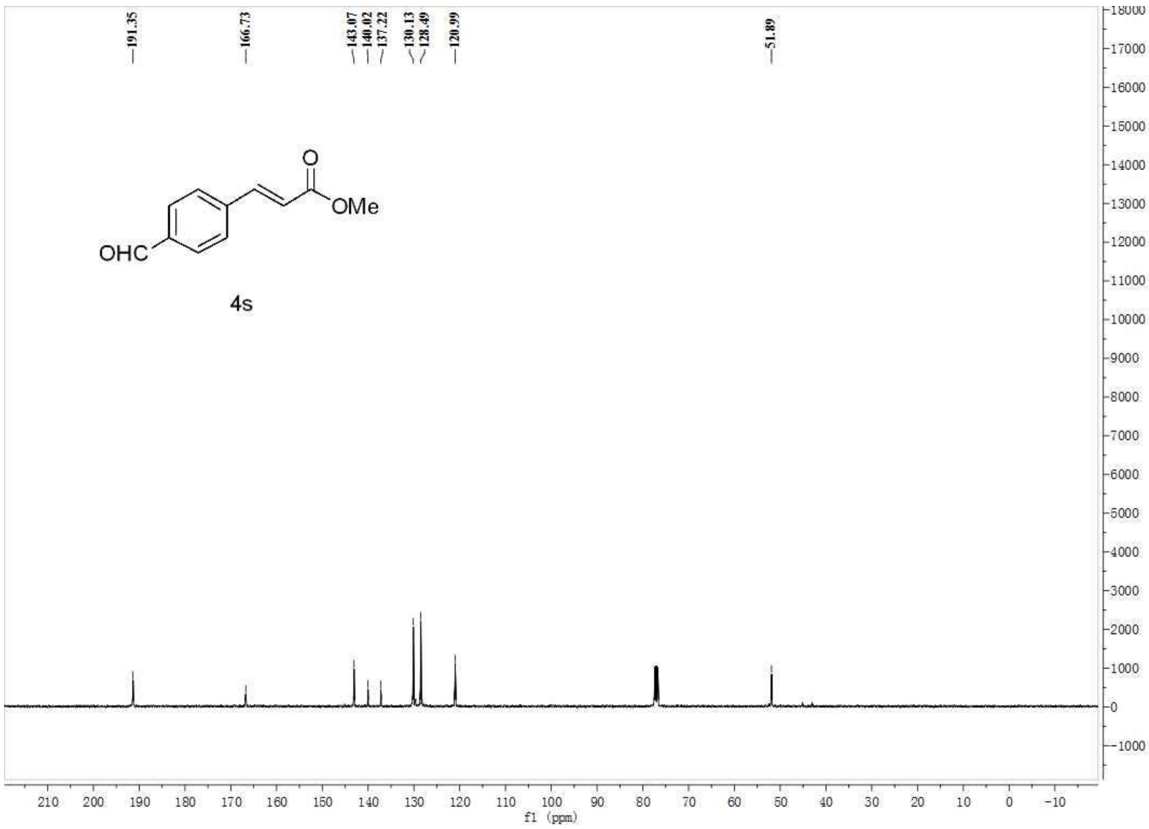

191.35
166.73
143.07
140.02
137.22
130.13
128.49
120.99
51.89
O
OMe
OHC
4s
f1 (ppm)

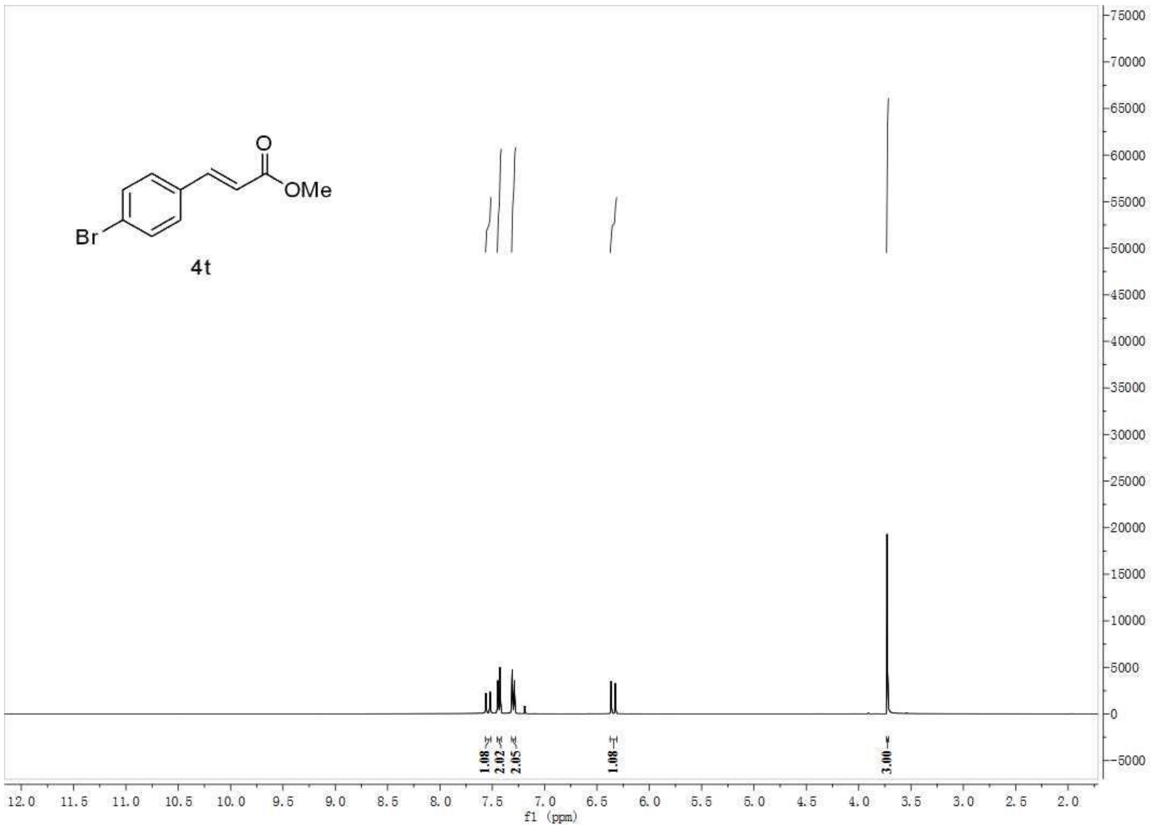
O
OMe
Br
4t
1.08
2.02
2.05
1.08
3.00
f1 (ppm)

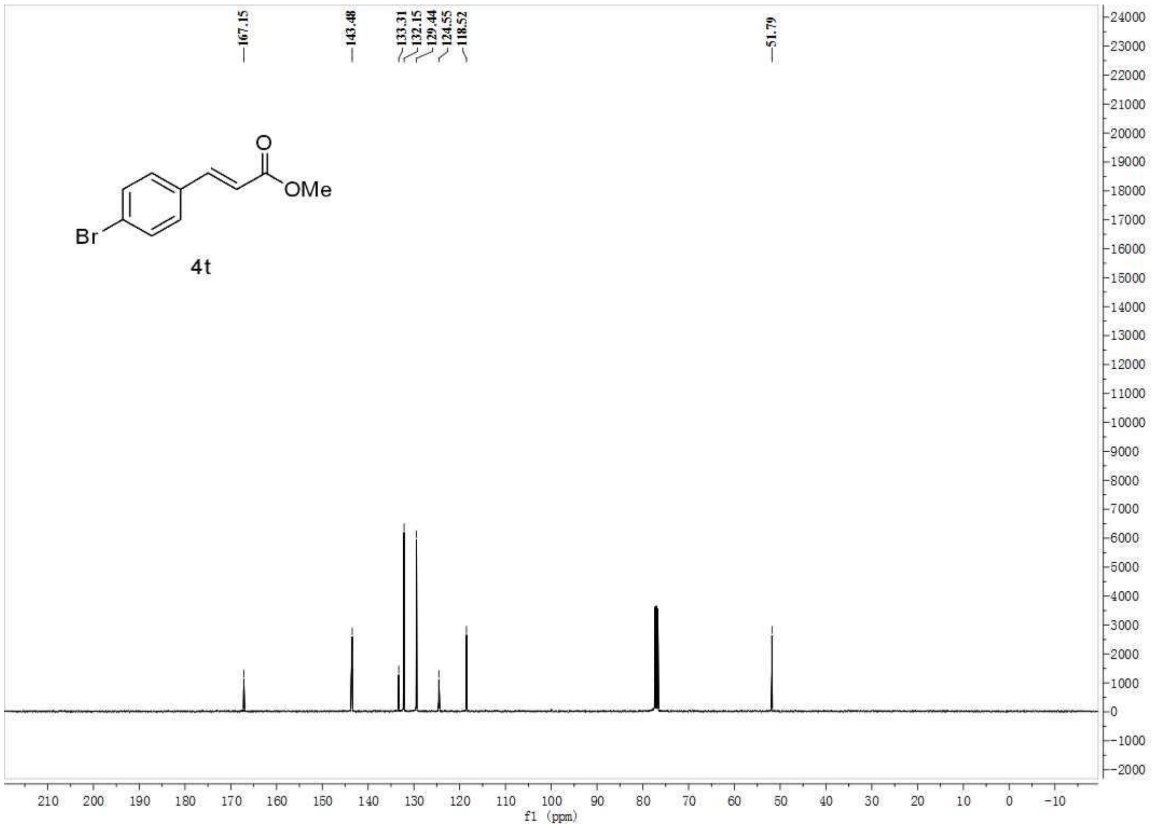
167.15
143.48
133.31
132.15
129.44
124.55
118.52
51.79
O
OMe
Br
4t
f1 (ppm)

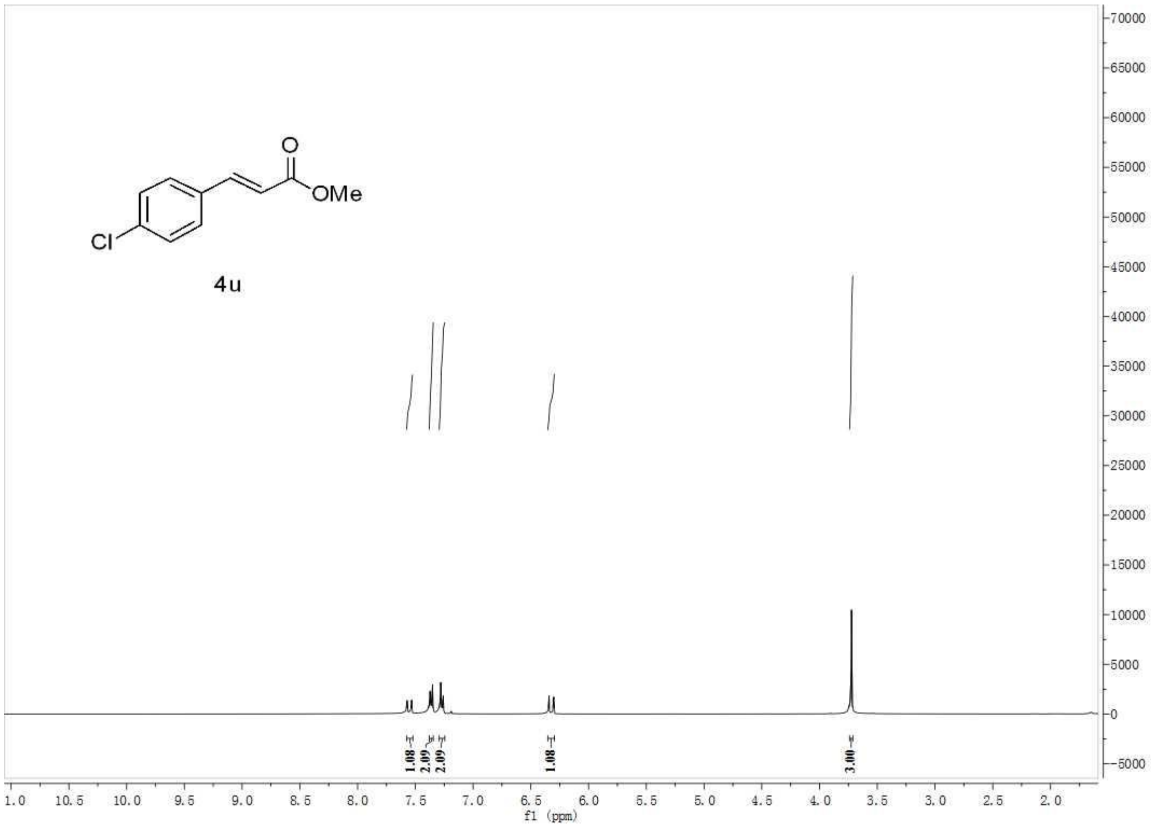
OMe
Cl
4u
1.08
2.09
2.09
1.08
3.00
f1 (ppm)

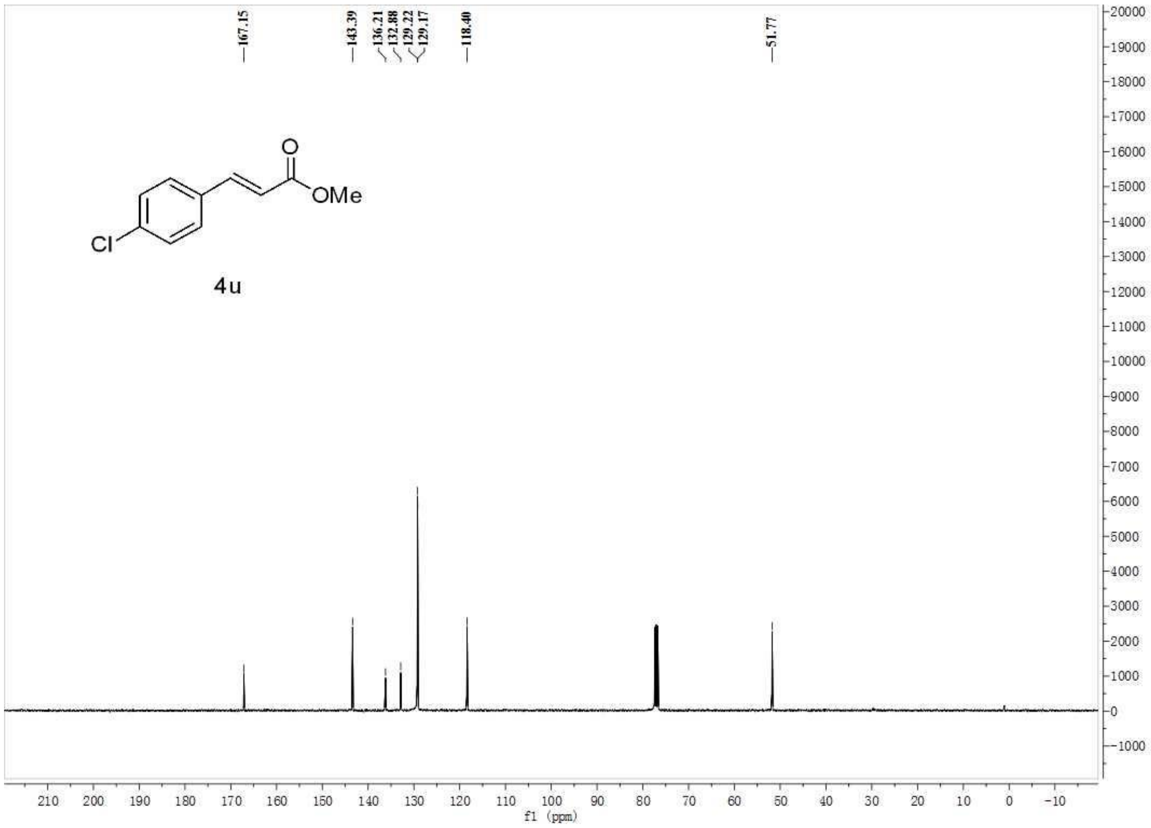
167.15
143.39
136.21
132.88
129.22
129.17
118.40
51.77
OMe
Cl
4u
f1 (ppm)

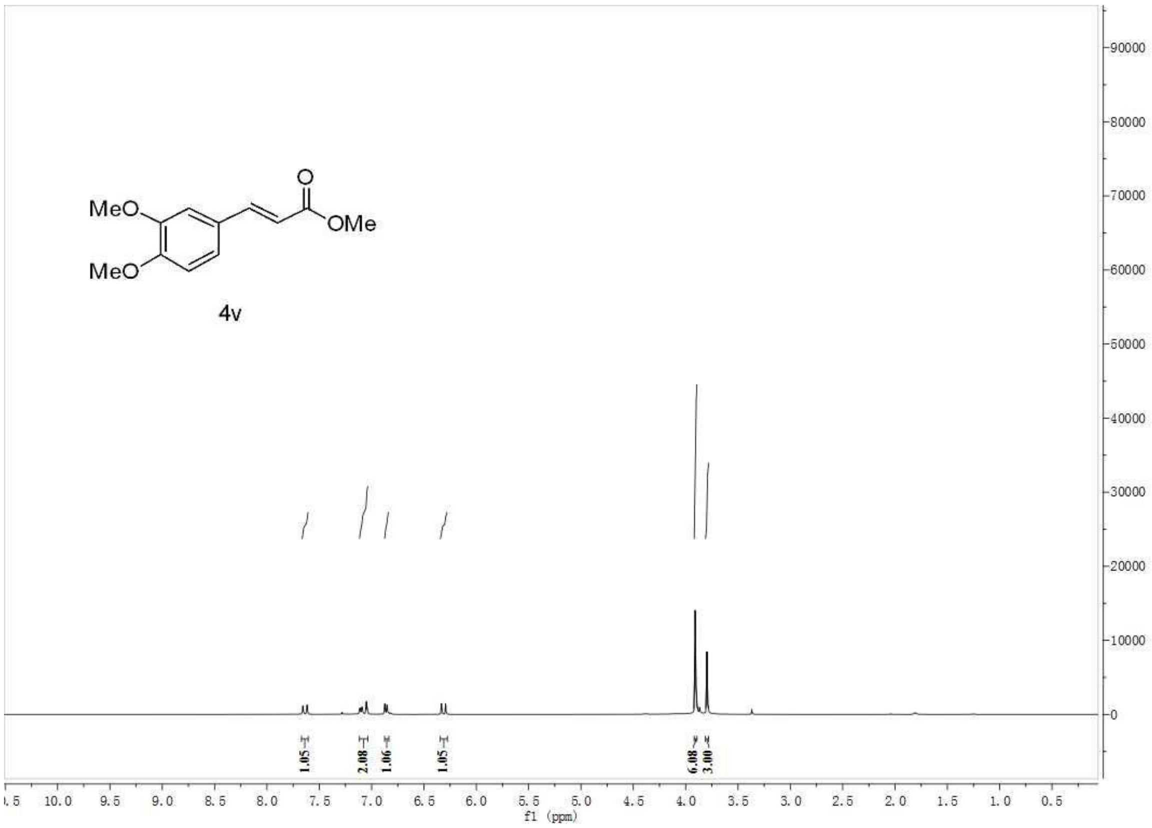

MeO
MeO
OMe
O
4v
1.05
2.08
1.06
1.05
6.08
3.00
f1 (ppm)

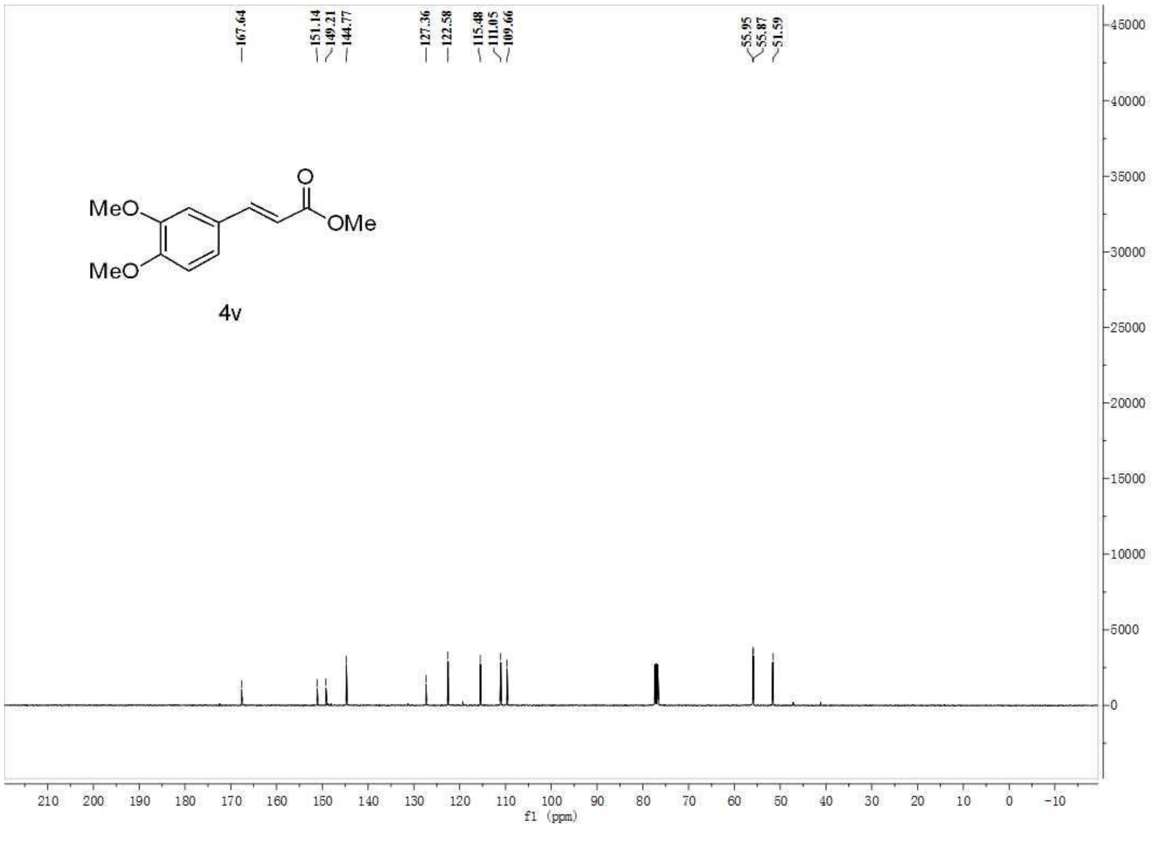

167.64
151.14
149.21
144.77
127.36
122.58
115.48
111.05
109.66
55.95
55.87
51.59
MeO
MeO
OMe
O
4v
f1 (ppm)

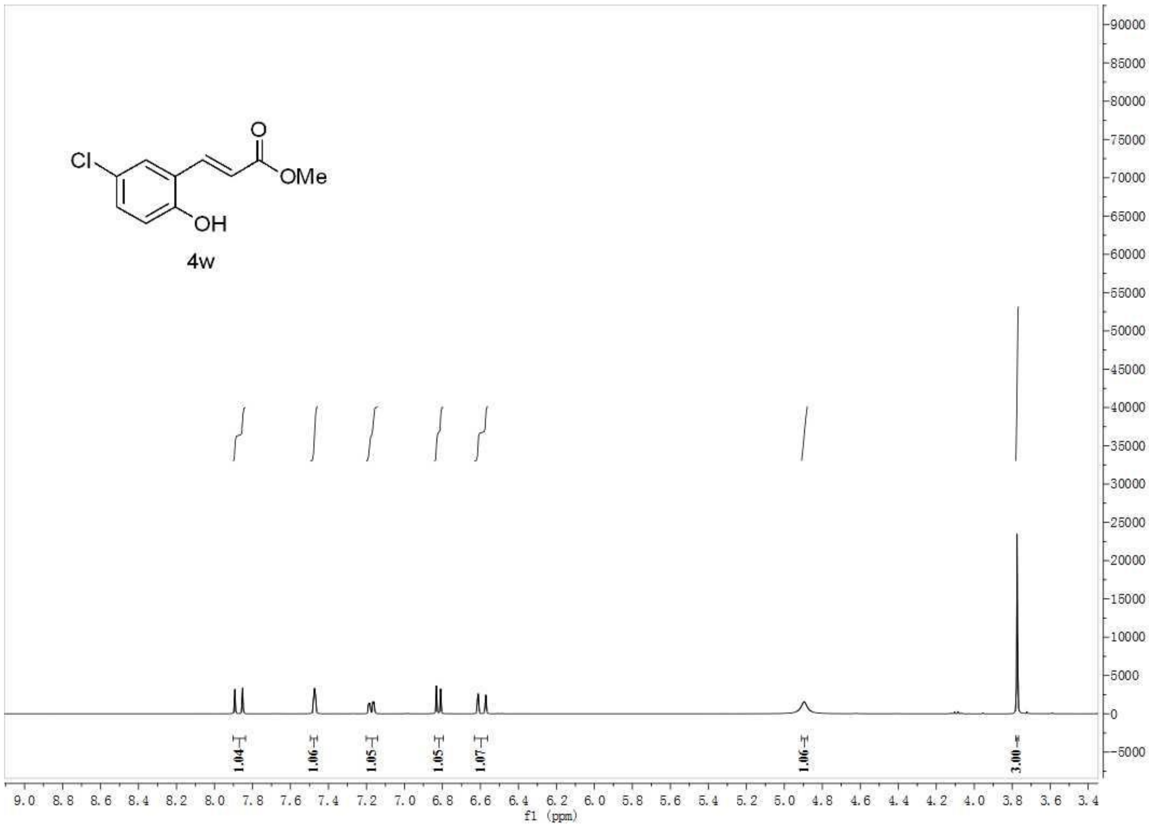
4w
1.04
1.06
1.05
1.05
1.07
1.06
3.00
f1 (ppm)

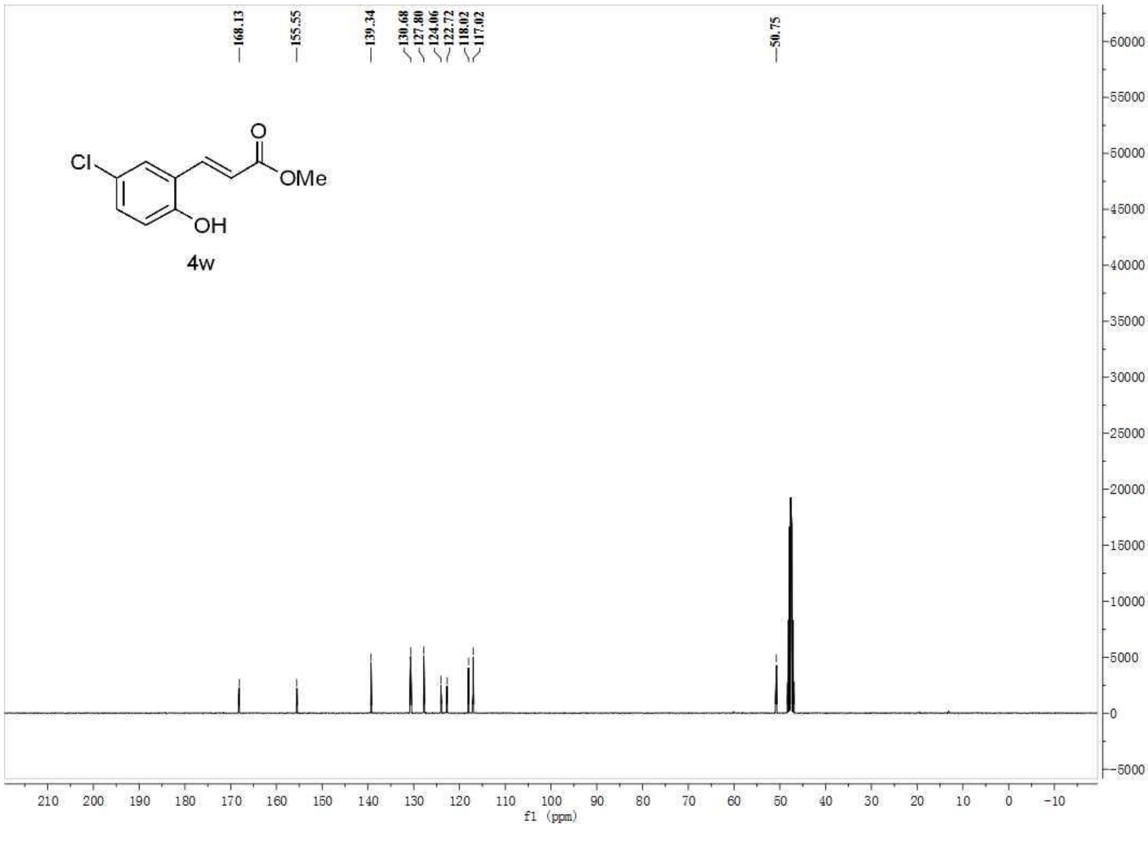
168.13
155.55
139.34
130.68
127.80
124.06
122.72
118.02
117.02
50.75
4w
f1 (ppm)

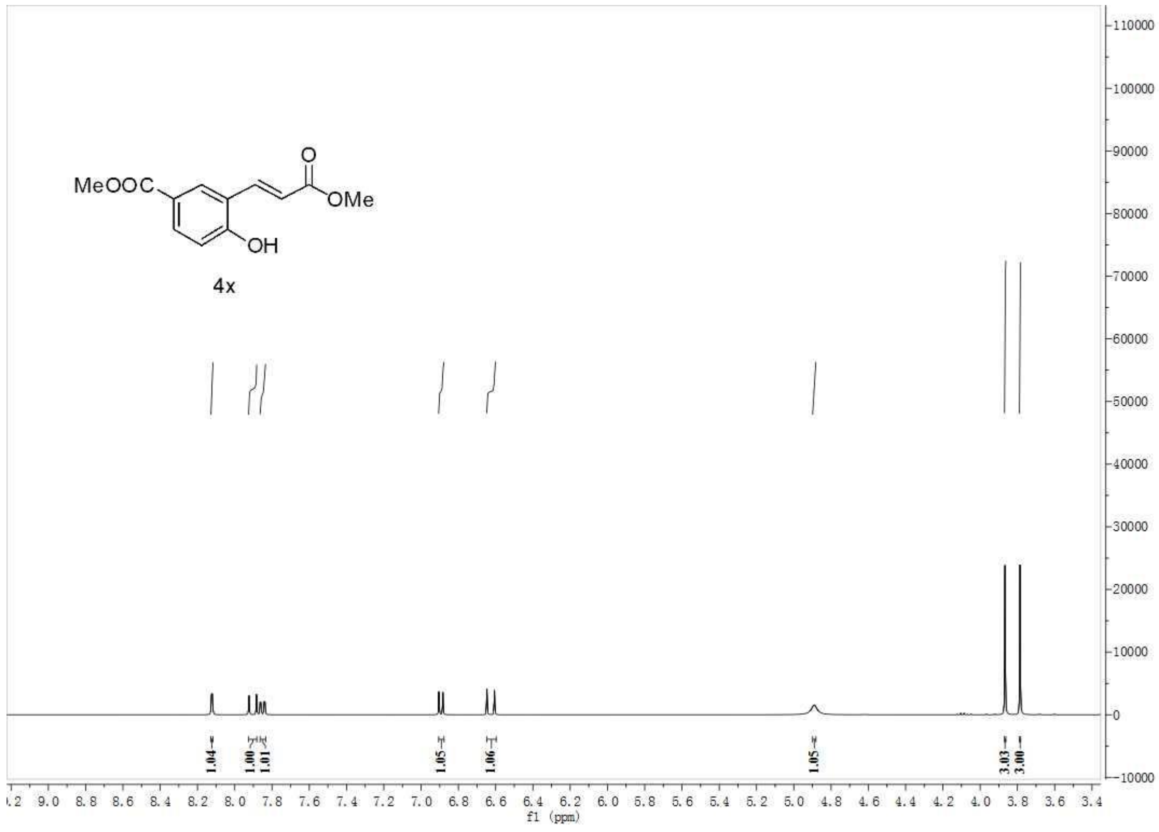
MeOOC
O
OMe
OH
4x
1.04
1.00
1.01
1.05
1.06
1.05
3.03
3.00
f1 (ppm)

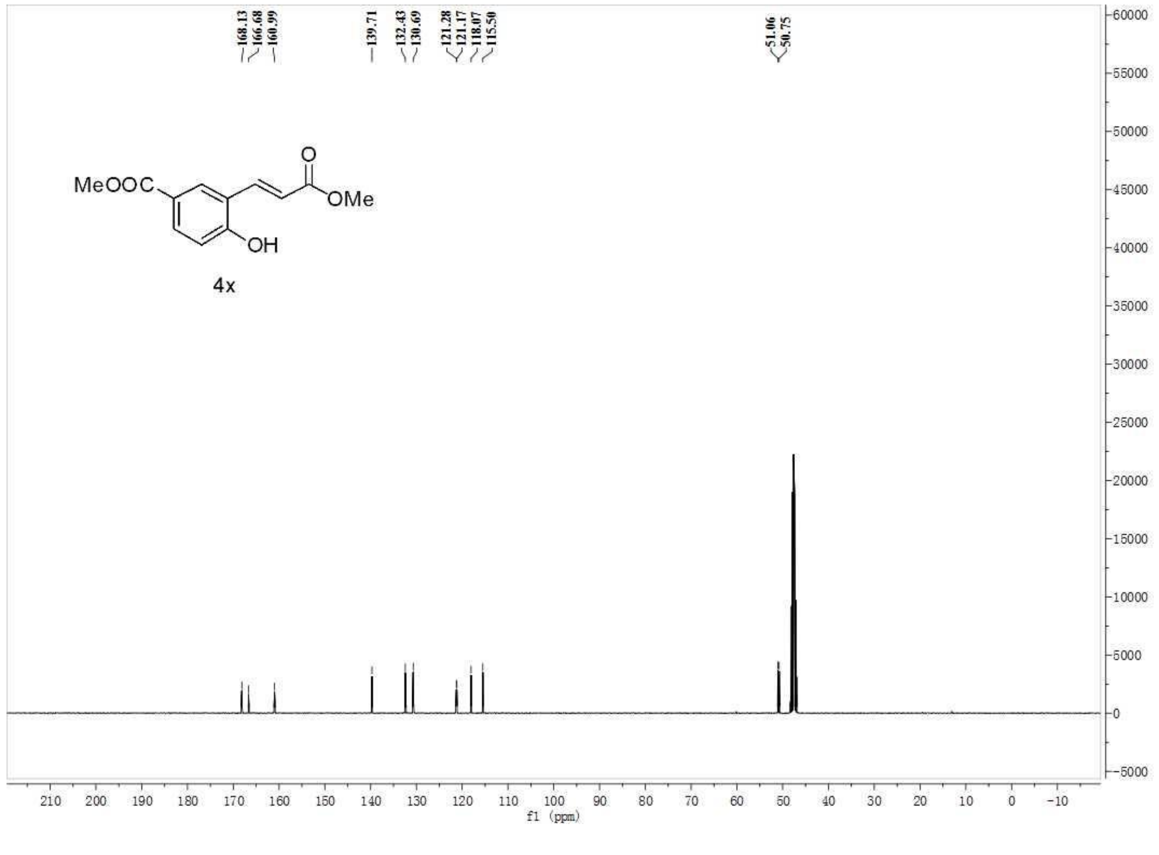
168.13
166.68
160.99
139.71
132.43
130.69
121.28
121.17
118.07
115.50
51.06
50.75
MeOOC
O
OMe
OH
4x
f1 (ppm)

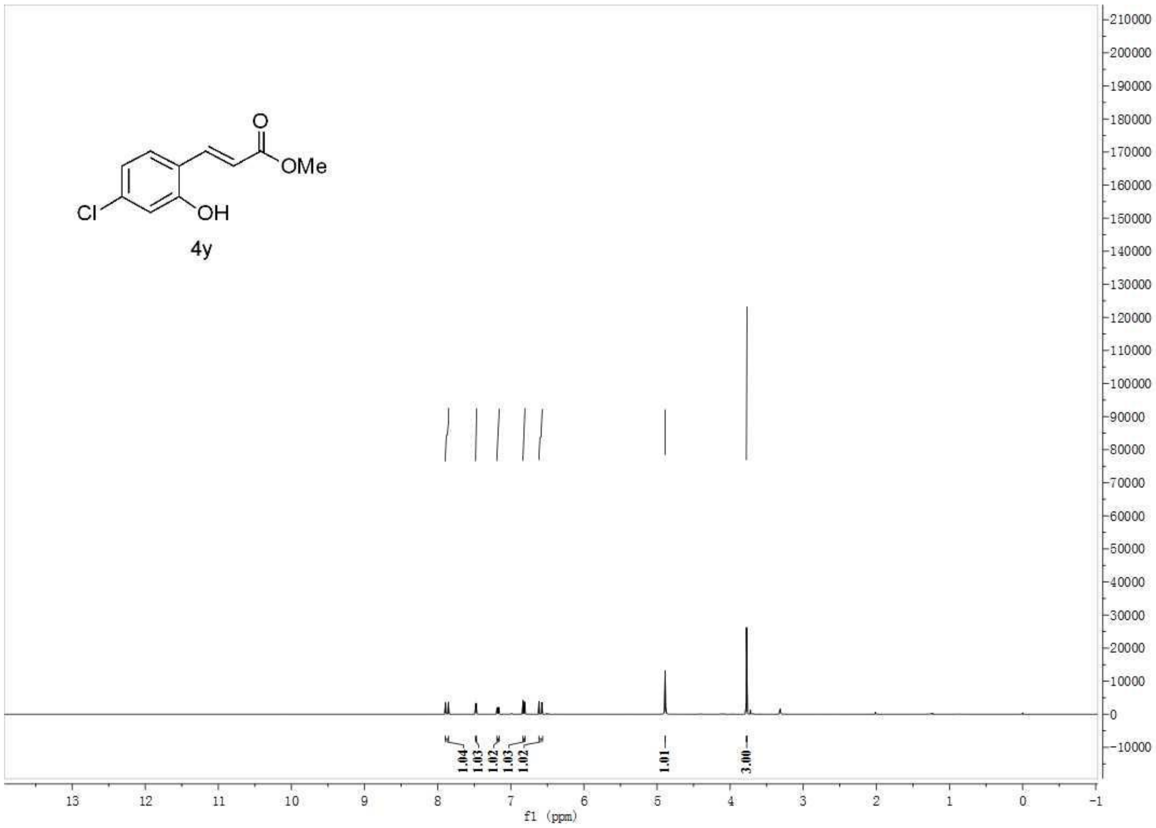
OMe
OH
Cl
4y
1.04
1.03
1.02
1.03
1.02
1.01
3.00
f1 (ppm)

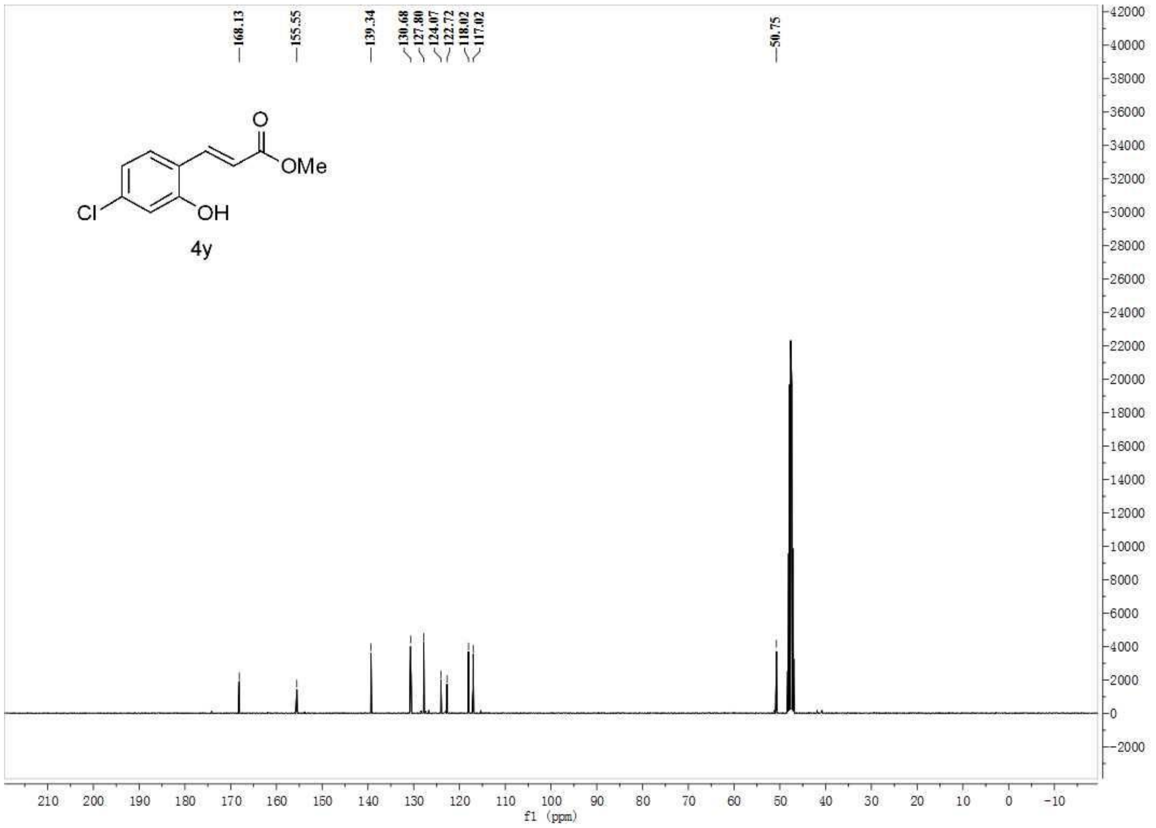
168.13
155.55
139.34
130.68
127.80
124.07
122.72
118.02
117.02
50.75
OMe
OH
Cl
4y
f1 (ppm)

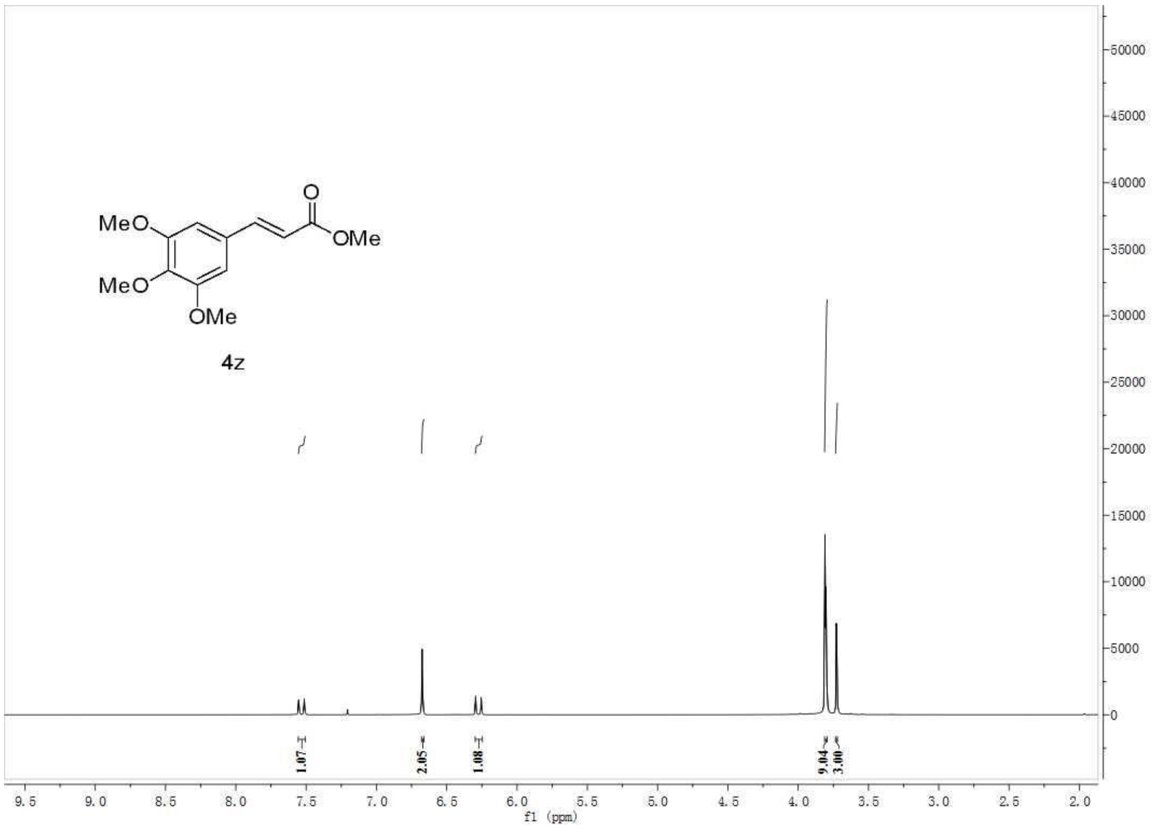
MeO
MeO
OMe
OMe
O
4z
1.07
2.05
1.08
9.04
3.00
f1 (ppm)

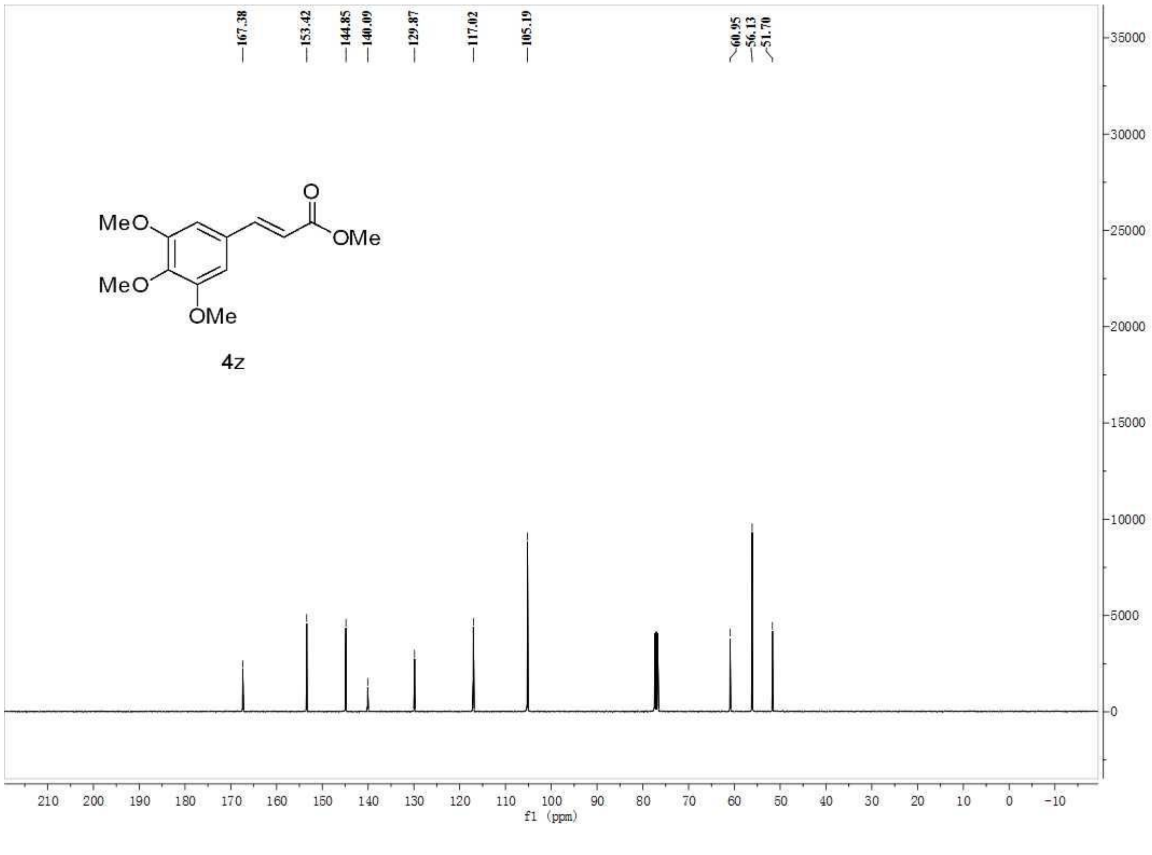
167.38
153.42
144.85
140.09
129.87
117.02
105.19
60.95
56.13
51.70
MeO
MeO
OMe
OMe
O
4z
f1 (ppm)

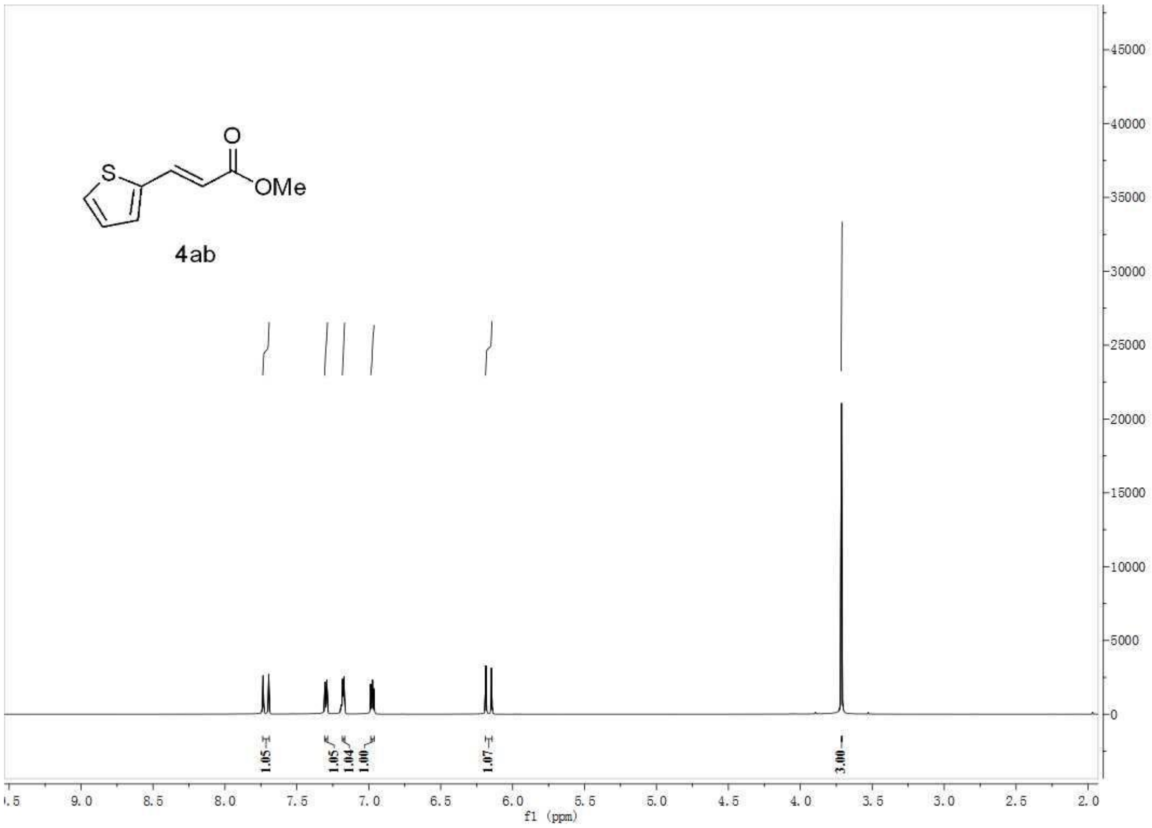
S
O
OMe
4ab
1.05
1.05
1.04
1.00
1.07
3.00
f1 (ppm)

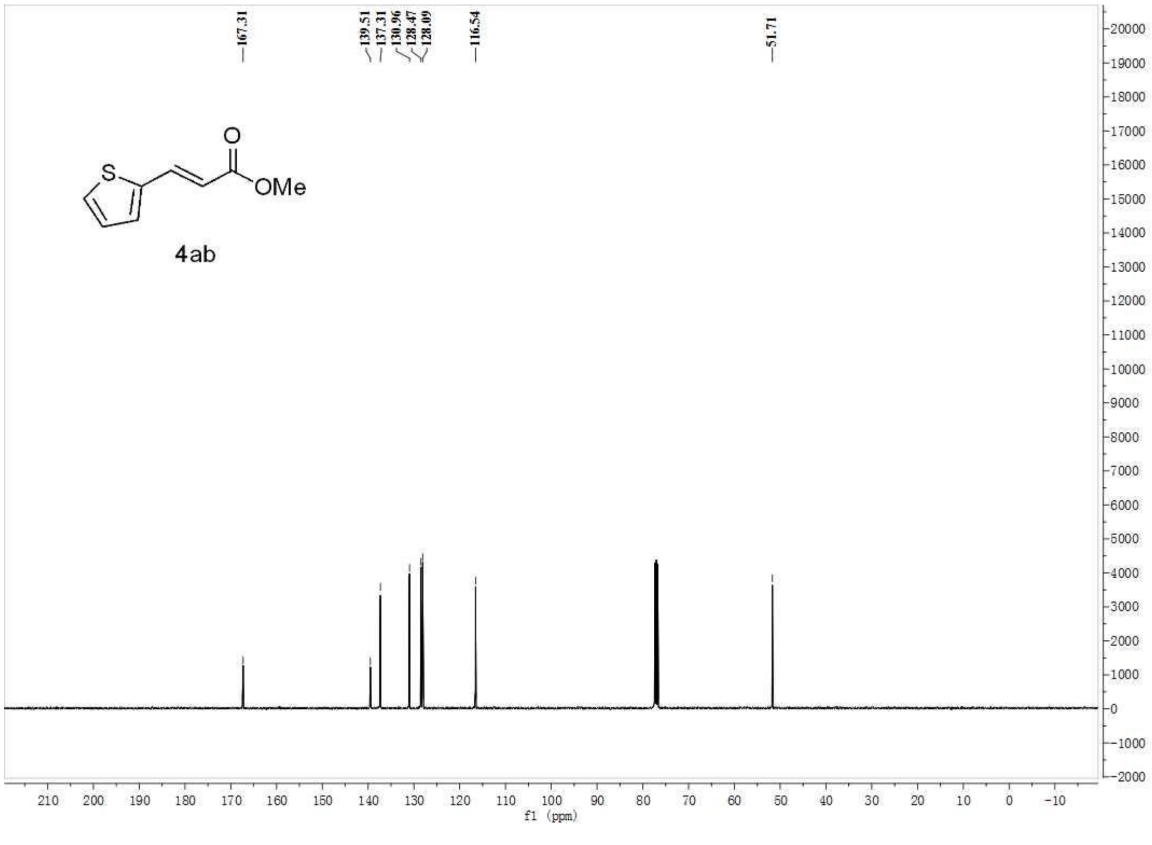
167.31
139.51
137.31
130.96
128.47
128.09
116.54
51.71
S
O
OMe
4ab
f1 (ppm)

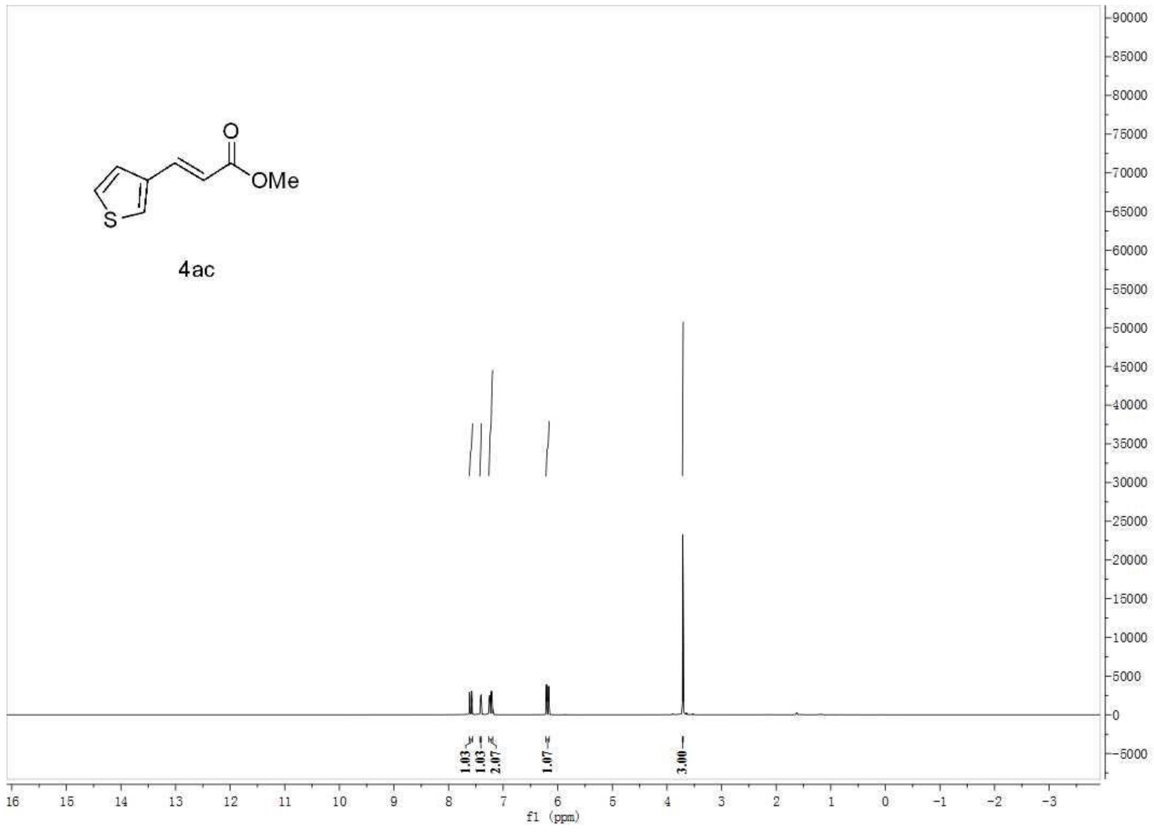
OMe
S
4ac
1.03
1.03
2.07
1.07
3.00
f1 (ppm)

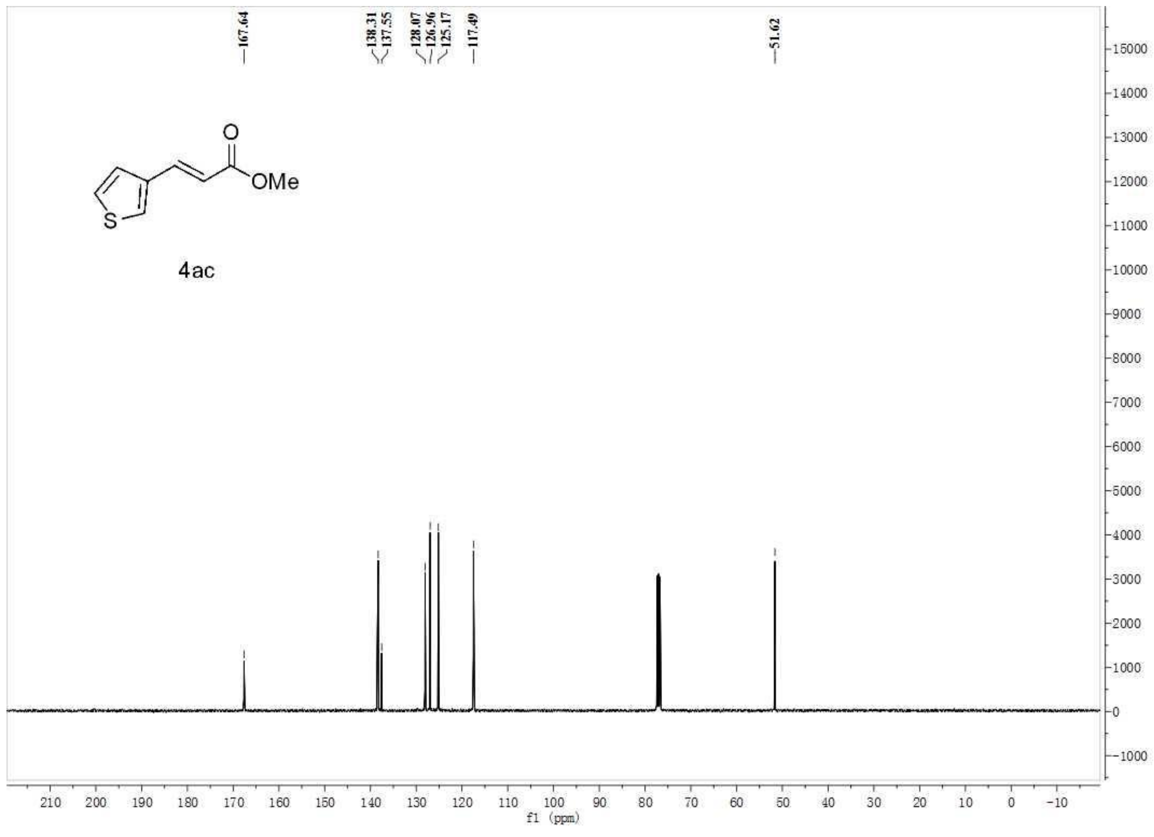
167.64
138.31
137.55
128.07
126.96
125.17
117.49
51.62
OMe
S
4ac
f1 (ppm)

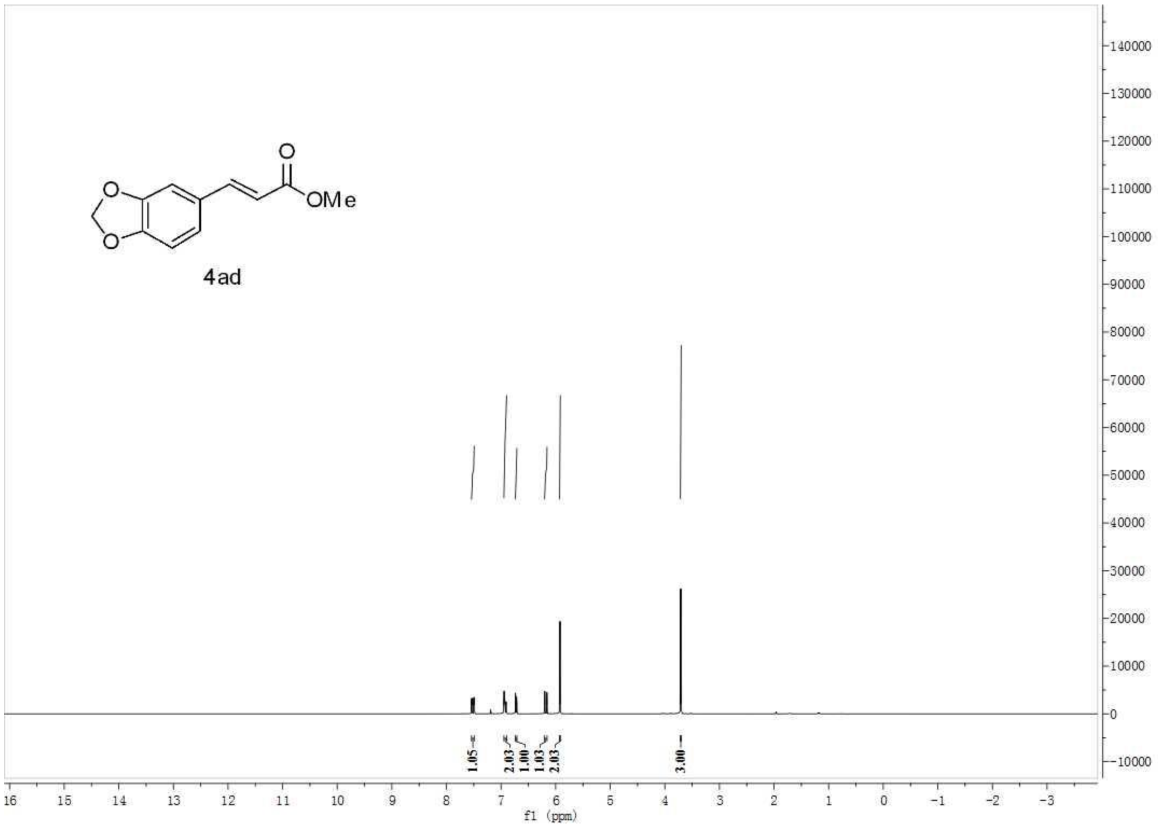
OMe
4ad
1.05
2.03
1.00
1.03
2.03
3.00
f1 (ppm)

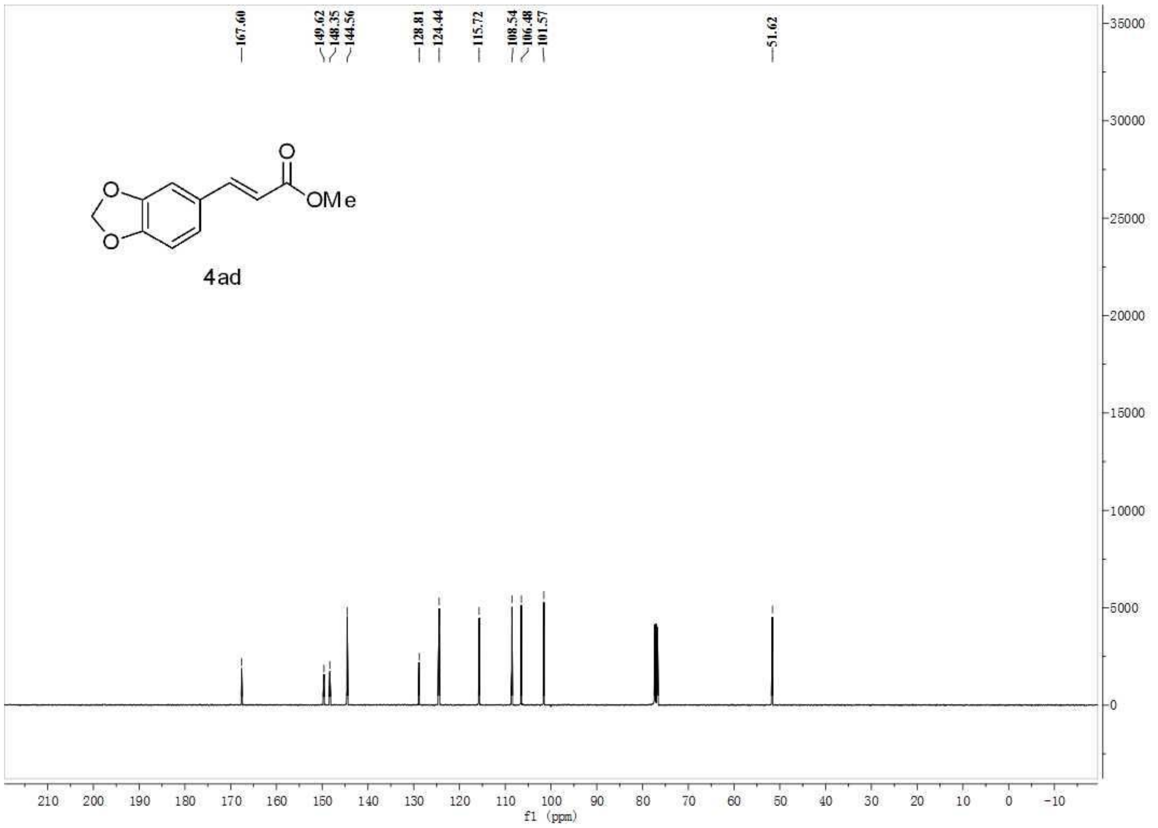
167.60
149.62
148.35
144.56
128.81
124.44
115.72
108.54
106.48
101.57
51.62
OMe
4ad
f1 (ppm)

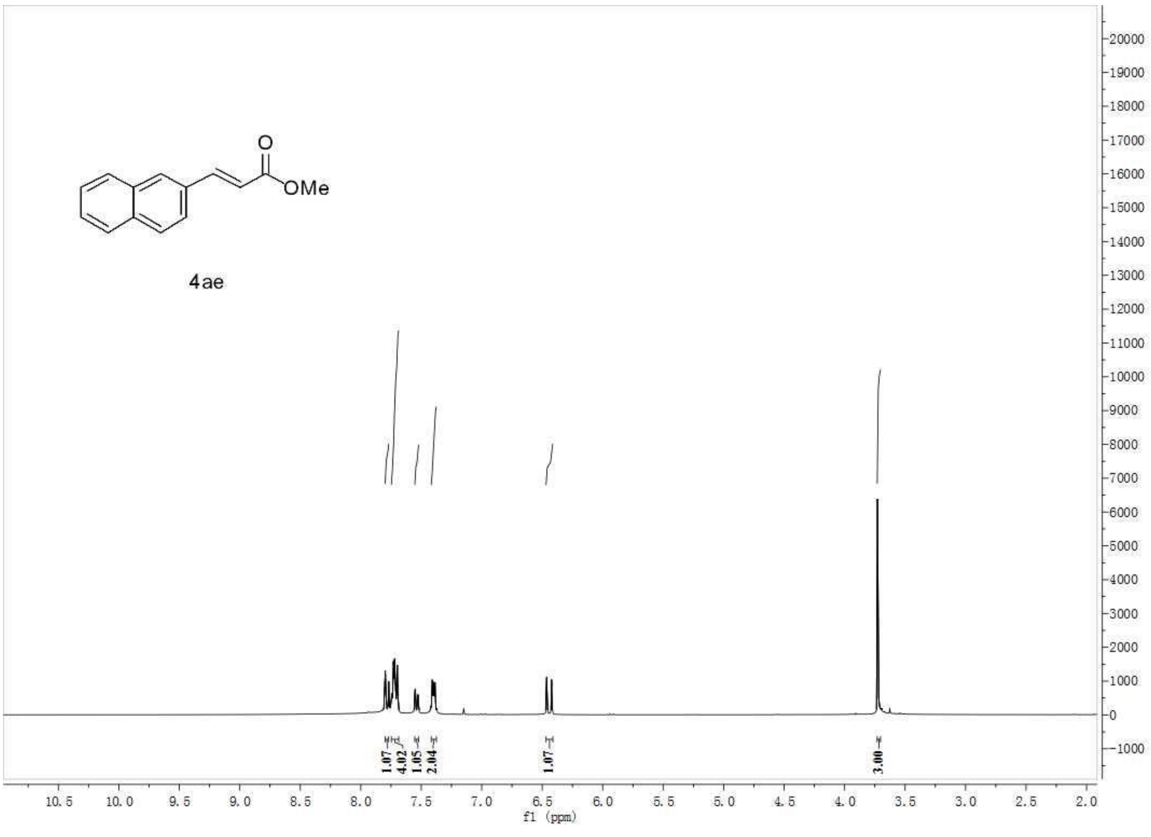

OMe
4ae
1.07
4.02
1.05
2.04
1.07
3.00
f1 (ppm)

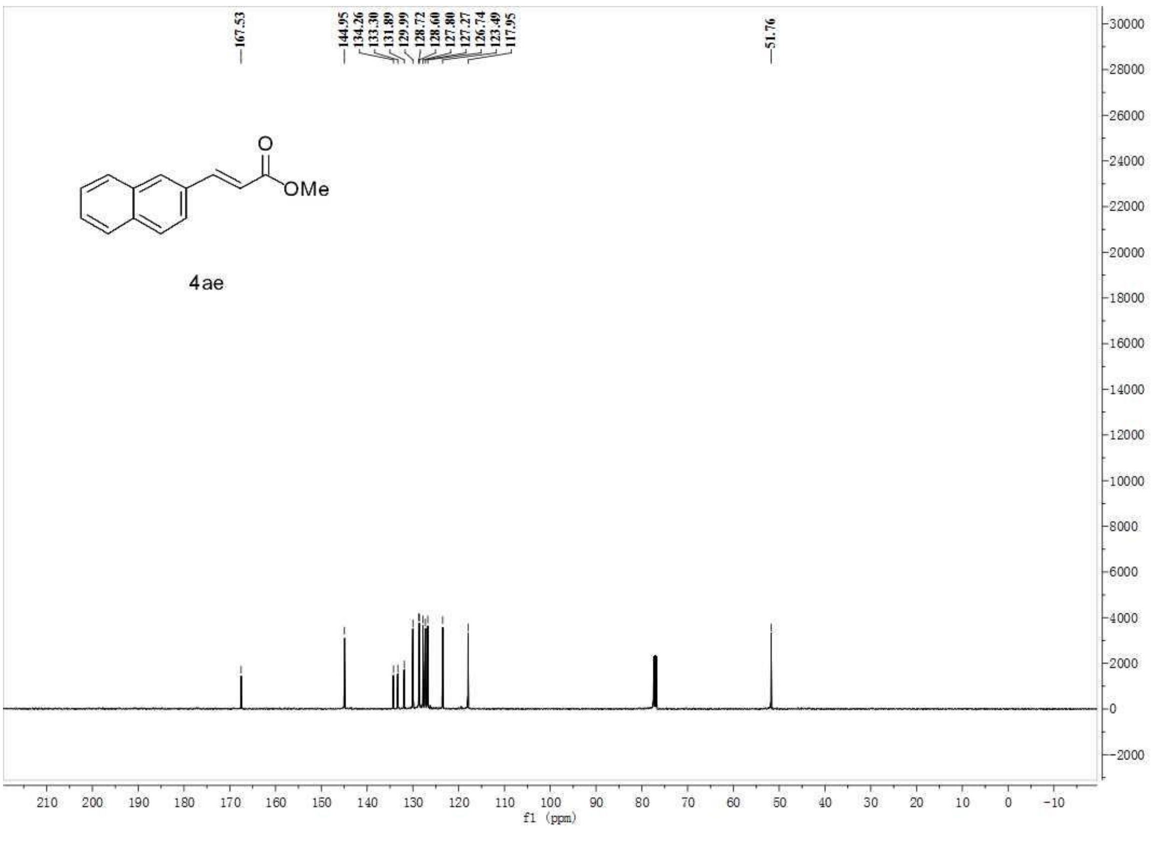

167.53
144.95
134.26
133.30
131.89
129.99
128.72
128.60
127.80
127.27
126.74
123.49
117.95
51.76
OMe
4ae
f1 (ppm)

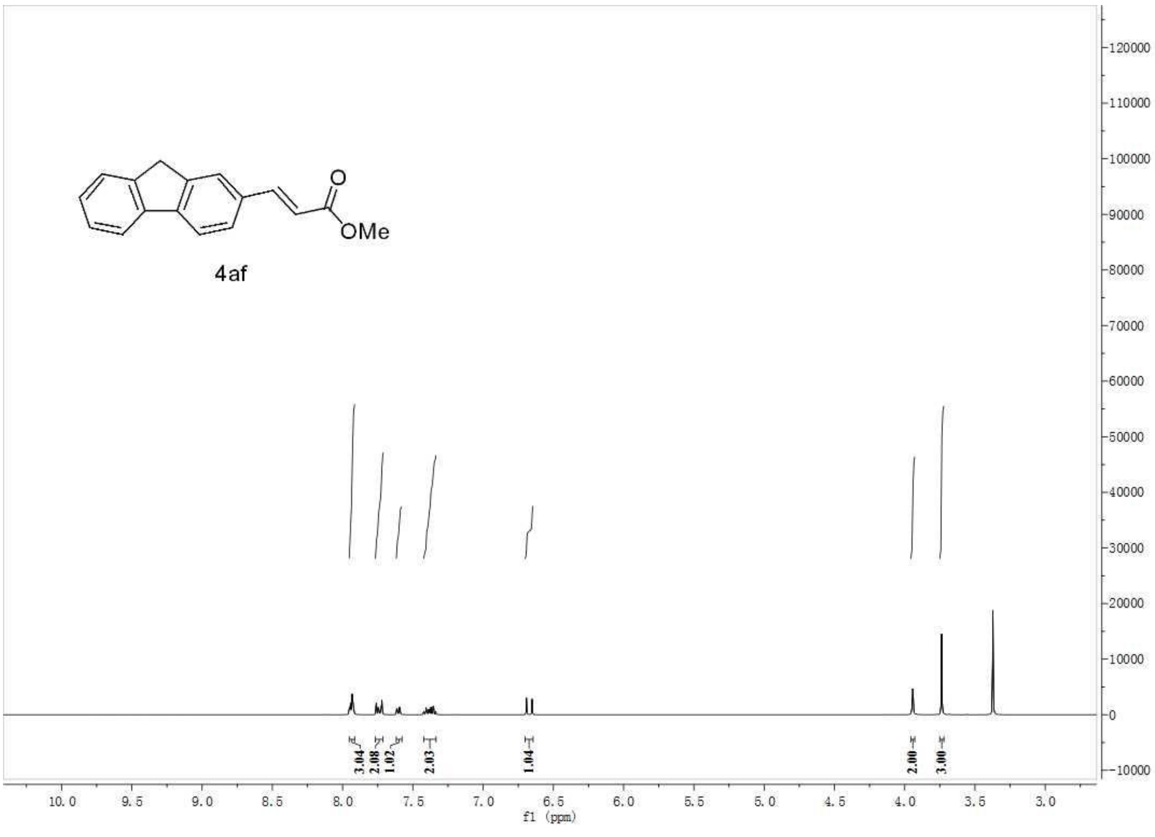
OMe
4af
3.04
2.08
1.02
2.03
1.04
2.00
3.00
f1 (ppm)

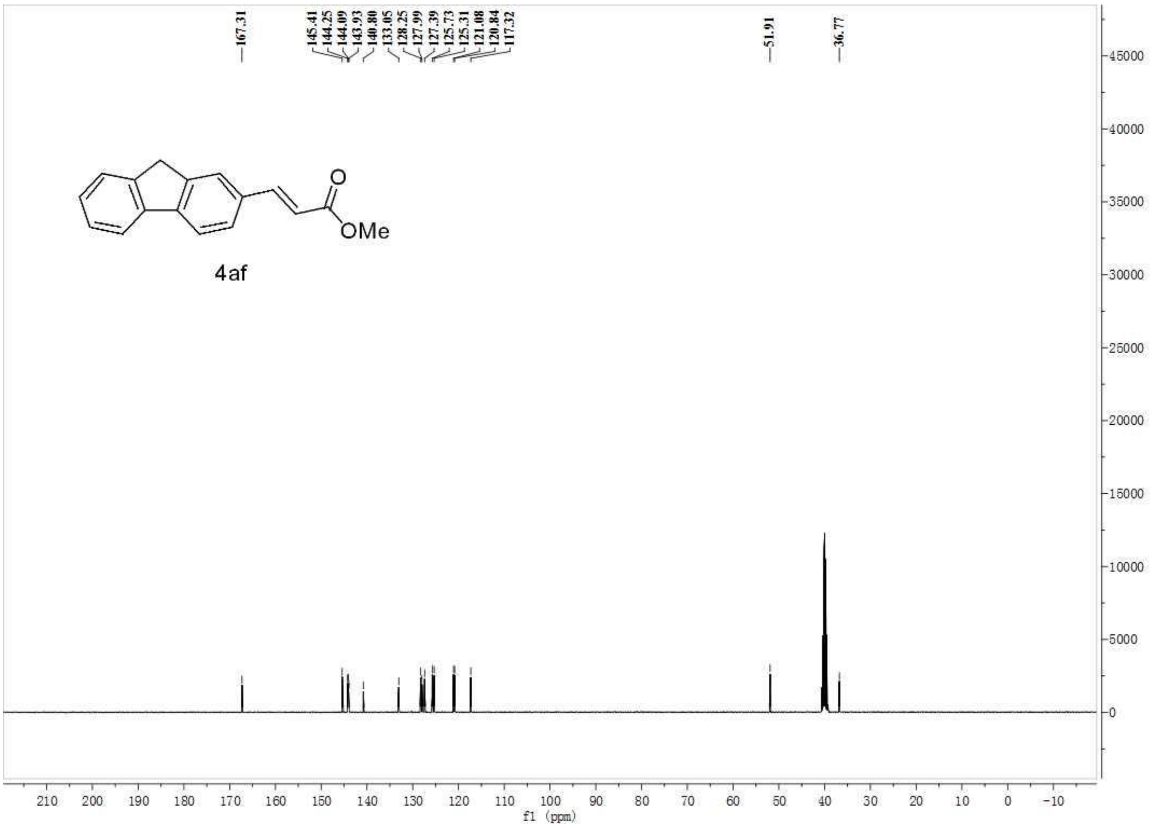
167.31
145.41
144.25
144.09
143.93
140.80
133.05
128.25
127.99
127.39
125.73
125.31
121.08
120.84
117.32
51.91
36.77
OMe
4af
f1 (ppm)

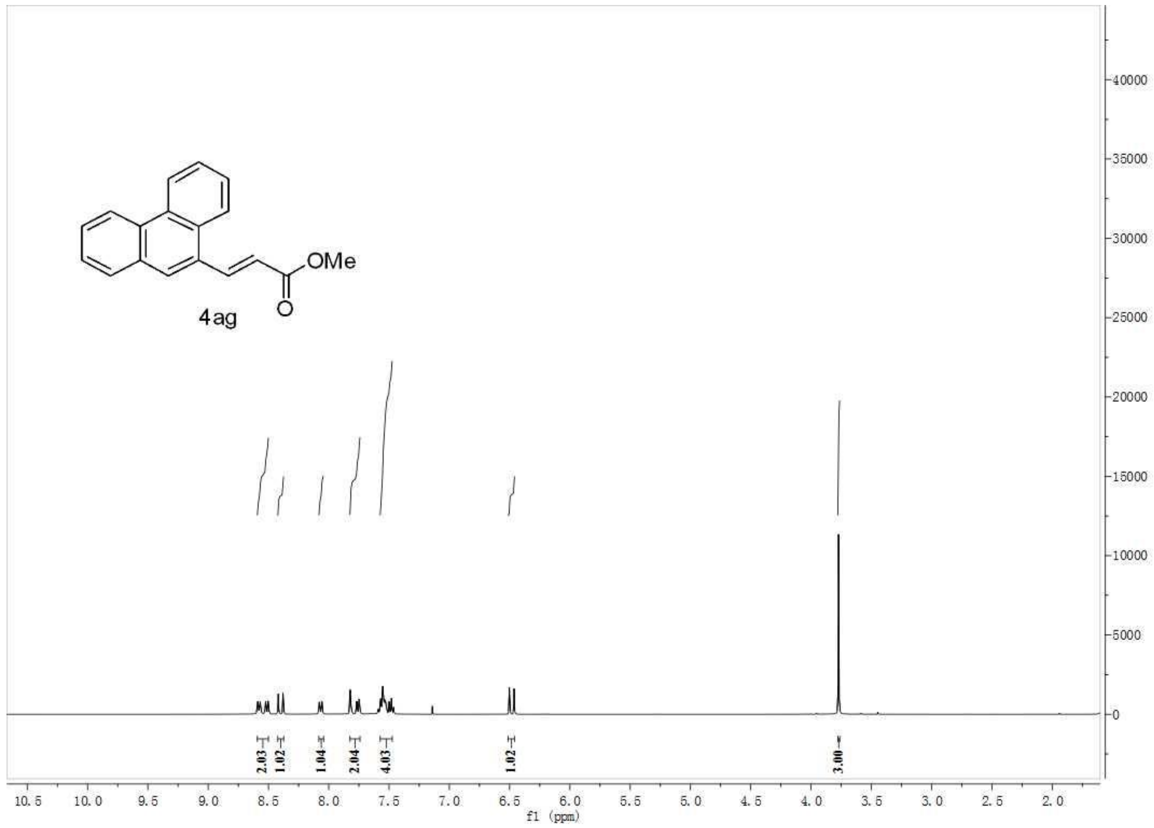
OMe
O
4ag
2.03
1.02
1.04
2.04
4.03
1.02
3.00
f1 (ppm)

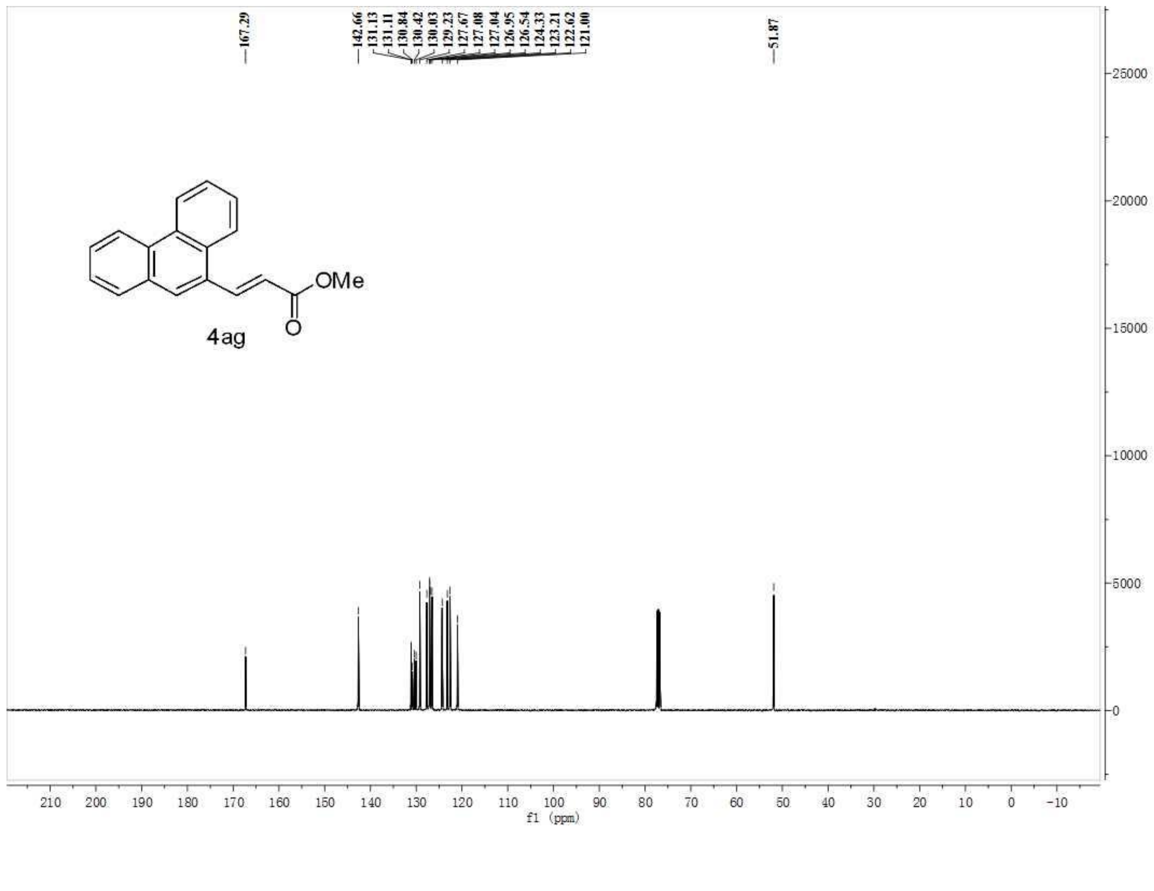
167.29
142.66
131.13
131.11
130.84
130.42
130.03
129.23
127.67
127.08
127.04
126.95
126.54
124.33
123.21
122.62
121.00
51.87
OMe
O
4ag
f1 (ppm)

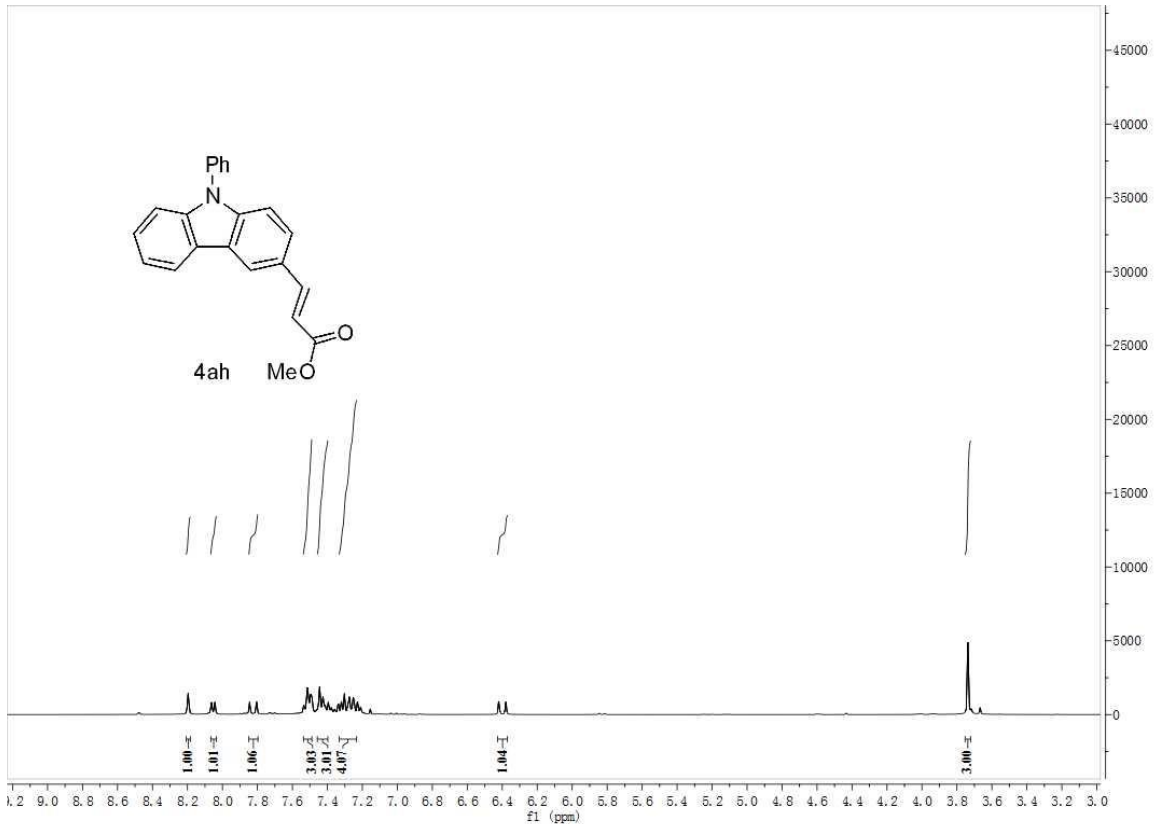
Ph
N
4ah
MeO
O
1.00
1.01
1.06
3.03
3.01
4.07
1.04
3.00
f1 (ppm)

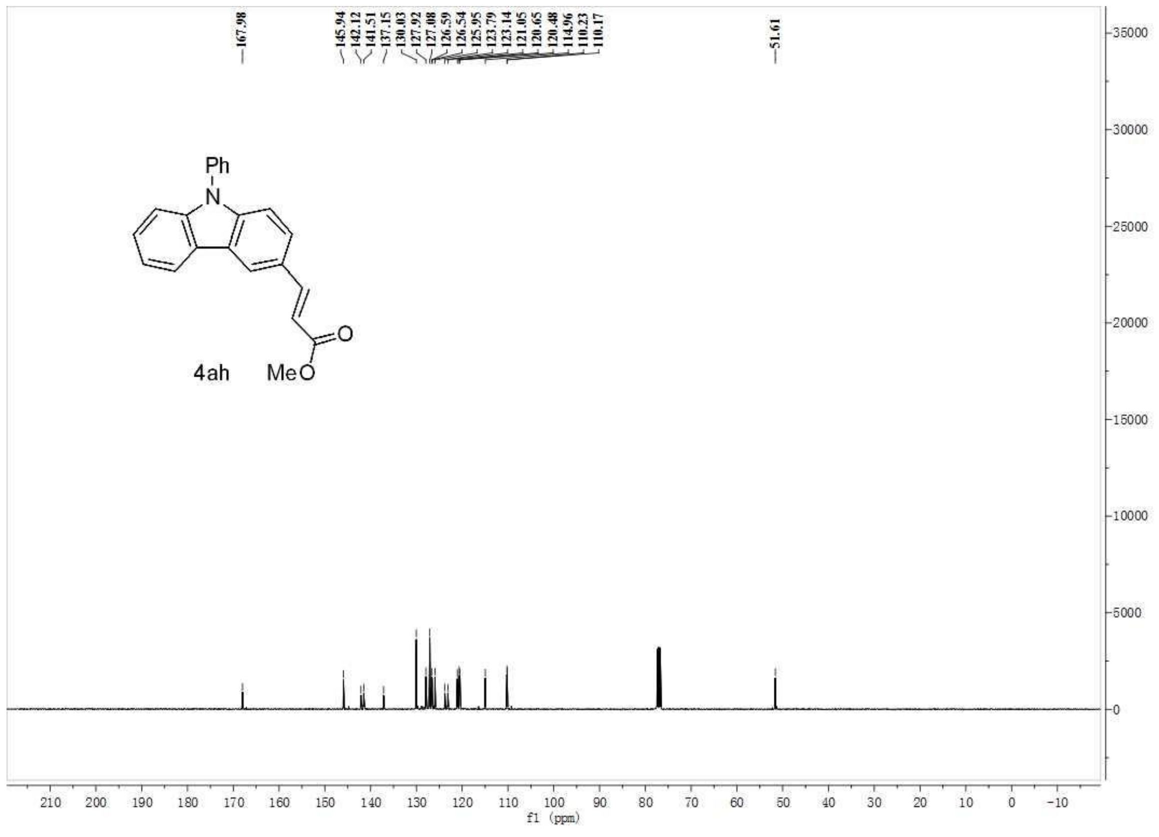
167.98
145.94
142.12
141.51
137.15
130.03
127.92
127.08
126.59
126.54
125.95
123.79
123.14
121.05
120.65
120.48
114.96
110.23
110.17
51.61
Ph
N
4ah
MeO
O
f1 (ppm)
